# Supplementary material for: FACED 2.0 enables large-scale voltage and calcium imaging in vivo
Source: Nat Methods. 2025 Dec 9;23(8):1611–21. doi: 10.1038/s41592-025-02925-7 (PMC13441978; doi:10.1038/s41592-025-02925-7)
Supplement: Supplementary file 1 [file 41592_2025_2925_MOESM1_ESM.pdf]

nature methods

Supplementary information https://doi.org/10.1038/s41592-025-02925-7

FACED 2.0 enables large-scale voltage and calcium imaging in vivo

In the format provided by the authors and unedited

This PDF file includes the following:

Supplementary Figures

Supplementary Figure 1. Representative -ΔF/F voltage traces (in blue) of all 182 neurons in Fig. 2.

Supplementary Figure 2. Raster plots of (top) spikes and (bottom) subthreshold -ΔF/F traces from 27 8.25-s trials of 182 neurons in Fig. 2.

Supplementary Figure 3. Representative -ΔF/F voltage traces (in blue) of all 225 neurons in Fig. 3.

Supplementary Figure 4. Raster plots of (top) spikes and (bottom) subthreshold -ΔF/F traces from 26 8.32-s trials of 225 neurons in Fig. 3.

Supplementary Figure 5. Simultaneously recorded ΔF/F calcium traces from 12,511 neurons during 36 64-s trials.

Supplementary Figure 6. Simultaneously recorded ΔF/F calcium traces from 14,005 neurons in during 51 trials.

Supplementary Figure 7. Example 10-trial-averaged ΔF/F calcium traces from calcium imaging datasets.

Supplementary Figure 8. FACED volumetric imaging accurately captures 3D structures in a 1,111 µm × 1,000 µm × 400 µm volume at 259.7 ms/vol without in-frame motion artifacts.

Supplementary Figure 9. FACED volumetric imaging accurately captures 3D structures in a 1,111 µm × 1,000 µm × 780 µm volume at 365.7 ms/vol without in-frame motion artifacts.

Supplementary Figure 10. Validation of the spike detection method on simulated voltage traces.

Supplementary Figure 11. Comparison of Pearson correlation coefficients (PCCs) between activity traces of neurons that are nearest neighbors along X axis (direction with potential contamination due to fluorescence lifetime) and Y axis (direction without the possibility of contamination).

Supplementary Notes

Supplementary Note 1: Additional characterization of volumetric imaging via continual Z movement of microscope objective

Supplementary Note 2: Evaluation of spike detection pipeline using simulated data

Supplementary Note 3: Voltage imaging at 800 Hz versus 400 Hz

Supplementary Note 4: Discussion on potential contamination due to fluorescence lifetime in activity imaging

Supplementary Note 5: FACED 2.0 versus FACED 1.0

Supplementary Tables

Supplementary Table 1: Comparison of 2PFM techniques

Supplementary Table 2: Parameters for 2D imaging with FACED 2.0

Supplementary Table 3: Parameters for 3D imaging with FACED 2.0

Supplementary Table 4: Brightness comparison for FACED 2.0 figures

Supplementary Table 5: Estimated numbers of photons in activity measurement datasets

Supplementary Table 6: Estimated baseline noise level for activity measurement datasets

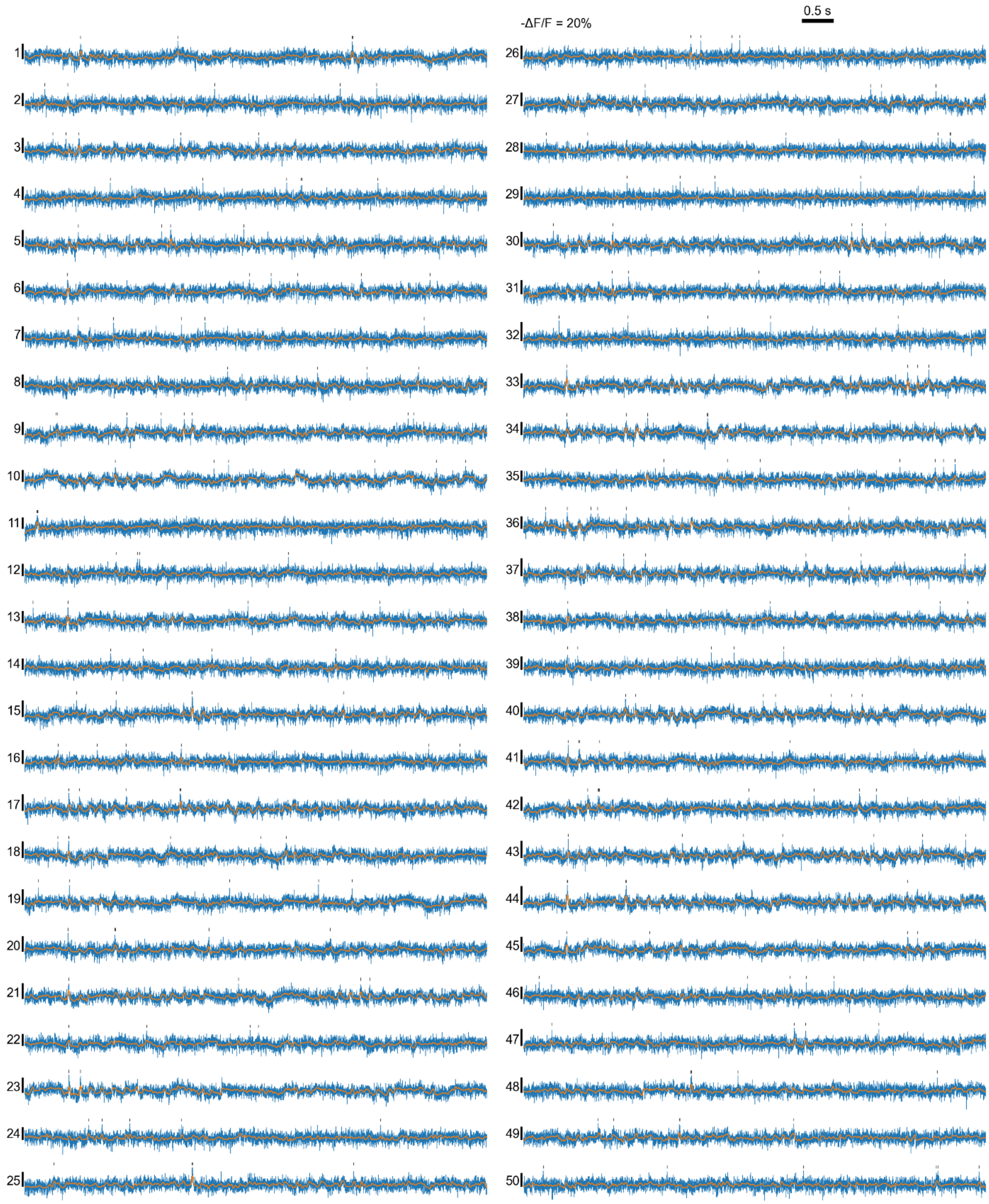
0.5 s
-ΔF/F = 20%
1
2
3
4
5
6
7
8
9
10
11
12
13
14
15
16
17
18
19
20
21
22
23
24
25
26
27
28
29
30
31
32
33
34
35
36
37
38
39
40
41
42
43
44
45
46
47
48
49
50

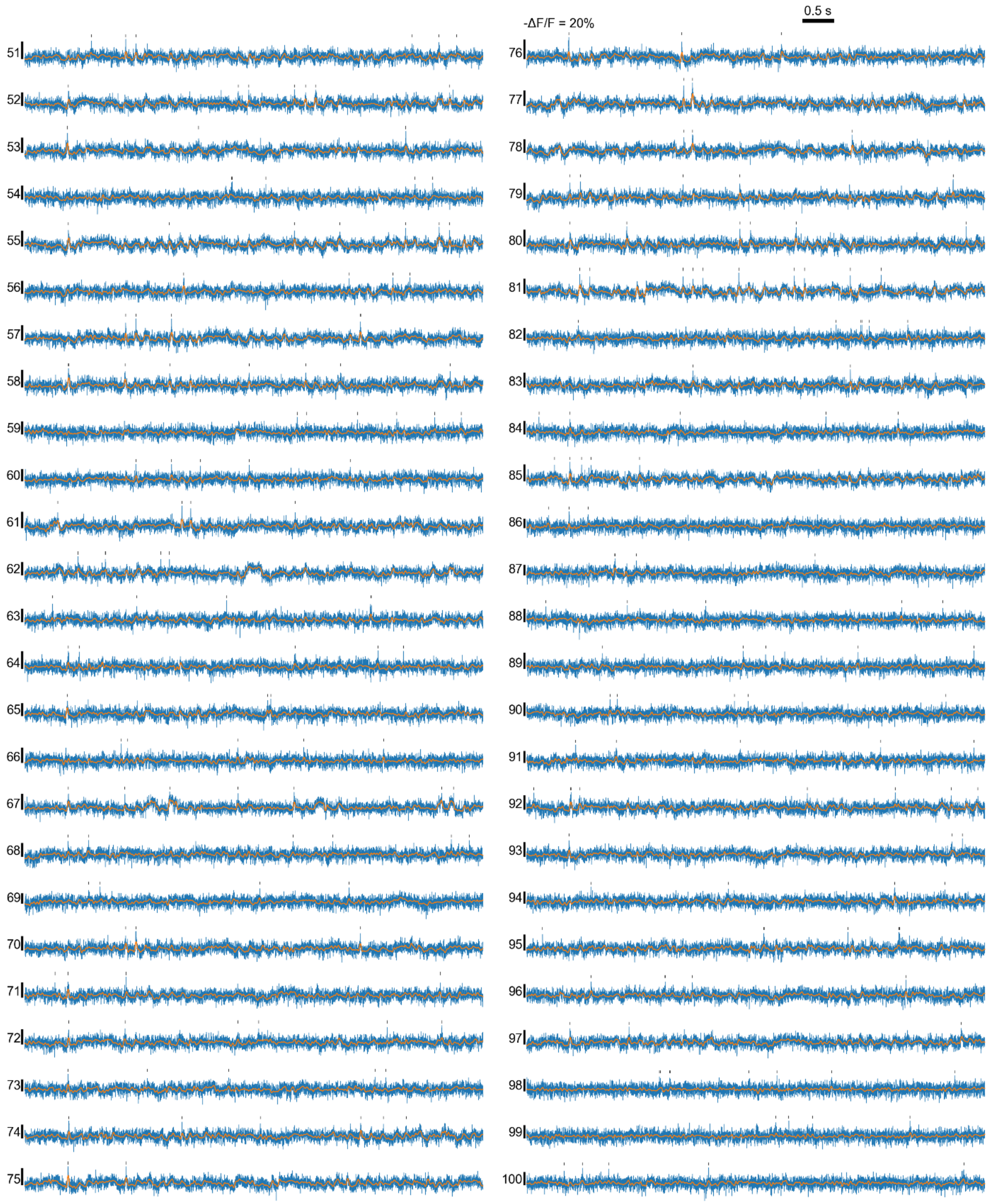

-ΔF/F = 20%
0.5 s
51
52
53
54
55
56
57
58
59
60
61
62
63
64
65
66
67
68
69
70
71
72
73
74
75
76
77
78
79
80
81
82
83
84
85
86
87
88
89
90
91
92
93
94
95
96
97
98
99
100

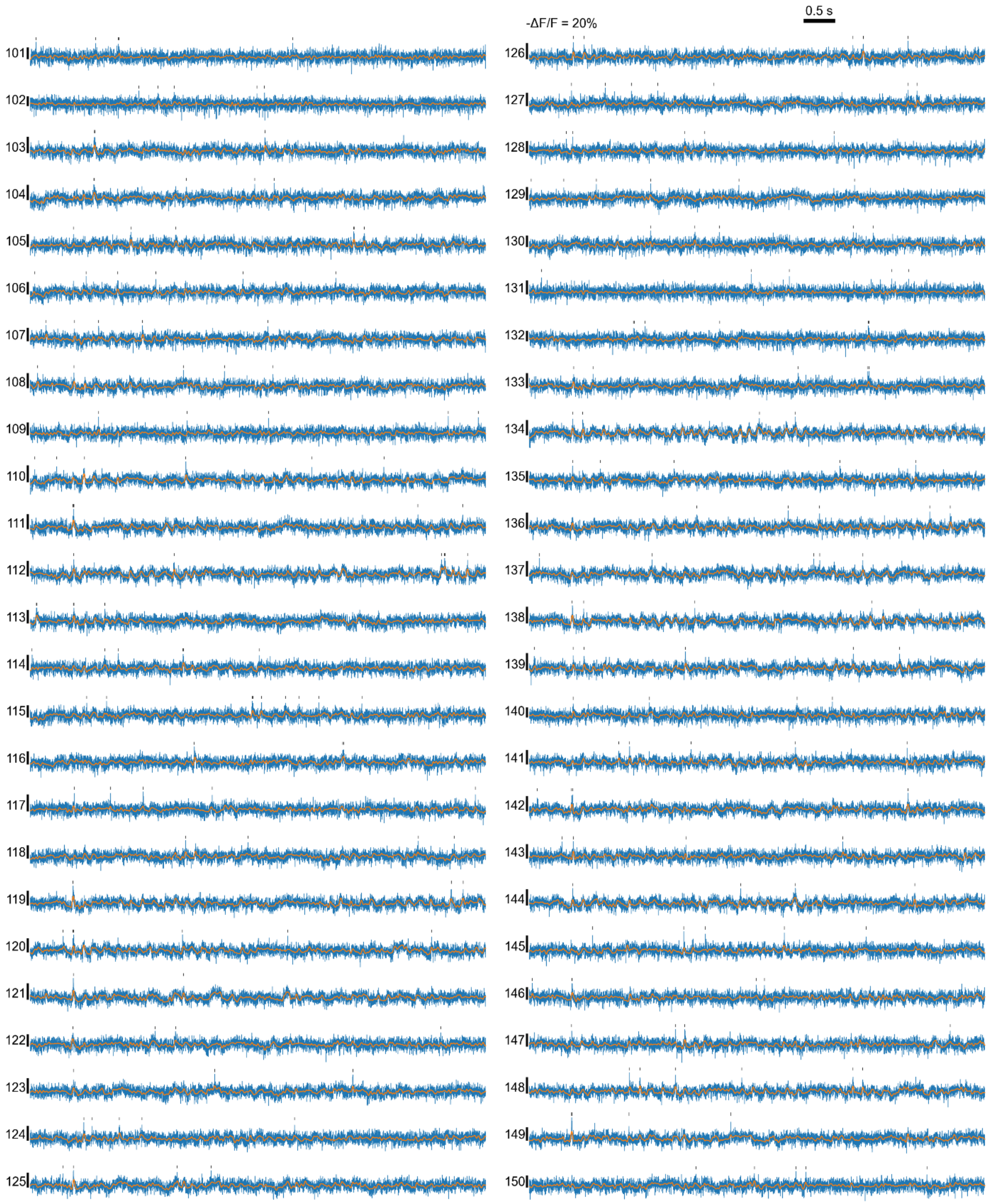

-ΔF/F = 20%
0.5 s
101
102
103
104
105
106
107
108
109
110
111
112
113
114
115
116
117
118
119
120
121
122
123
124
125
126
127
128
129
130
131
132
133
134
135
136
137
138
139
140
141
142
143
144
145
146
147
148
149
150

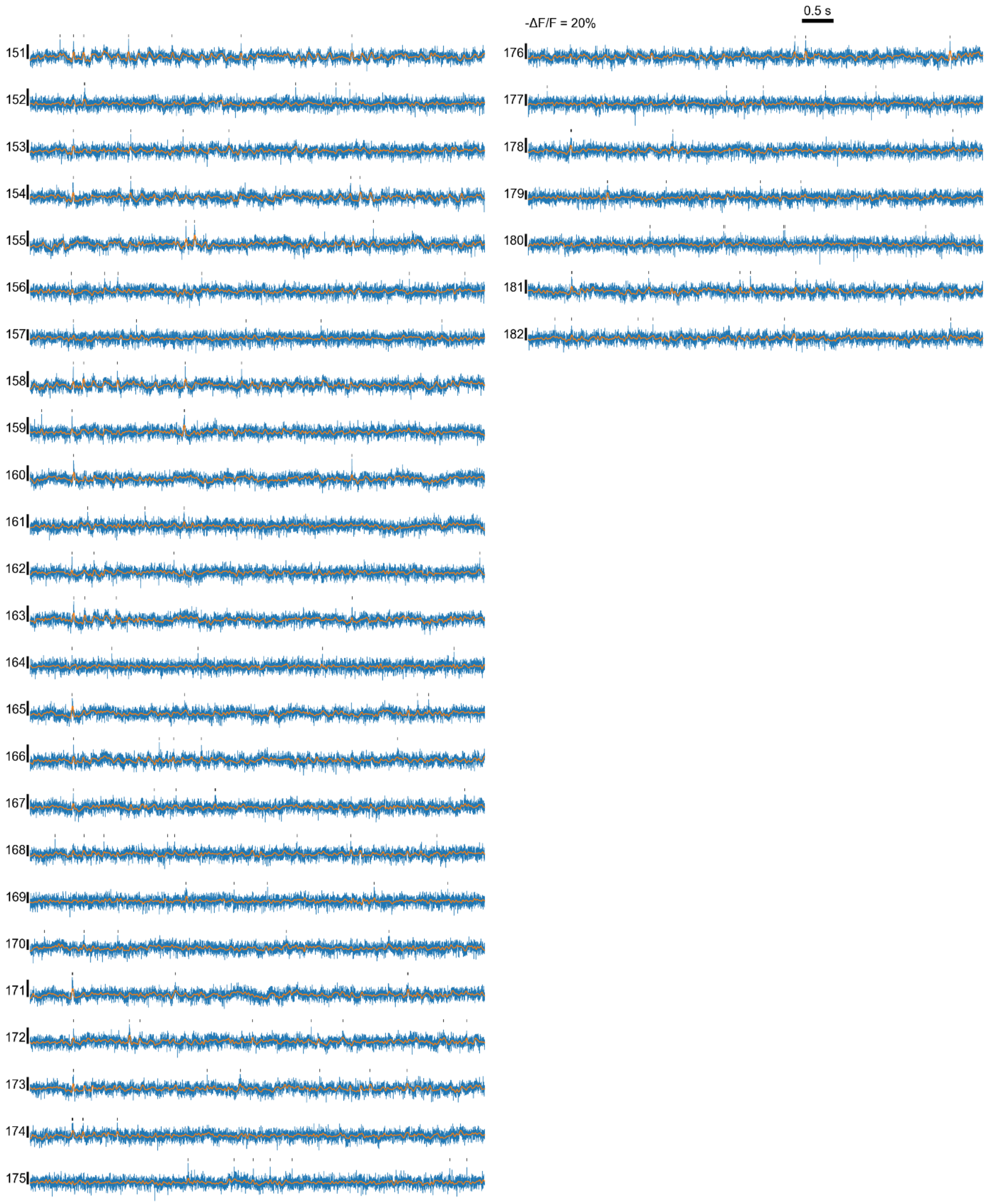

Supplementary Figure 1. Representative -ΔF/F voltage traces (in blue) of all 182 neurons in Fig. 2. Black ticks: suprathreshold spikes. Orange traces: extracted subthreshold -ΔF/F.

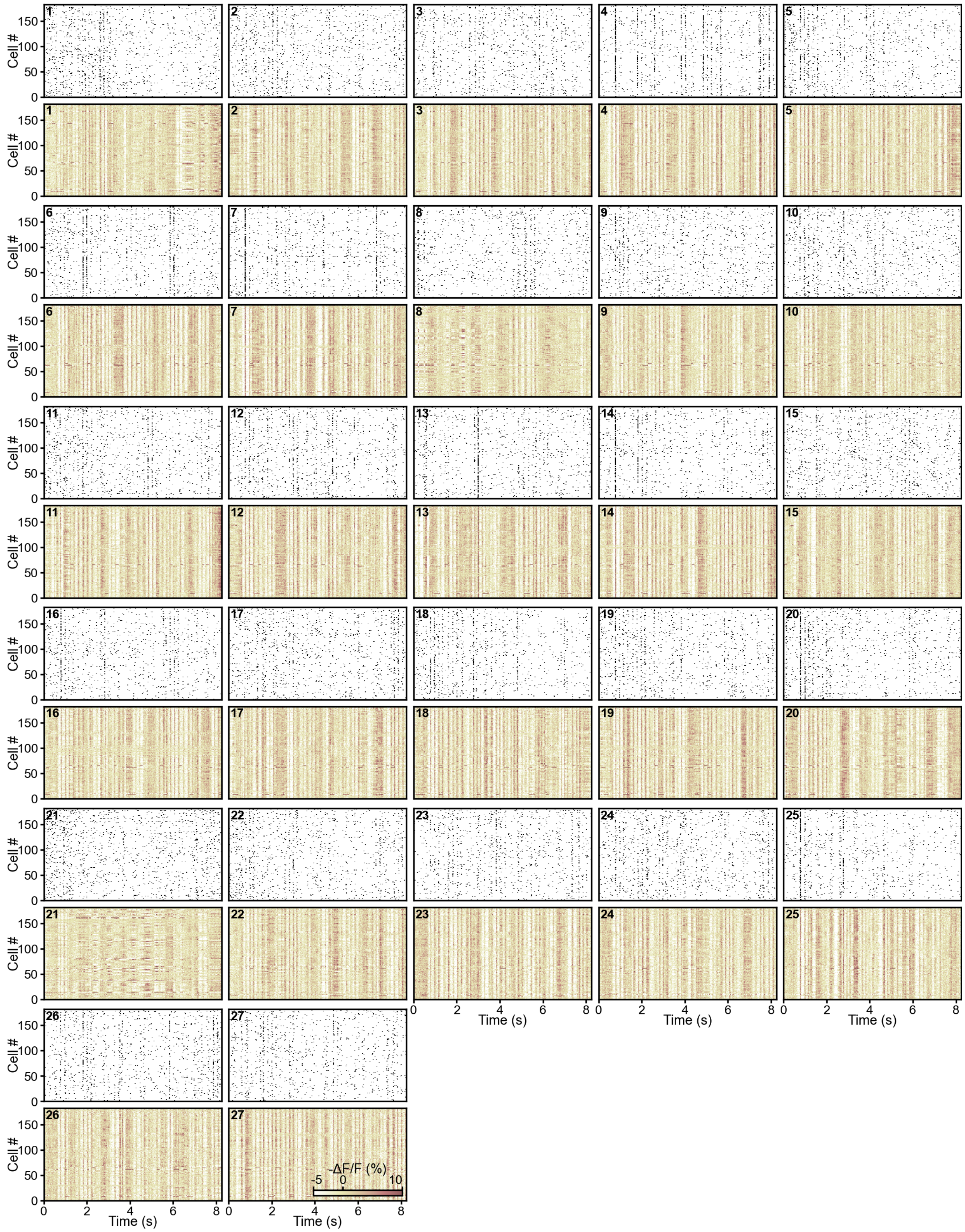

Supplementary Figure 2. Raster plots of (top) spikes and (bottom) subthreshold -ΔF/F traces from 27 8.25-s trials of 182 neurons in Fig. 2.

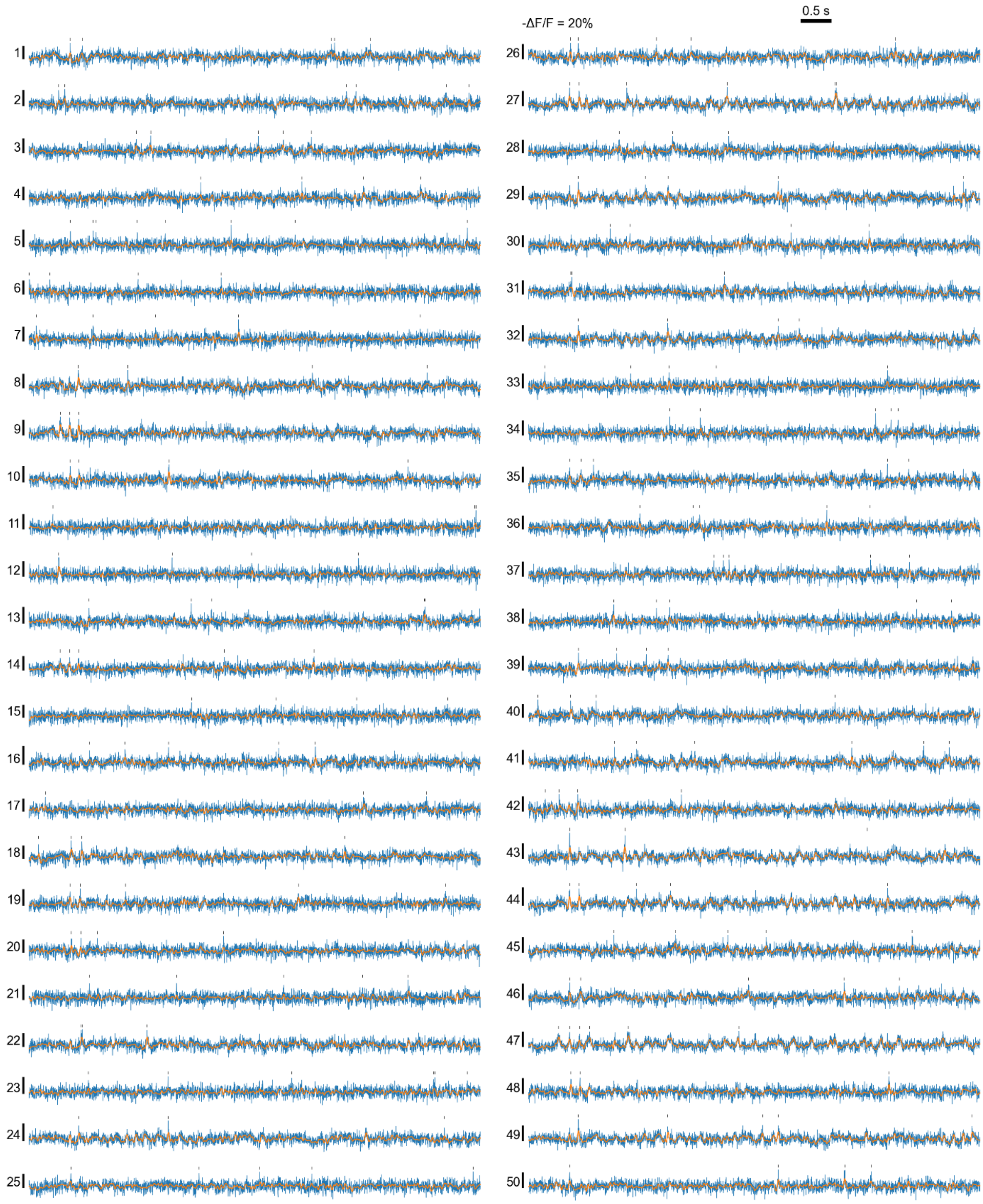

-ΔF/F = 20%
0.5 s
1
2
3
4
5
6
7
8
9
10
11
12
13
14
15
16
17
18
19
20
21
22
23
24
25
26
27
28
29
30
31
32
33
34
35
36
37
38
39
40
41
42
43
44
45
46
47
48
49
50

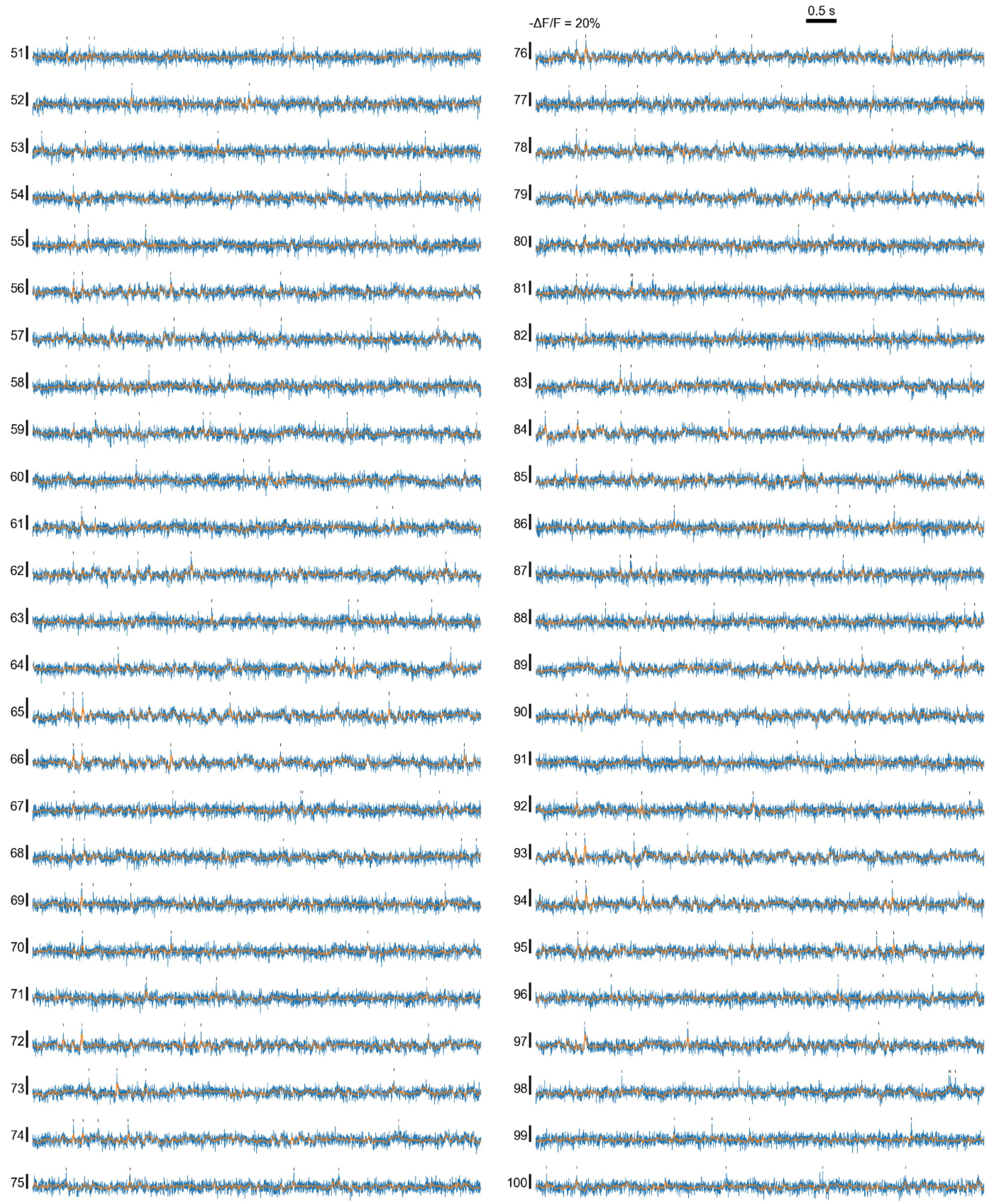
0.5 s
-ΔF/F = 20%
51
52
53
54
55
56
57
58
59
60
61
62
63
64
65
66
67
68
69
70
71
72
73
74
75
76
77
78
79
80
81
82
83
84
85
86
87
88
89
90
91
92
93
94
95
96
97
98
99
100

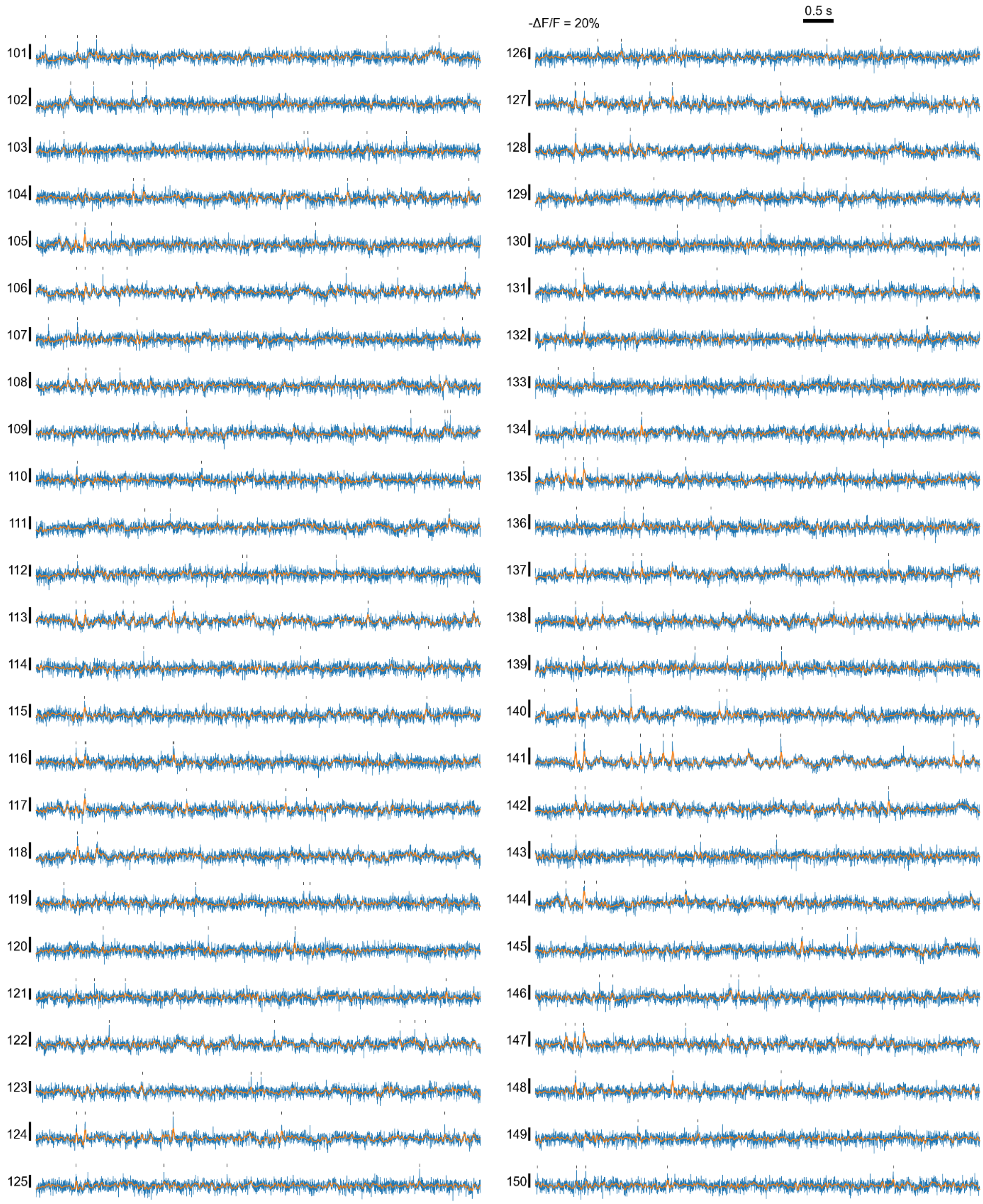

-ΔF/F = 20%
0.5 s
101
102
103
104
105
106
107
108
109
110
111
112
113
114
115
116
117
118
119
120
121
122
123
124
125
126
127
128
129
130
131
132
133
134
135
136
137
138
139
140
141
142
143
144
145
146
147
148
149
150

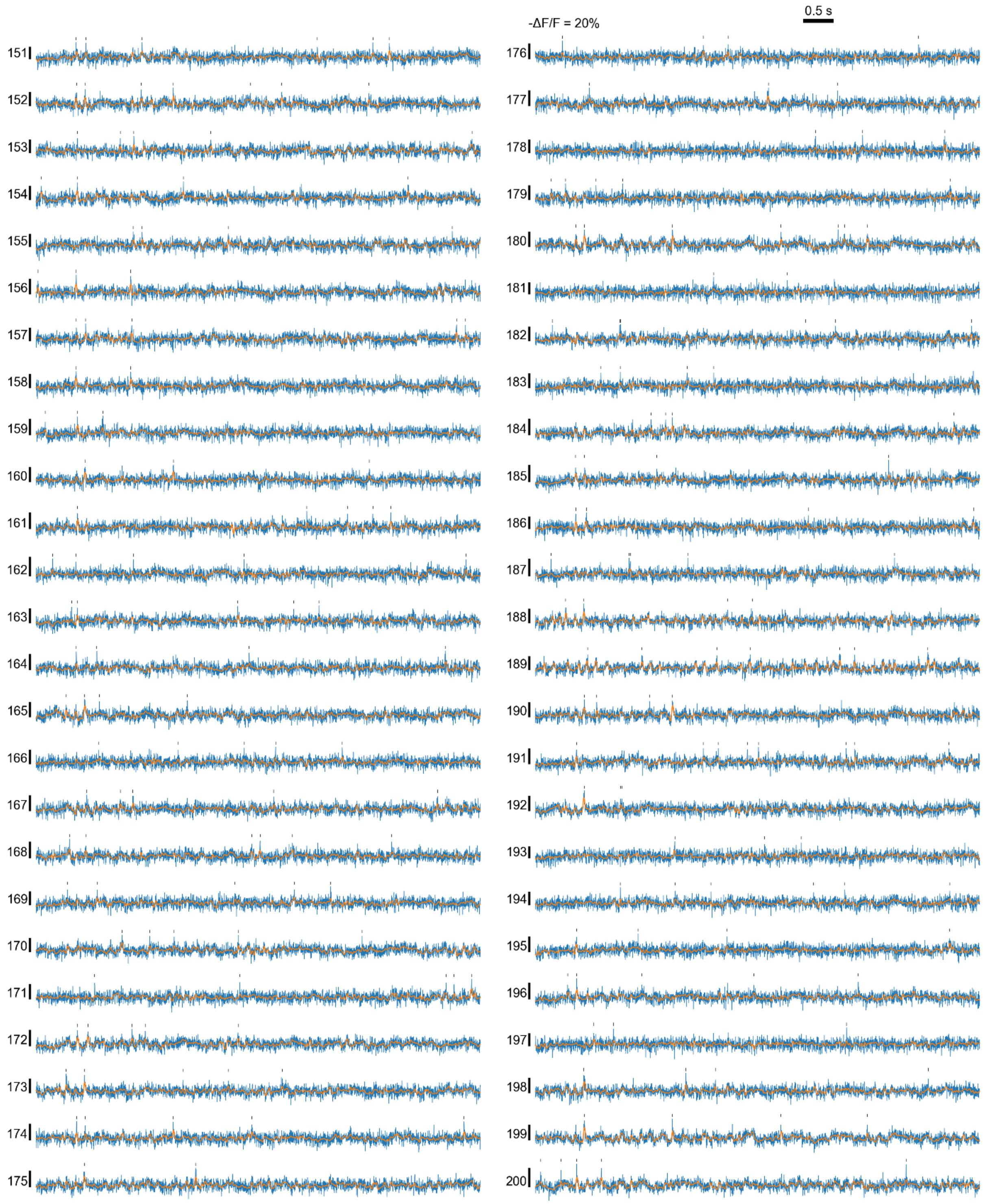

0.5 s
-ΔF/F = 20%
151
152
153
154
155
156
157
158
159
160
161
162
163
164
165
166
167
168
169
170
171
172
173
174
175
176
177
178
179
180
181
182
183
184
185
186
187
188
189
190
191
192
193
194
195
196
197
198
199
200

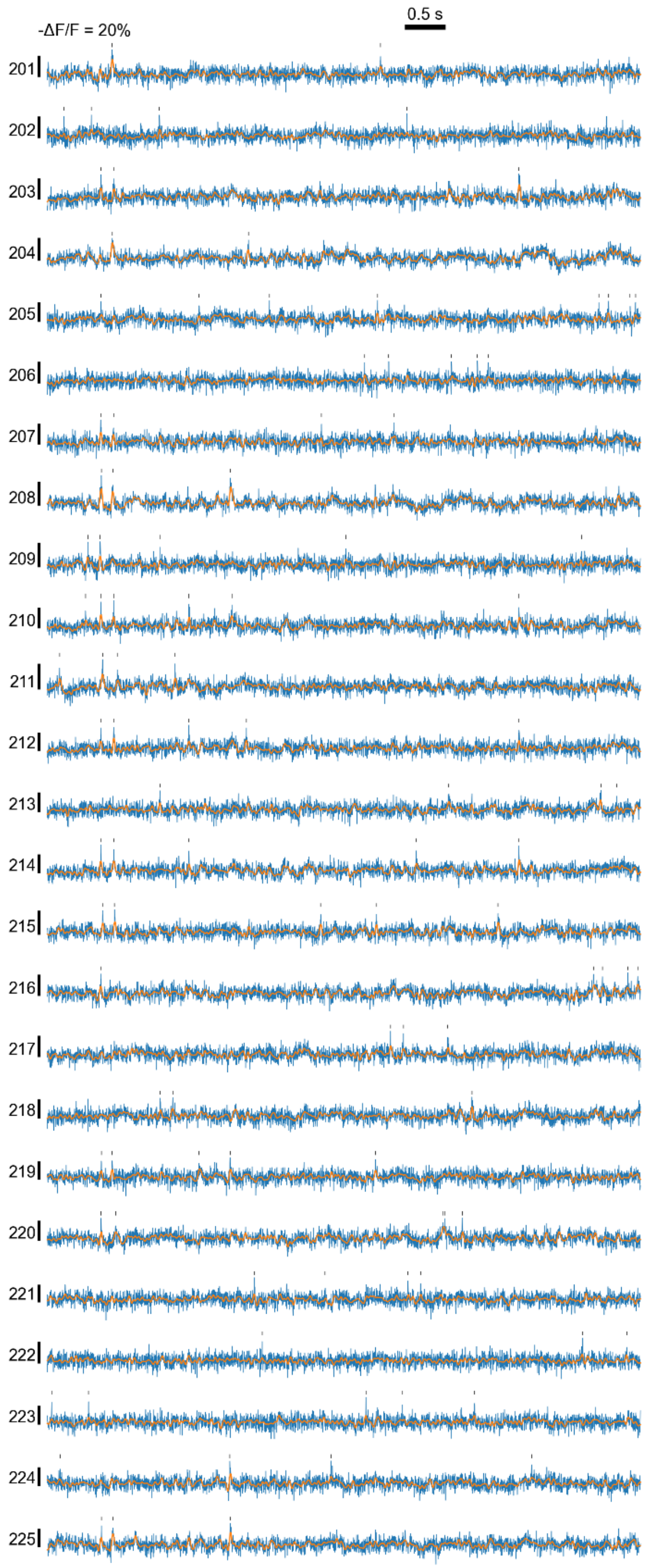

Supplementary Figure 3. Representative -ΔF/F voltage traces (in blue) of all 225 neurons in Fig. 3. Black ticks: suprathreshold spikes. Orange traces: extracted subthreshold -ΔF/F.

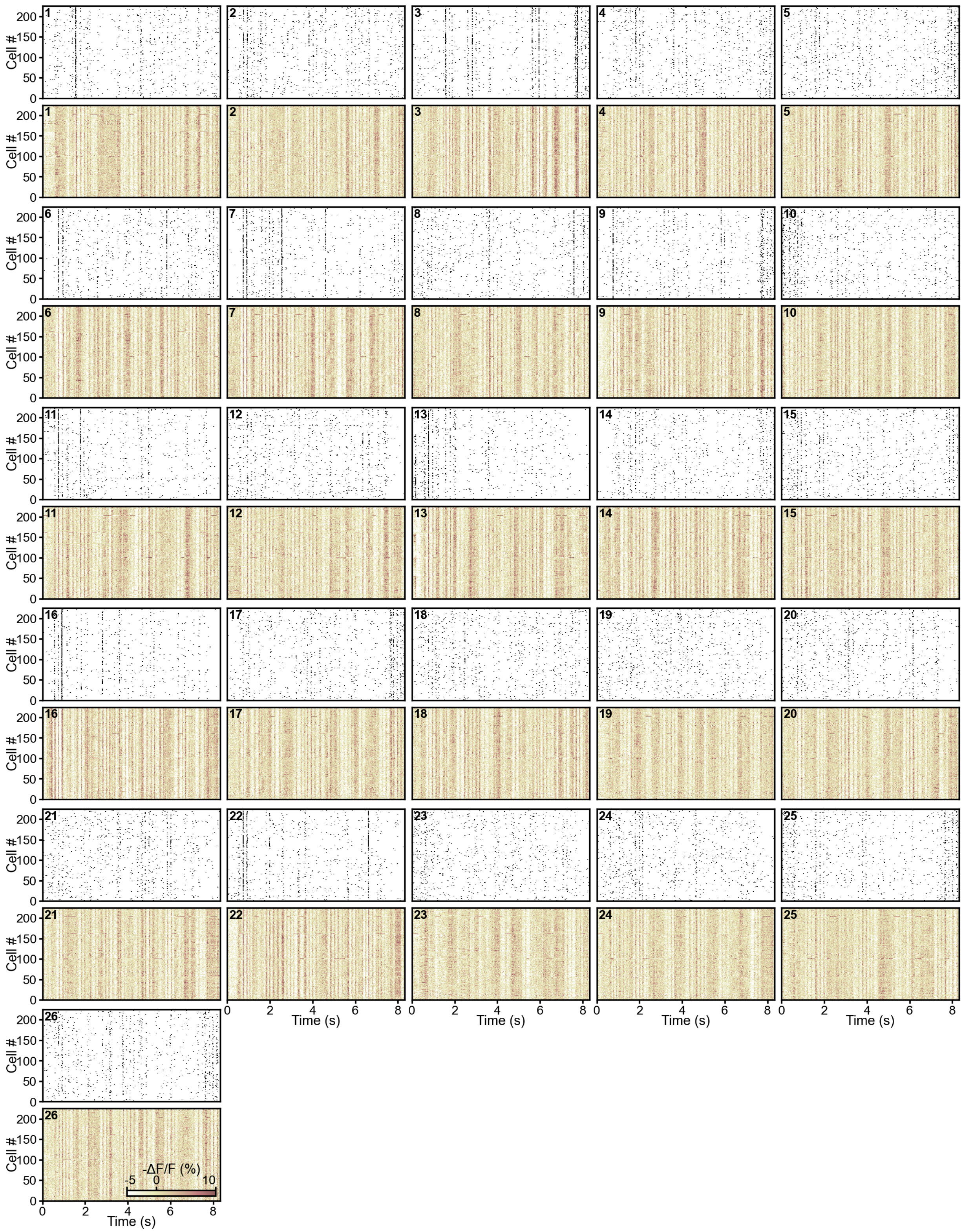

Supplementary Figure 4. Raster plots of (top) spikes and (bottom) subthreshold -ΔF/F traces from 26 8.32-s trials of 225 neurons in Fig. 3.

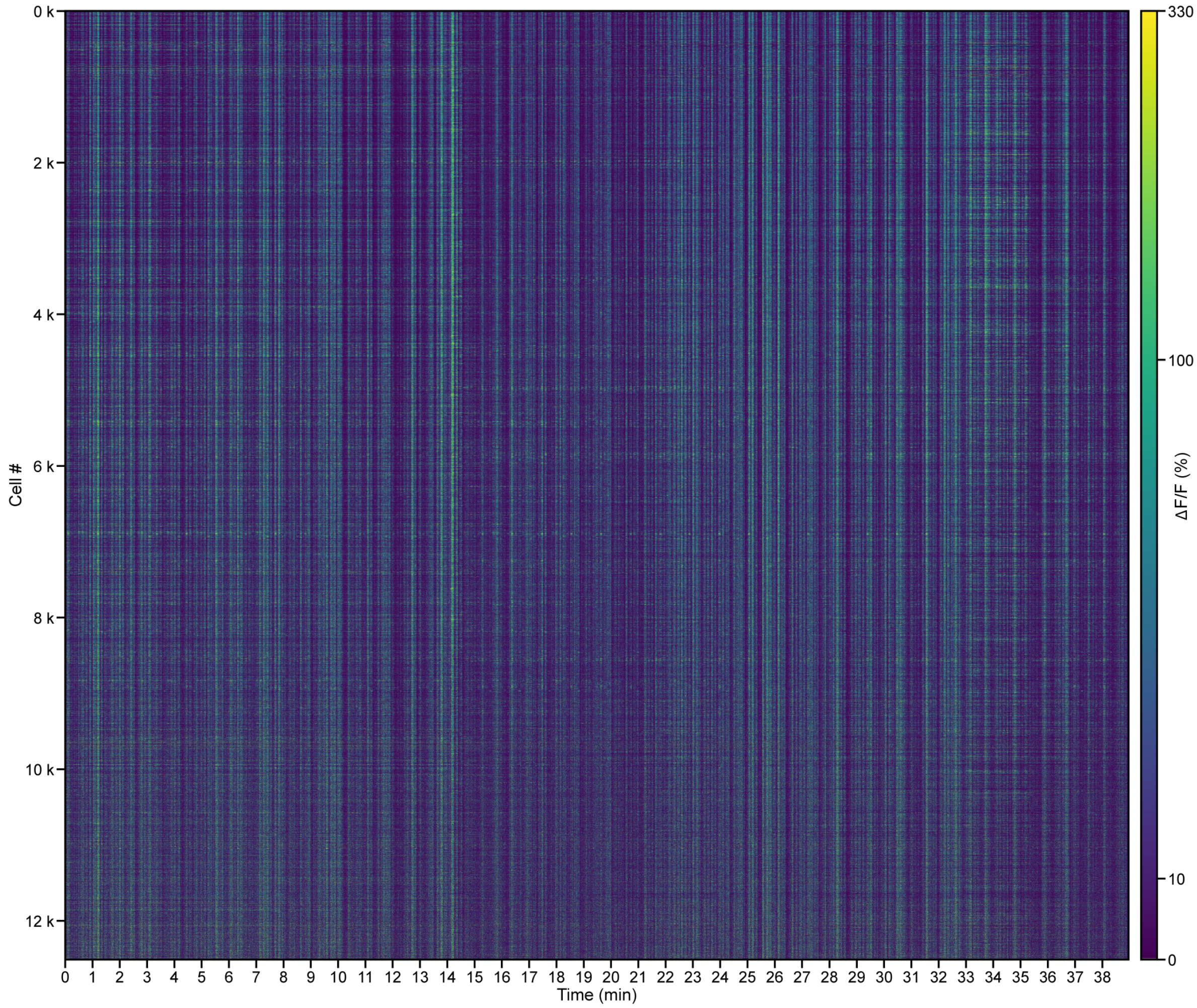

Supplementary Figure 5. Simultaneously recorded ΔF/F calcium traces from 12,511 neurons during 36 64-s trials. Same data as **Fig. 4**.

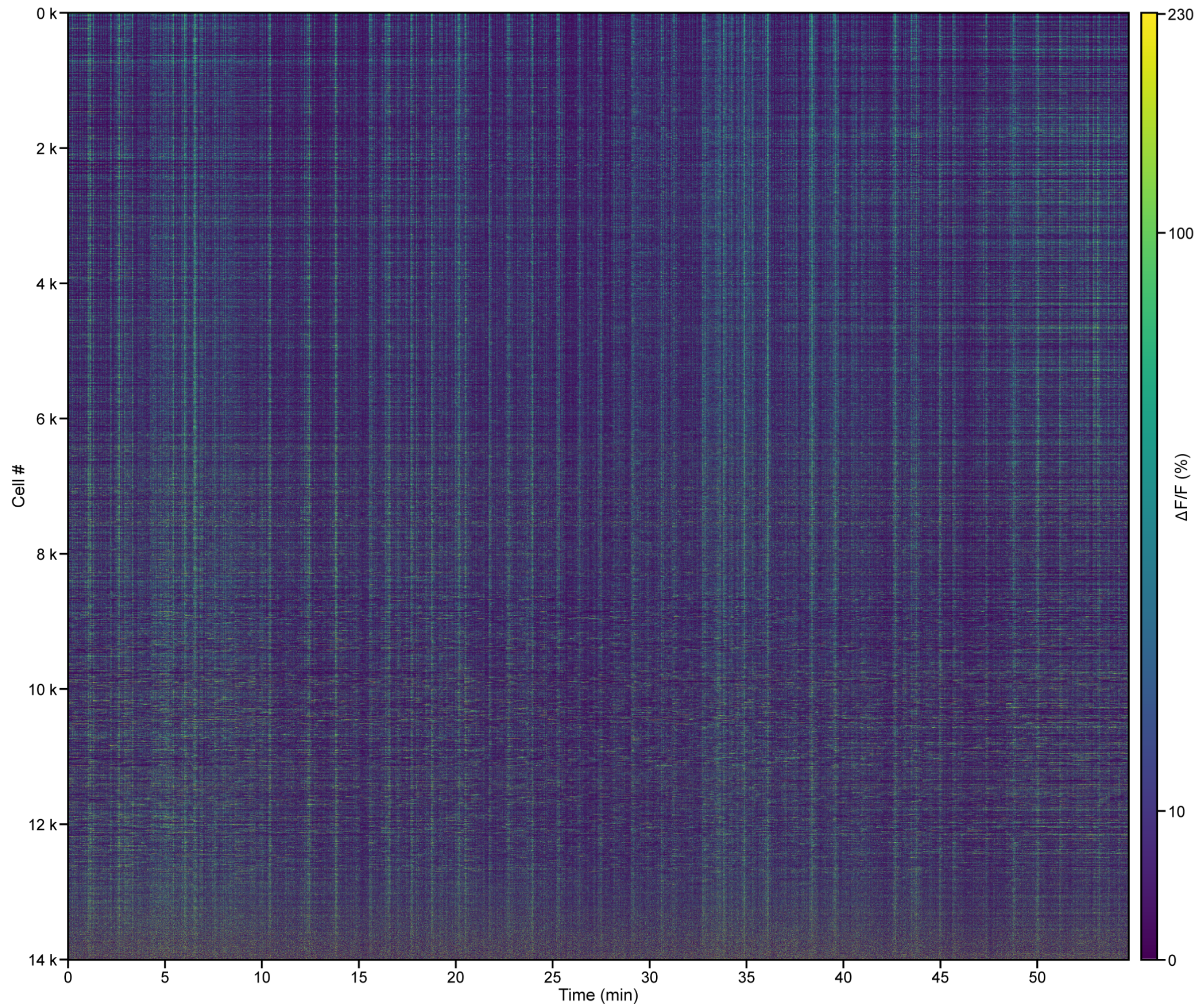

Supplementary Figure 6. Simultaneously recorded ΔF/F calcium traces from 14,005 neurons in during 51 trials. Same data as **Fig. 5**.

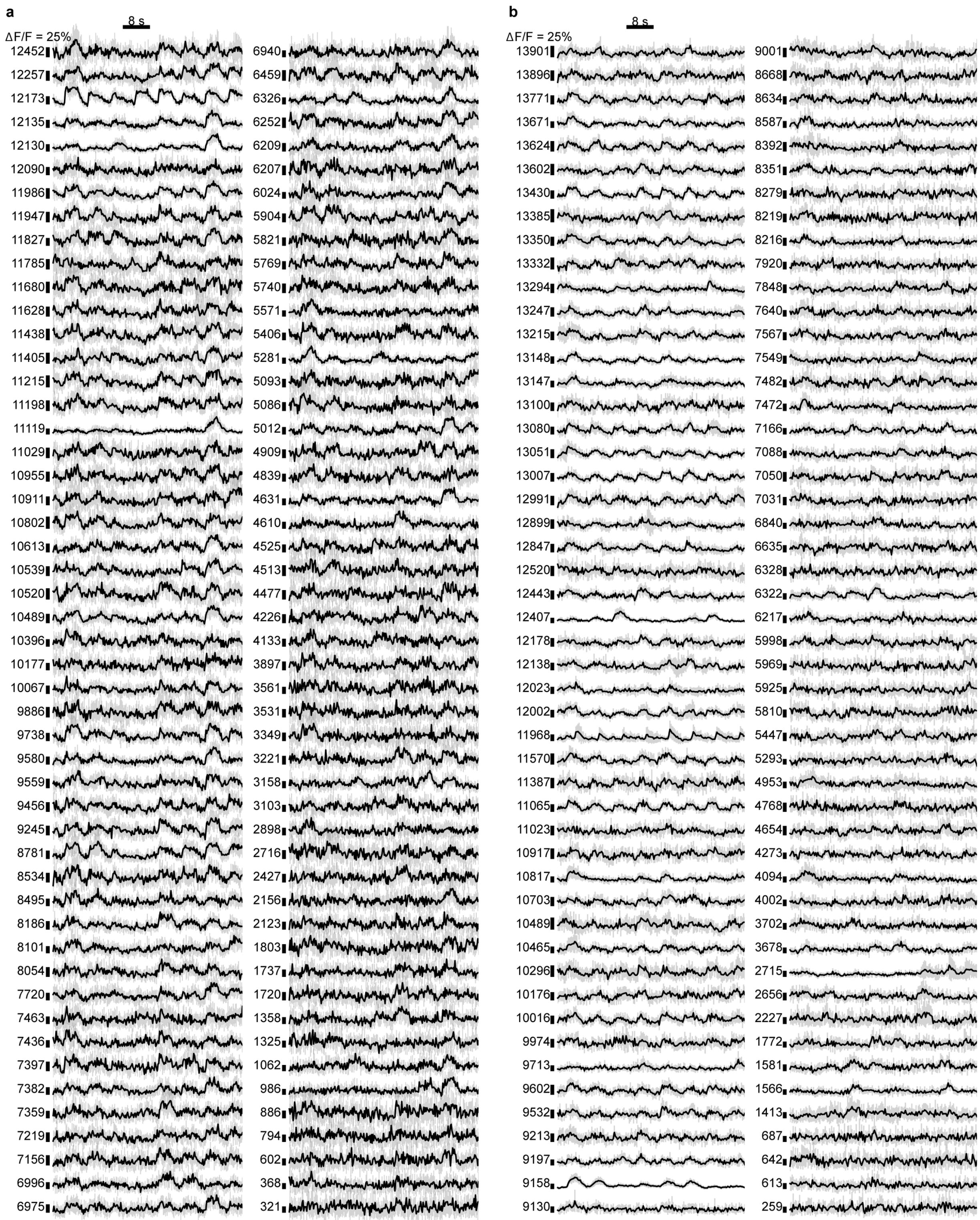

Supplementary Figure 7. Example 10-trial-averaged ΔF/F calcium traces from calcium imaging datasets. Example neurons were randomly selected from the visually evoked neurons across all depth ranges within the imaging volume of **Fig. 4** and **Fig. 5**, which was achieved by sampling neurons from a discrete uniform distribution encompassing all visually evoked neurons (implemented by the Python randint() function). Numbers: Cell numbers for selected neurons. Black curves: 10-trial averaged ΔF/F calcium traces. Gray region: standard deviation.

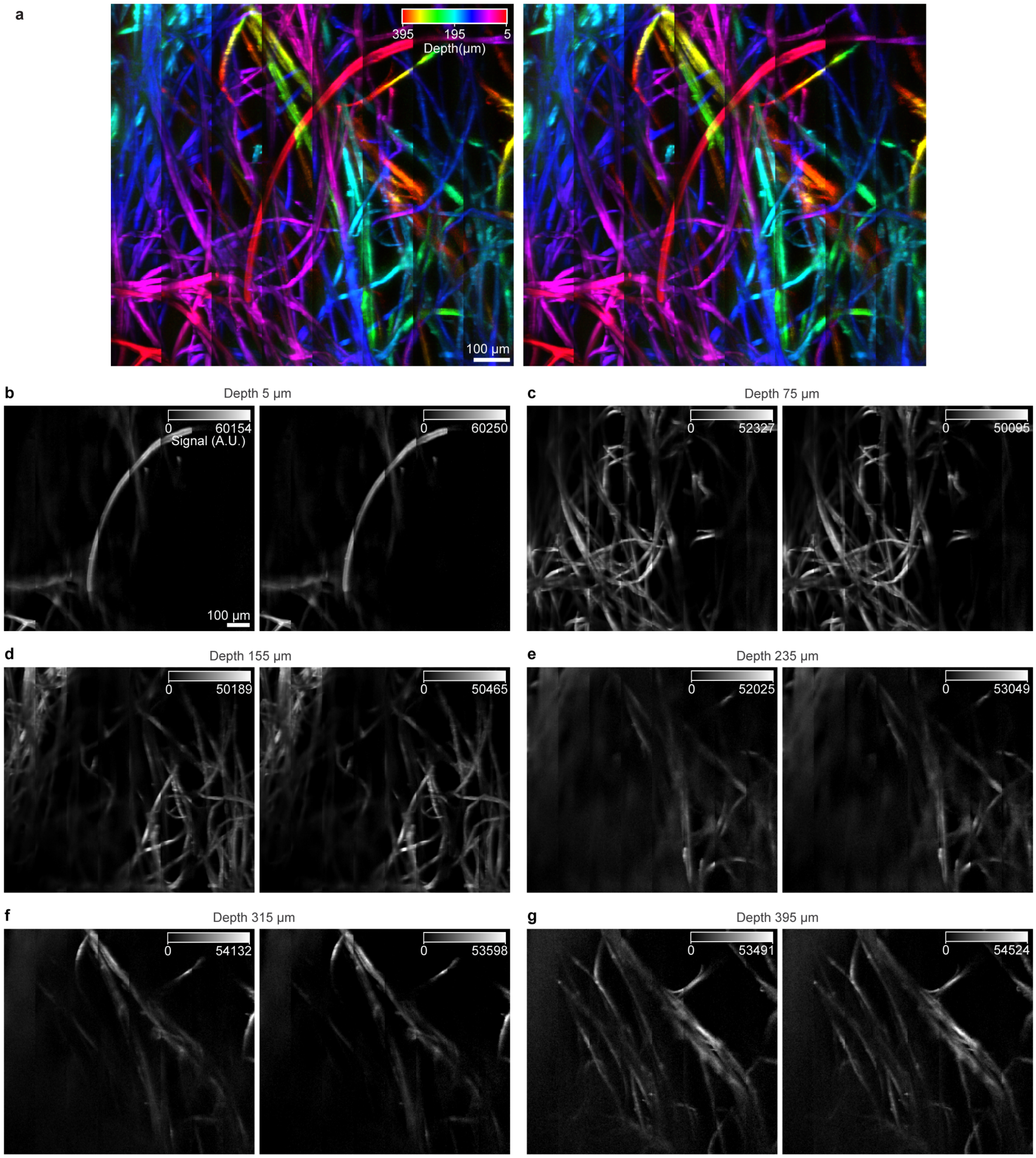

Supplementary Figure 8. FACED volumetric imaging accurately captures 3D structures in a 1,111 µm × 1,000 µm × 400 µm volume at 259.7 ms/vol without in-frame motion artifacts. Comparison of ground-truth (left, acquired by keeping the objective lens stationary at each depth) and volumetric (right, acquired by continuous Z scanning) images of fluorescent fibers. (**a**) Maximum intensity projection of 3D fiber structures (color-coded by depth) across the entire imaging volume for both ground-truth (left) and volumetric (right) datasets. (**b-g**) Representative ground-truth (left) and volumetric (right) images at depths of 5 µm, 75 µm, 155 µm, 235 µm, 315 µm, and 395 µm. Voxel size: 1.4 µm × 2 µm × 10 µm. 1035 nm excitation at 21 mW post 16× 0.8-NA objective.

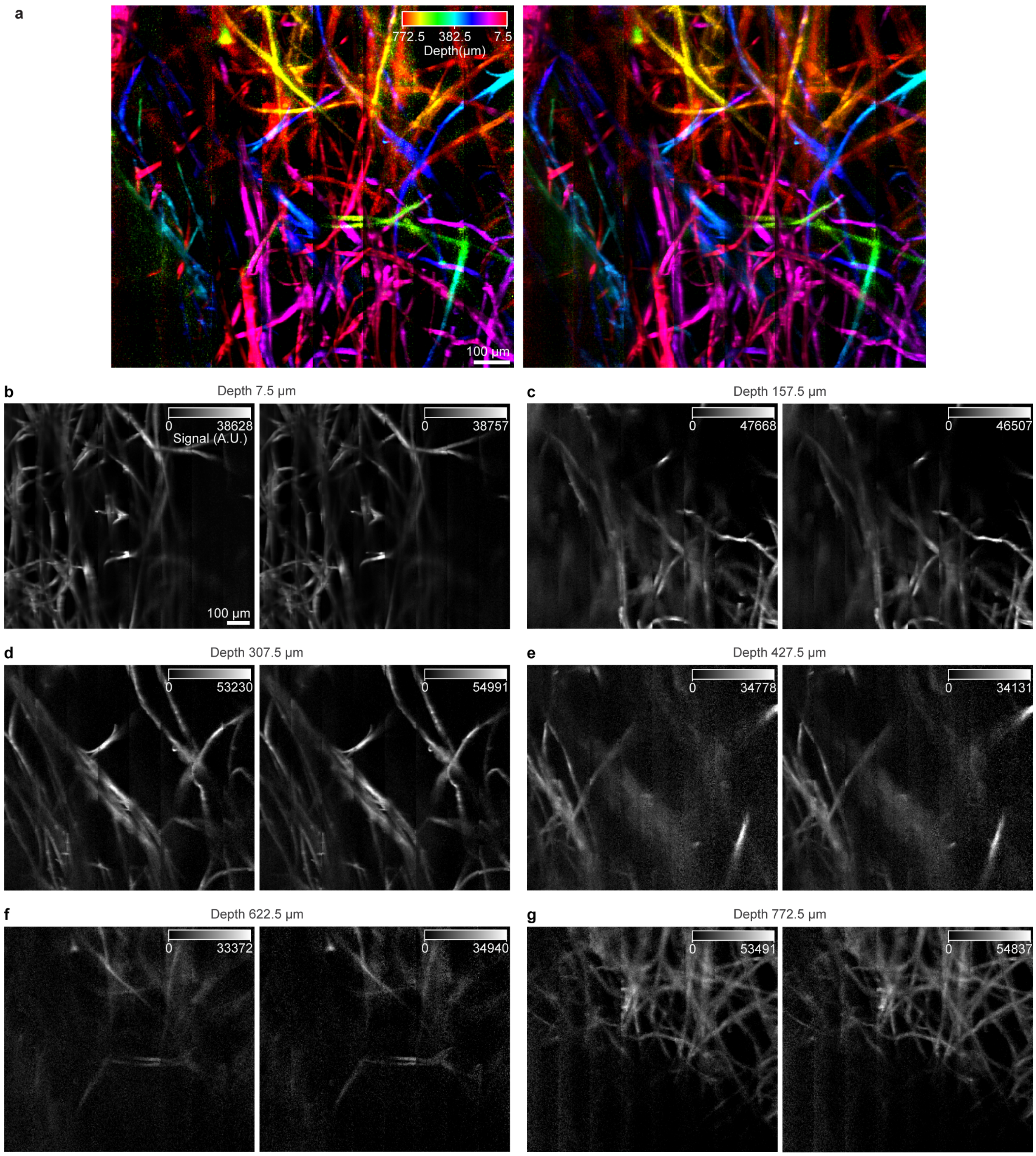

Supplementary Figure 9. FACED volumetric imaging accurately captures 3D structures in a 1,111 µm × 1,000 µm × 780 µm volume at 365.7 ms/vol without in-frame motion artifacts. Comparison of ground-truth (left, acquired by keeping the objective lens stationary at each depth.) and volumetric (right, acquired by continuous Z scanning) images of fluorescent fibers. (**a**) Maximum intensity projection of 3D fiber structures (color-coded by depth) across the entire imaging volume for both ground-truth (left) and volumetric (right) datasets. (**b-g**) Representative ground truth (left) and volumetric (right) images at depths of 7.5 µm, 157.5 µm, 307.5 µm, 427.5 µm, 622.5 µm, and 772.5 µm. Voxel size: 1.4 µm × 2 µm × 15 µm. 1035 nm excitation at 21 mW post 16× 0.8-NA objective.

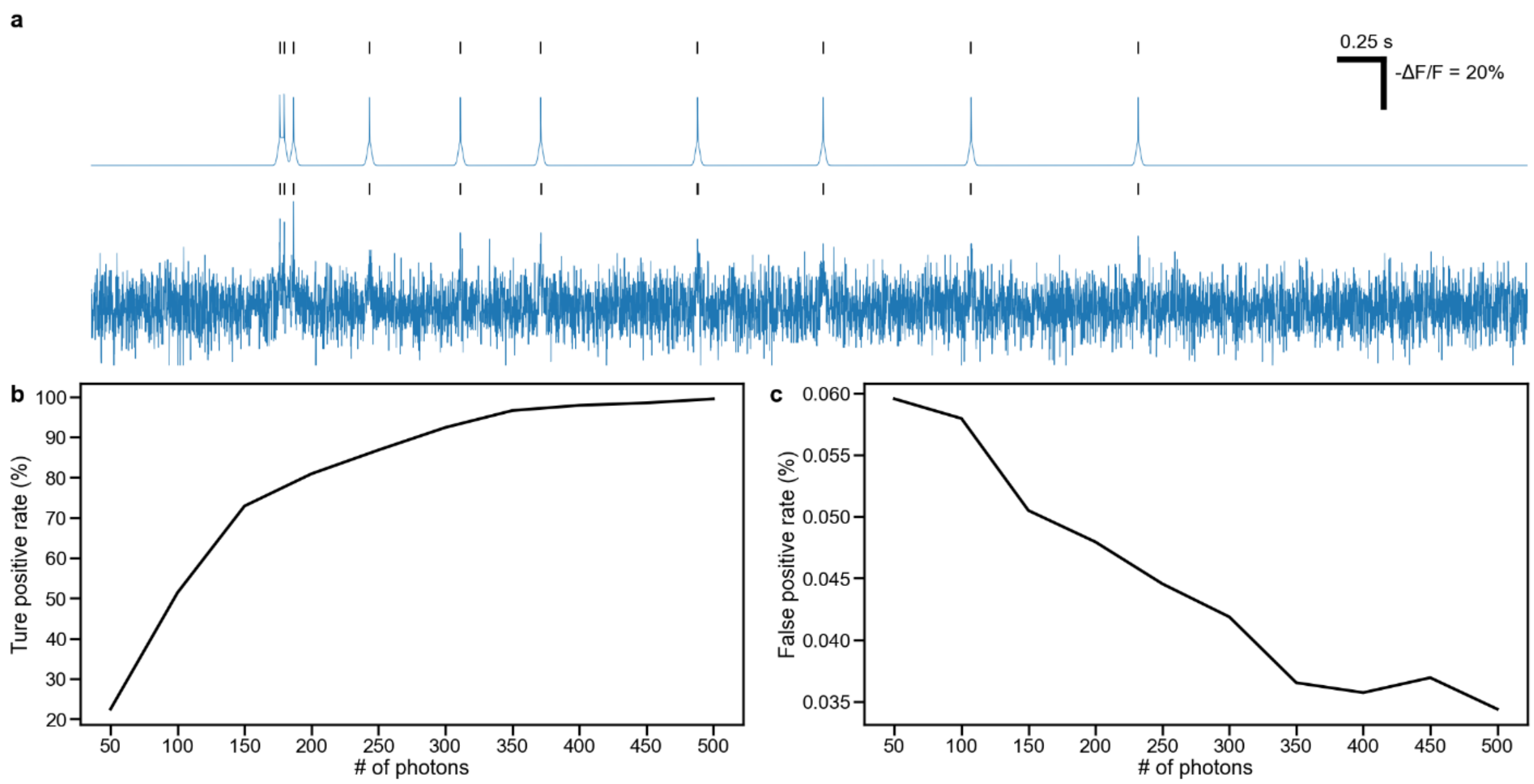

Supplementary Fig. 10. Validation of the spike detection method on simulated voltage traces. (**a**) Top trace: an example ground-truth voltage trace. Top black ticks: ground-truth spikes. Bottom trace: corresponding noise-added voltage trace with a SNR from 150 photons/frame. Bottom black ticks: spikes detected from noise-added voltage trace. (**b**) True positive rate of spike detection across different SNR conditions (SNR is quantified by number of photons per frame) (**c**) False positive rate of spike detection across different SNR conditions (SNR is quantified by number of photons per frame). For **b** and **c**, data point for each SNR condition is computed from 1000 simulated noisy traces with the number of spikes randomly selected (1 to 50) and randomly positioned within the trace duration (**Supplementary Codes**).

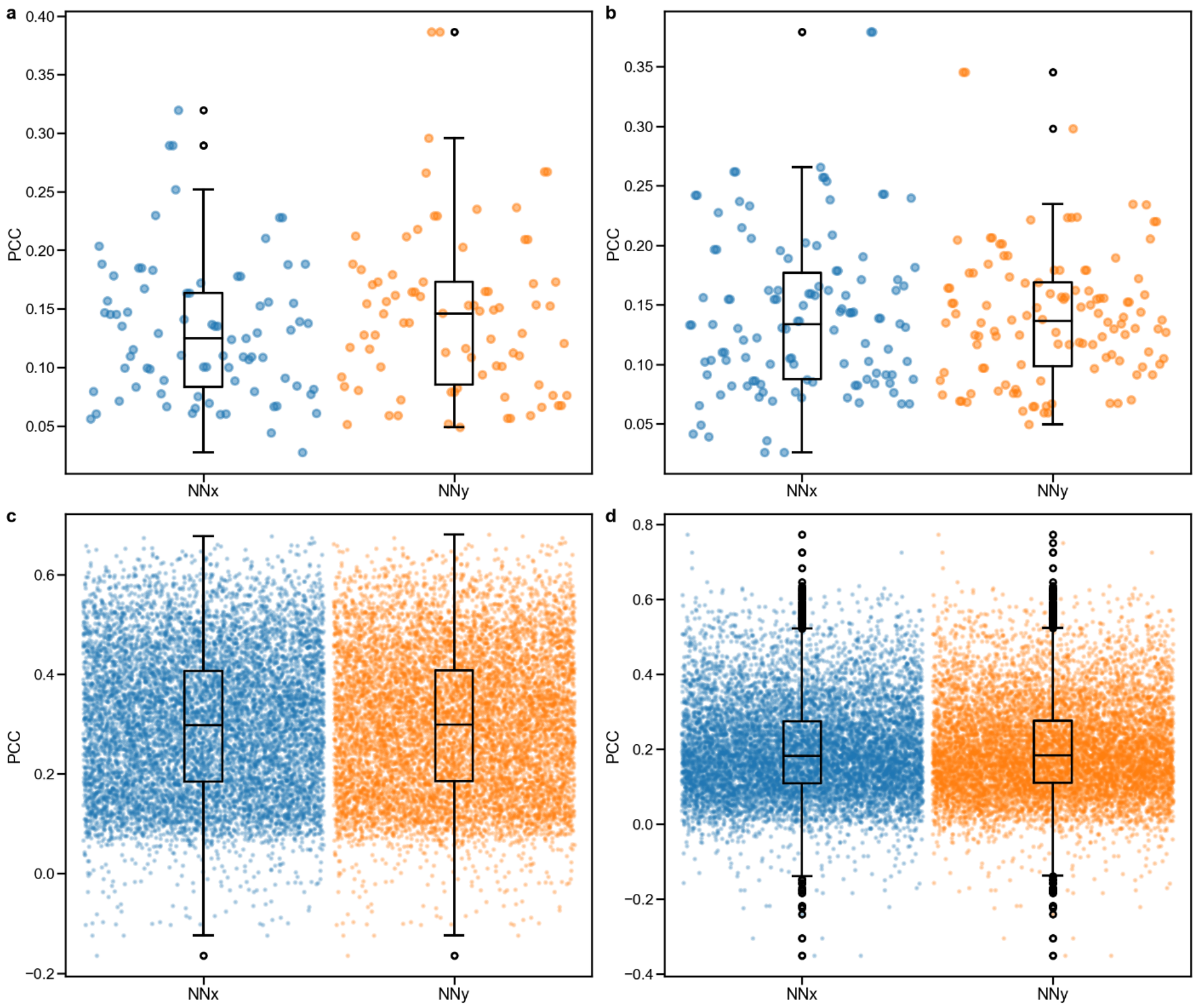

Supplementary Figure 11. Comparison of Pearson correlation coefficients (PCCs) between activity traces of neurons that are nearest neighbors along X axis (direction with potential contamination due to fluorescence lifetime) and Y axis (direction without the possibility of contamination). (**a,b**) Scatter and box-and-whisker plots of PCCs of voltage traces from **Fig. 2** and **Fig. 3**, respectively. (**c,d**) Scatter and box-and-whisker plots of PCCs of calcium traces from **Fig. 4** and **Fig. 5**, respectively. Blue dots and "NNx": PCC between two neurons that are nearest neighbors along X axis; Orange dots and "NNy": PCC between two neurons that are nearest neighbors along Y axis. Box-and-whisker plots: box represents interquartile range (IQR) extending from the first quartile to the third quartile; horizontal line indicates median; whisker extends to the most extreme data points within 1.5×IQR from box edge; black circles: outliers. One-sided t-tests comparing PCC(NNx) and PCC(NNy) yields p-values of 0.92, 0.42, 0.73, and 0.70 for the data in **a**, **b**, **c**, and **d**, respectively. Each scatter plot in **a**, **b**, **c**, and **d** contains data points from 81, 118, 9575, 9638 pairs of neurons, respectively.

Supplementary Notes

1. Additional characterization of volumetric imaging via continual Z movement of microscope objective

For volumetric imaging, FACED 2.0 uses a fast piezo stage to continuously oscillate the 16× 0.8-NA objective axially. To confirm that the continual Z movements during image acquisition did not generate in-frame motion artifacts, we compared 3D image stacks of static fluorescent fiber structures acquired at different rates (**Supplementary Figs. 8,9**).

For ground-truth measurements, we stepped and stopped the objective piezo at a Z position and acquired 100 XY images of 1,110 µm × 1,000 µm FOVs at 5.3 ms/frame; we repeated this at 40 Z positions with 10 µm Z-step size (**Supplementary Fig. 8**) or at 52 Z positions with 15 µm Z-step size (**Supplementary Fig. 9**) to cover the same volumes as in **Fig. 4** and **Fig. 5**, respectively. The averages of the 100 XY images at each Z position are used as the ground-truth volumetric datasets (left panels, **Supplementary Figs. 8,9**).

We then imaged the same structures by continuously moving the objective in Z to achieve the same volumetric rates as in **Fig. 4** (259.7 ms/volume by scanning the objective piezo continuously at 1,887 µm /s) and **Fig. 5** (365.7 ms/volume by scanning the objective piezo continuously at 2,830 µm/s). We acquired 100 volumes and compared the volume-averaged XY images at the same depths (right panels, **Supplementary Figs. 8,9**) with those from the ground-truth datasets.

Visual inspection indicates that the image stacks acquired by these two methods are highly similar. To quantify the similarity between the two stacks, we calculated the normalized cross-correlation coefficients (ρ_{XY}), which measures how well the images match, with higher values indicating greater similarity:

$$\rho_{XY} = \frac{\frac{1}{NML}\sum_{i=1}^{N}\sum_{j=1}^{M}\sum_{k=1}^{L}(X_{ijk} - \mu_X)(Y_{ijk} - \mu_Y)}{\sigma_X \sigma_Y}$$

Here, X_{ijk} and Y_{ijk} are the pixel values at position (i, j, k) in the volumetric (X) and ground-truth stacks (Y), respectively. N, M, and L represent the number of pixels along the X, Y, and Z axes. μ_X and μ_Y are the means, and σ_X and σ_Y are the standard deviations of pixel values within the entire image stacks:

$$\mu_X = \frac{1}{NML}\sum_{i=1}^{N}\sum_{j=1}^{M}\sum_{k=1}^{L}(X_{ijk}), \mu_Y = \frac{1}{NML}\sum_{i=1}^{N}\sum_{j=1}^{M}\sum_{k=1}^{L}(Y_{ijk})$$

$$\sigma_X = \sqrt{\frac{1}{NML}\sum_{i=1}^{N}\sum_{j=1}^{M}\sum_{k=1}^{L}(X_{ijk} - \mu_X)^2}, \sigma_Y = \sqrt{\frac{1}{NML}\sum_{i=1}^{N}\sum_{j=1}^{M}\sum_{k=1}^{L}(Y_{ijk} - \mu_Y)^2}$$

We found $\rho_{XY} = 98\%$ for data in **Supplementary Fig. 8** and $\rho_{XY} = 95\%$ for data in **Supplementary Fig. 9**, demonstrating a high level of agreement between the stack acquired through continuous Z scanning and the ground-truth stack acquired through step-and-stop Z scanning.

2. Evaluation of spike detection pipeline using simulated data

We further validated the accuracy of our spike detection pipeline using simulating voltage traces. The simulated traces contain supra- and subthreshold -ΔF/F activity similar to the *in vivo* data (at 1.25 ms time step size, **Supplementary Fig. 10**). The simulated suprathreshold spikes had 20% -ΔF/F and 2 ms FWHM. For simulated subthreshold activity, we set the mean peak fluctuation amplitude to be 8% ΔF/F. Poisson noise, corresponding to the photon count per frame, was then added to the ground-truth traces to approximate photon noise (**Supplementary Fig. 10a**).

We then processed these simulated traces with our spike detection pipeline and calculated the true positive rates (the percentage of ground-truth spikes that were detected by our pipeline) and the false positive rates (the number of incorrectly detected spikes divided by the total number of time points of the recording) at different photon counts (**Supplementary Fig. 10b,c**). As expected, higher photon counts are associated with more accurate spike detection. At the average photon counts for neurons in **Fig. 2** (316.2 photons per ROI, **Supplementary Table 5**), our method achieved >90% true positive rates and ~0.04% false positive rates (i.e., ~4 false spikes in 10,000 time points or a 12.5-s-long recording).

3. Voltage imaging at 800 Hz versus 400 Hz

We carried out additional analysis to determine whether ~400 Hz frame rate is sufficient for revealing most population voltage activity characteristics. We down-sampled the 800 Hz data in **Fig. 2** (by keeping alternate data points in time) to 400 Hz and then carried out the same analyses on correlated activity, timing and magnitude of visually evoked response, input-output function, and population orientation tuning properties (**Extended Data Fig. 8**).

Unsurprisingly, given the much slower variation of subthreshold activity with time, properties related to subthreshold voltage activity alone do not show perceivable differences between the original 800 Hz data and the down-sampled 400 Hz data (lower panels, **Extended Data Fig. 8a,d,g,j**). Measured spike rate is lower in the down-sampled 400-Hz data than in 800-Hz data (upper panels, **Extended Data Fig. 8d,j**), likely because the slower sampling rate misses APs. However, both datasets have similar timing and temporal variation of firing rates (upper panels, **Extended Data Fig. 8a,d,g,j**).

Activity correlation across the population is insensitive to frame rate except for neuron pairs with highest correlated spiking activity (**Extended Data Fig. 8b,h**). Distributions of the exponent of the input-output function also have very similar median values (2.17 vs 2.23), with the main difference being that 800 Hz sampling revealed more highly nonlinear neurons (e.g., those with exponents larger than 3) than the down-sampled 400 Hz data (**Extended Data Fig. 8c,i**). These differences are likely caused by 400 Hz data tending to miss more APs during burst firing.

Although the numbers of neurons that were found to have visually evoked AP firing are the same for the 800 Hz and 400 Hz data (88 out of the total 182 neurons), the number of neurons with suprathreshold orientation selectivity is higher for the 800 Hz data (43 neuros at 800 Hz vs. 30 neurons at 400 Hz). However, OSI distributions are again highly similar between the two datasets (median: 0.66 vs. 0.63; **Extended Data Fig. 8e,f,k,l**).

The above analysis indicates that imaging JEDI-2P-Kv-expressing neurons at ~400 Hz can inform on neuronal activity with similar accuracy as imaging at 800 Hz for most application scenarios. 400 Hz data become less accurate only when high-fidelity recording of high-frequency firing is required.

4. Discussion on potential contamination due to fluorescence lifetime in activity imaging

In the current FACED implementation, consecutive FACED foci are delayed by 1 ns. For fluorophores with lifetimes substantially longer than 1 ns, fluorophores excited by one FACED focus may emit their fluorescence photons during the data acquisition periods of subsequent pixels. Because most fluorescent proteins have lifetimes around 3 ns[1], this would lead to a practical decrease of the lateral resolution along the FACED linescan direction, as can be seen in structural images of dendritic spines (**Fig. 1d, Extended Data Fig. 4a**). However, as detailed below, this lifetime effect minimally affects population activity imaging of neuronal cell bodies (e.g., data in **Fig. 2,3**).

FACED is distinct from other 2PFM techniques that utilize temporal multiplexing for fast scanning. Those techniques utilize much longer delays (for example, 3 ns delay[2], 6.7 ns delay[3], and 8 ns delay[4]), because consecutive laser pulses and thus pixels sample distinct structures tens of microns apart along either lateral or axial direction. For such implementations, it is essential to have large temporal delay in order to avoid crosstalk of signals from distinct structures. In contrast, neighboring pulses in FACED linescans probe structures that are 0.8 µm (for voltage imaging) or 1.4 µm (for calcium imaging) apart along the X direction. Therefore, neighboring pixels mostly sample the cell body of the same neurons and contain similar activity information.

When analyzing somatic voltage and calcium activity, ROIs are selected to include the entire somata. The values of pixels inside each ROI are then averaged to obtain its activity trace, because these pixels contain the same information and pixel averaging gives rise to higher SNR. At our pixel sizes, each cell body is represented by on average 244 pixels for voltage imaging and 100 pixels for calcium imaging. With the FACED foci scanning from left to right, the only pixels that may be affected are those at the left edge of the ROI, with their signal contaminated by pixels to the left of the ROI. There are on average 17 pixels (for voltage imaging) or 9 pixels (for calcium imaging) at the left edge of the ROIs, corresponding to only 6.8% and 8.5% of total pixels for voltage and calcium imaging ROIs, respectively.

In the case of voltage imaging, due to soma-targeting of JEDI-2P-Kv, neuropil to the left of the ROIs had much lower brightness than the ROIs themselves. For calcium imaging, expression level in the neuropil was higher, but neuropil activity is still of much smaller magnitude than somatic activity[5]. Together, these factors further minimize contamination in the activity traces due to fluorescence lifetime.

That crosstalk due to fluorescence lifetime minimally contaminates the activity traces of individual neurons is supported by quantitative analysis: If there is substantial crosstalk, then two neurons that are neighbors along the FACED scan direction (X axis) would have more correlated activity (because fluorescence from the neuron on the left would contaminate the signal of the neuron on the right) than two neurons that are neighbors along the Y axis. We calculated the Pearson correlation coefficients (PCC) between the activity traces of the nearest neighbors along X direction ("NNx", blue data points, **Supplementary Fig. 11**) and

---

[1] Mamontova, A. V. et al. Bright GFP with subnanosecond fluorescence lifetime. Sci. Rep. 8, 13224 (2018).

[2] Cheng, A., Gonçalves, J. T., Golshani, P., Arisaka, K. & Portera-Cailliau, C. Simultaneous two-photon calcium imaging at different depths with spatiotemporal multiplexing. Nat. Methods 8, 139–142 (2011).

[3] Demas, J. et al. High-speed, cortex-wide volumetric recording of neuroactivity at cellular resolution using light beads microscopy. Nat. Methods 18, 1103–1111 (2021).

[4] Platisa, J. et al. High-Speed Low-Light In Vivo Two-Photon Voltage Imaging of Large Neuronal Populations. bioRxiv 2021.12.07.471668 (2021) doi:10.1101/2021.12.07.471668.

[5] Pachitariu, M. et al. Suite2p: beyond 10,000 neurons with standard two-photon microscopy. bioRxiv (2017) doi:10.1101/061507.

along Y direction ("NNy", blue data points, **Supplementary Fig. 11**) for both voltage and calcium imaging data.

As shown in **Supplementary Fig. 11**, the PCCs of activity traces of the closest neighbors along X axis and along Y axis exhibited similar distributions. One-sided t-tests on whether the PCC distribution along X is greater than the PCC distribution along Y have p-values of 0.95 (**Supplementary Fig. 11a**), 0.51 (**Supplementary Fig. 11b**), 0.73 (**Supplementary Fig. 11c**), and 0.70 (**Supplementary Fig. 11d**), indicating that there is no statistically significant difference between these distributions. Therefore, activity traces of neighboring neurons along X do not exhibit a higher correlation than those along Y, indicating that activity imaging does not suffer from contamination due to fluorescence lifetime.

5. FACED 2.0 versus FACED 1.0

FACED 2.0 substantially enhances the imaging capabilities of FACED 2PFM by offering larger imaging FOVs and longer continuous imaging durations than FACED 1.0, in addition to having volumetric imaging capability. These improvements, achieved via both hardware and software optimization, allow for selecting imaging area/volume/throughput based on the temporal dynamics of biological events, enabling the study of larger neuronal populations over wider spatial domains and longer timescales at higher throughput than FACED 1.0.

In FACED 1.0, the FACED module was configured to generate linescans containing 80 pixels at a 2 ns inter-pixel delay, with its imaging FOV restricted to 50 µm × 250 µm (25× 1.05 NA objective). In FACED 2.0, the FACED module is configured to generate linescans containing 100 pixels at a 1 ns inter-pixel delay, with a single FACED FOVs reaching 80 µm × 585 µm (25× 1.05 NA objective) or 140 µm × 1035 µm (16× 0.8 NA objective).

A complete rewrite of the microscope control software for FACED 2.0 allows us to increase Y galvo scanning to 833 Hz (1.67 kHz frame rate) over 585 µm for 25× 1.05-NA objective and 1,035 µm for 16× 0.8 NA objective, a substantial improvement over from FACED 1.0 with 500 Hz Y galvo scanning over 250 µm for 25× 1.05-NA objective. Adding an additional X galvo that scans along the FACED linescan direction in FACED 2.0 allows tiling FACED FOVs to further enlarge the area that can be scanned at high speed. For example, using the 16× 0.8 NA objective, an area of 1111 µm × 1000 µm can be scanned at 192 Hz.

The control software also synchronizes 2D FACED imaging with continuous axial scanning of the microscope objective, enabling volumetric imaging. The large FOV and high frame rate of FACED 2.0 enable calcium activity of >10,000 neurons to be collected over large volumes at true cellular resolution (e.g., 1111 µm × 1000 µm × 780 µm at 365.7 ms/volume).

FACED 1.0 utilized a PXIe-5160 digitizer (National Instruments), which limits the maximal continuous imaging duration to 6 s. FACED 2.0 uses a high-speed digitizer with onboard FPGA (ADQ7-DC, Teledyne SP Devices). Together with a custom multi-thread data acquisition program that optimizes data transfer and storage, it enables continuous data streaming, limited in its duration only by disk storage size.

Supplementary Table 1. Comparison of 2PFM techniques

Technique	Pixels/voxels per second	Lateral resolution: X × Y	Axial (Z) resolution	Max depth in mouse brain in vivo	2D imaging performance: FOV @ frame rate (pixel size)	3D imaging performance: Volume @ volume rate (voxel size)	Notes
FACED 2.0	1.0×10^8 [a]	0.83 µm × 0.53 µm (1.05-NA objective)<br><br>1.27 µm × 0.76 µm (0.8-NA objective)	2.0 µm (1.05-NA objective)<br><br>4.8 µm (0.8-NA objective)	757 µm	80 µm × 585 µm @ 1 kHz (0.8 µm × 0.65 µm)<br>80 µm × 585 µm @ 1.67 kHz (0.8 µm × 1.17 µm)<br><br>140 µm × 1035 µm @ 1 kHz (1.4 µm × 1.15 µm)<br><br>80 µm × 585 µm @ 1.67 kHz (0.8 µm × 2.07 µm)<br><br>160 µm × 400 µm @ 800 Hz (0.8 µm × 0.8 µm)<br><br>320 µm × 400 µm @ 385 Hz (0.8 µm × 0.8 µm)<br><br>1111 µm × 1000 µm @ 192 Hz (1.4 µm × 2 µm)	1111 µm × 1000 µm × 780 µm @ 2.7 Hz (1.4 µm × 2 µm × 15 µm)<br><br>1111 µm × 1000 µm × 400 µm @ 3.8 Hz (1.4 µm × 2 µm × 10 µm)<br><br>480 µm × 585 µm × 360 µm @ 3.8 Hz (0.8 µm × 0.65 µm × 12 µm)<br><br>555.2 µm × 800 µm × 360 µm @ 10.4 Hz (1.4 µm × 1.6 µm × 15 µm)	Current manuscript
FACED 1.0[1]	0.8×10^8 [a]	0.82 µm × 0.35 µm	3.0 µm	345 µm	50 µm × 250 µm @ 1 kHz (0.6 µm × 0.3 µm)	N/A	
Multifoci excitation[2]	2.0×10^9	1.4 µm × 1.4 µm	9.6 µm	300 µm	95,000 µm² @ 1 kHz (1024 × 196 pixels)<br><br>248,000 µm² @ ≤400 Hz (1024 × 512 pixels)	N/A	Camera detection: limited depth in opaque brains

Bessel focus scanning[3,4]	7.9×10^6	0.3-0.7 µm	20-160 µm	650 µm	N/A	180 µm × 180 µm × 160 µm @ 30 Hz (0.35 µm × 0.35 µm × 160 µm)<br><br>270 µm × 270 µm × 100 µm @ 30 Hz (0.53 µm × 0.53 µm × 100 µm)<br><br>301 µm × 450 µm × 612 µm @ 3.2 Hz (0.4 µm × 0.4 µm × 100 µm) [b]<br><br>3020 µm × 1500 µm × 600 µm @ 1 Hz (2 µm × 2 µm × 100 µm) [b]	Extended-depth excitation: requires sparse labeling
Light beads microscopy[5]	1.41×10^8	1.2 µm × 1.9 µm	19-30 µm	600 µm	N/A	600 µm × 600 µm × 500 µm @ 9.6 Hz (1 µm × 1 µm × 16 µm) [b]<br><br>900 µm × 900 µm × 500 µm @ 38 Hz (3.1 µm × 3.1 µm × 16 µm) [b]<br><br>3000 µm × 5000 µm × 500 µm @ 4.7 Hz (4.2 µm × 5 µm × 16 µm) [b]<br><br>5400 µm × 6000 µm × 500 µm @ 2.2 Hz (4.2 µm × 5 µm × 16 µm) [b]	Resolution values from Extended Data Fig. 4i,j
Light sculpting based on temporal focusing[6]	2.3×10^6	5 µm × 5 µm	10 µm	500 µm	500 µm × 500 µm @ 158 Hz (4.2 µm × 4.2 µm)<br><br>500 µm × 500 µm @ 10 Hz Dual plane (4.2 µm × 4.2 µm)	500 µm × 500 µm × 500 µm @ 3 Hz (4.2 µm × 4.2 µm × 11.9 µm)<br><br>500 µm × 500 µm × 200 µm @ 5.7 Hz (4.2 µm × 4.2 µm × 9.5 µm)	
Hybrid Multiplexed Sculpted Light (HyMS) Microscopy[7]	1.2×10^7	5 µm × 5 µm	15 µm	700 µm	N/A	690 µm × 675 µm × 600 µm @ 16.7 Hz (5 µm × 5 µm × 15 µm)<br><br>1000 µm × 1000 µm × 600 µm @ 5.1 Hz (5 µm × 5 µm × 15 µm)<br><br>765 µm × 665 µm × 640 µm @ 4.3 Hz (5 µm × 5 µm × 13.3 µm)	Data reported are for 2PFM modality
Scan multiplier	6.6×10^7	2.2 µm × 1.9 µm	8.2 µm	540 µm	200×200 µm @ 1kHz (0.78 µm × 0.78 µm)	N/A	

unit (SMU)[8,9]							
Optical gearbox[10]	7.2×10^7	0.49 µm × 0.48 µm	2.60 µm	460 µm	700 µm ×560 µm @ 50 Hz (0.52 µm × 0.52 µm)<br><br>344 µm ×274 µm @ 200 Hz (0.52 µm × 0.51 µm)<br><br>350 µm × 44 µm @ 1 kHz (0.58 µm × 0.59 µm)<br><br>155 µm × 124 µm @ 200 Hz (0.23 µm ×0.23 µm)<br><br>251 µm × 454 µm @ 200 Hz (0.38 µm × 0.43 µm)<br><br>196 µm × 196 µm @ 200 Hz (0.29 µm × 0.38 µm)	2 × 155 µm ×124 µm × 40 µm @ 10 Hz (0.23 µm × 0.23 µm × 4 µm)	Image FOV and pixel sizes are estimated from figures shared data

[a] Can be increased by simply increasing laser repetition rate; See Ref. 1.
[b] Applied in a mesoscope system.

[1] Wu, J. et al. Kilohertz two-photon fluorescence microscopy imaging of neural activity in vivo. Nat Methods 17, 287–290 (2020).
[2] Zhang, T. et al. Kilohertz two-photon brain imaging in awake mice. Nat Methods 16, 1119–1122 (2019).
[3] Lu, R. et al. Video-rate volumetric functional imaging of the brain at synaptic resolution. Nat Neurosci 20, 620–628 (2017).
[4] Lu, R. et al. Rapid mesoscale volumetric imaging of neural activity with synaptic resolution. Nat Methods 17, 291–294 (2020).
[5] Demas, J. et al. High-speed, cortex-wide volumetric recording of neuroactivity at cellular resolution using light beads microscopy. Nat Methods 18, 1103–1111 (2021).
[6] Prevedel, R. et al. Fast volumetric calcium imaging across multiple cortical layers using sculpted light. Nat Methods 13, 1021–1028 (2016).
[7] Weisenburger, S. et al. Volumetric Ca2+ Imaging in the Mouse Brain Using Hybrid Multiplexed Sculpted Light Microscopy. Cell 177, 1050-1066.e14 (2019).
[8] Xiao, S., Davison, I. & Mertz, J. Scan multiplier unit for ultrafast laser scanning beyond the inertia limit. Optica 8, 1403 (2021).
[9] Xiao, S., Giblin, J. T., Boas, D. A. & Mertz, J. High-throughput deep tissue two-photon microscopy at kilohertz frame rates. Optica 10, 763 (2023).
[10] Lin, J., Cheng, Z., Yang, G. & Cui, M. Optical gearbox enabled versatile multiscale high-throughput multiphoton functional imaging. Nat Commun 13, (2022).

Supplementary Table 2: Parameters for 2D imaging with FACED 2.0

Figure	Sample	FOV dimension (X µm × Y µm)	Pixel size (X µm × Y µm)	Rescaled pixel size for display (µm)	Frame rate (ms/frame)	Post-objective power @ wavelength	Imaging Depth (µm)	Microscope objective	Filter*	# of frame average for display
Fig. 1d	Thy1-GFP line M mouse	46 x 64 (Cropped from a larger image)	0.8 × 0.2	0.2	9.3	150 mW @ 1035 nm	36±2.5 (0.5 µm /step)	25× 1.05 NA	525/50 BP	400
Fig. 1e-g	Wild-type mouse injected with Rhodamine B	1111 × 1000	1.4 × 2.0	1.4	5.3	215 mW @ 1035 nm	50	16× 0.8 NA	680 SP	6000
Fig. 2	Wild-type mouse transfected with JEDI-2P-Kv voltage indicator	160 × 400	0.8 × 0.8	0.8	1.25	163 mW @ 1035 nm	132	25× 1.05 NA	680 SP	6600
Fig. 3	Wild-type mouse transfected with JEDI-2P-Kv voltage indicator	320 × 400	0.8 × 0.8	0.8	2.6	164 mW @ 1035 nm	140	25× 1.05 NA	680 SP	3200

*525/50 BP: band-pass filter with 525 nm center wavelength and 50 nm bandwidth; 680 SP: short-pass filter with 680 nm cutoff wavelength.

Supplementary Table 3: Parameters for 3D imaging with FACED 2.0

Figure	Sample	Volume dimension (X µm × Y µm × Z µm)	Voxel size (X µm × Y µm × Z µm)	Rescaled Voxel size for display (XY µm × Z µm)	Volume rate (ms/vol)	Post-Objective power @ wavelength	Imaging Depth (µm)	Microscope objective	Filter*	# of volumes average for display
Fig. 1b	Wild-type mouse injected with Rhodamine B	1111 × 1000 × 600	1.4 × 2.0 × 5.0	1.4 × 5.0	624	240 mW @ 1035 nm	25-625	16× 0.8 NA	680 SP	100
Fig. 4	a Slc17a7-IRES2-Cre × Ai162D mouse	1111 × 1000 × 400	1.4 × 2.0 × 10.0	1.4 × 10.0	259.7	102 mW @ 920 nm	0-400	16× 0.8 NA	525/50 BP	9000
Fig. 5	Wild-type mouse transfected with GCaMp6s	1111 × 1000 × 780	1.4 × 2.0 × 15.0	1.4 × 15.0	365.7	140 mW @ 920 nm	0-780	16× 0.8 NA	525/50 BP	8976
Extended Data Fig. 4a	Thy1-GFP line M mouse	92 × 128 (Cropped from larger raw image)	1.4 × 0.4	0.4	9.3	165 mW @ 1035 nm	29.5±5 (0.5 µm /step)	16× 0.8 NA	525/50 BP	400
Extended Data Fig. 4b	Thy1-GFP line M mouse	1111 × 1035	1.4 × 1.15	1.15	9.3	170 mW @ 1035 nm	50-800	16× 0.8 NA	525/50 BP	4000
Extended Data Fig. 5	Thy1-GFP line M mouse	640 × 585	0.8 × 0.65	0.65	9.3	140 mW @ 1035 nm	50-800	25× 1.05 NA	525/50 BP	4000
Extended Data Fig. 6	Wild-type mouse injected with Rhodamine B	1111 × 1000	1.4 × 2.0	1.4	5.3	208 mW @ 1035 nm	0-750	16× 0.8 NA	680 SP	6000

Extended Data Fig. 9	larval Tg[Elavl3:H2B-GCaMP6s] zebrafish	480 × 585 × 360	0.8 × 0.65 × 12.0	0.65 × 12.0	262.4	78 mW @ 920 nm	0-360	25×1.05 NA	525/50 BP	230
Extended Data Fig. 10	larval Tg[Elavl3:H2B-GCaMP6s] zebrafish	555.6 × 800 × 360	1.4 × 1.6 × 15.0	1.4 × 15.0	96	121 mW @ 920 nm	0-360	16× 0.8 NA	525/50 BP	500
Supp. Fig. 8	Fluorescent lens tissue fiber	1111 × 1000 × 400	1.4 × 2.0 × 10.0	1.4 × 10.0	259.7	21 mW @ 1035 nm	0-400	16× 0.8 NA	680 SP	100
Supp. Fig. 9	Fluorescent lens tissue fiber	1111 × 1000 × 780	1.4 × 2.0 × 15.0	1.4 × 15.0	365.7	21 mW @ 1035 nm	0-780	16× 0.8 NA	680 SP	100

*525/50 BP: band-pass filter with 525 nm center wavelength and 50 nm bandwidth; 680 SP: short-pass filter with 680 nm cutoff wavelength.

Supplementary Table 4: Brightness comparison for FACED 2.0 figures

Figure	Sample	Average of pixel value in the top 99% of image frame[1]	Frame/volume rate	Post-objective power @ wavelength	Imaging Depth (µm)	Microscope objective	Filter[2]	# of frame/volume average for figure display
Fig. 1b depth = 42.5 µm	Wild-type mouse injected with Rhodamine B	9821	624 ms/vol	240 mW @ 1035 nm	42.5	16× 0.8 NA	680 SP	100
Fig. 1b depth = 342.5 µm	Wild-type mouse injected with Rhodamine B	1078	624 ms/vol	240 mW @ 1035 nm	342.5	16× 0.8 NA	680 SP	100
Fig. 1b depth = 592.5 µm	Wild-type mouse injected with Rhodamine B	138	624 ms/vol	240 mW @ 1035 nm	592.5	16× 0.8 NA	680 SP	100
Fig. 1d	Thy1-GFP line M mouse	1372	9.3 ms/frame	150 mW @ 1035 nm	36±2.5 (0.5 µm /step)	25× 1.05 NA	525/50 BP	400
Fig. 1e	Wild-type mouse injected with Rhodamine B	7005	5.3 ms/frame	215 mW @ 1035 nm	50	16× 0.8 NA	680 SP	6000
Fig. 2	Wild-type mouse transfected with JEDI-2P-Kv	3766	1.25 ms/frame	163 mW @ 1035 nm	132	25× 1.05 NA	680 SP	6600
Fig. 3	Wild-type mouse transfected with JEDI-2P-Kv	3303	2.6 ms/frame	164 mW @ 1035 nm	140	25× 1.05 NA	680 SP	3200
Fig. 4b	Slc17a7-IRES2-Cre × Ai162D mouse	842	259.7 ms/vol	102 mW @ 920 nm	105	16× 0.8 NA	525/50 BP	9000
Fig. 4c	Slc17a7-IRES2-Cre × Ai162D mouse	497	259.7 ms/vol	102 mW @ 920 nm	185	16× 0.8 NA	525/50 BP	9000
Fig. 5b	Wild-type mouse transfected with GCaMP6s	1355	365.7 ms/vol	140 mW @ 920 nm	97.5	16× 0.8 NA	525/50 BP	8976
Fig. 5c	Wild-type mouse transfected with GCaMP6s	1277	365.7 ms/vol	140 mW @ 920 nm	142.5	16× 0.8 NA	525/50 BP	8976
Fig. 5d	Wild-type mouse transfected with GCaMP6s	474	365.7 ms/vol	140 mW @ 920 nm	412.5	16× 0.8 NA	525/50 BP	8976

Fig. 5e	Wild-type mouse transfected with GCaMP6s	195	365.7 ms/vol	140 mW @ 920 nm	517.5	16× 0.8 NA	525/50 BP	8976
Fig. 5f	Wild-type mouse transfected with GCaMP6s	34	365.7 ms/vol	140 mW @ 920 nm	697.5	16× 0.8 NA	525/50 BP	8976
Fig. 5i	Wild-type mouse transfected with GCaMP6s	29	365.7 ms/vol	140 mW @ 920 nm	742.5	16× 0.8 NA	525/50 BP	8976
Fig. 5k	Wild-type mouse transfected with GCaMP6s	27	365.7 ms/vol	140 mW @ 920 nm	757.5	16× 0.8 NA	525/50 BP	8976
Extended Data Fig. 4a	Thy1-GFP line M mouse	314	9.3 ms/frame	165 mW @ 1035 nm	29.5±5 (0.5 µm /step)	16× 0.8 NA	525/50 BP	400
Extended Data Fig. 4b depth = 50 µm	Thy1-GFP line M mouse	225	9.3 ms/frame	170 mW @ 1035 nm	50	16× 0.8 NA	525/50 BP	4000
Extended Data Fig. 4b depth = 400 µm	Thy1-GFP line M mouse	89	9.3 ms/frame	170 mW @ 1035 nm	400	16× 0.8 NA	525/50 BP	4000
Extended Data Fig. 4b depth = 750 µm	Thy1-GFP line M mouse	29	9.3 ms/frame	170 mW @ 1035 nm	750	16× 0.8 NA	525/50 BP	4000
Extended Data Fig. 5 depth = 50 µm	Thy1-GFP line M mouse	369	9.3 ms/frame	140 mW @ 1035 nm	50	25× 1.05 NA	525/50 BP	4000
Extended Data Fig. 5 depth = 400 µm	Thy1-GFP line M mouse	63	9.3 ms/frame	140 mW @ 1035 nm	400	25× 1.05 NA	525/50 BP	4000
Extended Data Fig. 5 depth = 750 µm	Thy1-GFP line M mouse	38	9.3 ms/frame	140 mW @ 1035 nm	750	25× 1.05 NA	525/50 BP	4000
Extended Data Fig. 6 depth = 50 µm	Wild-type mouse injected with Rhodamine B	2641	5.3 ms/frame	208 mW @ 1035 nm	50	16× 0.8 NA	680 SP	6000
Extended Data Fig. 6 depth = 400 µm	Wild-type mouse injected with Rhodamine B	321	5.3 ms/frame	208 mW @ 1035 nm	400	16× 0.8 NA	680 SP	6000
Extended Data Fig. 6 depth = 750 µm	Wild-type mouse injected with Rhodamine B	43	5.3 ms/frame	208 mW @ 1035 nm	750	16× 0.8 NA	680 SP	6000

Extended Data Fig. 9a Z = 330 µm	larval Tg[Elavl3:H2B-GCaMP6s] zebrafish	306	262.4 ms/vol	78 mW @ 920 nm	30	25×1.05 NA	525/50 BP	230
Extended Data Fig. 9a Z = 210 µm	larval Tg[Elavl3:H2B-GCaMP6s] zebrafish	250	262.4 ms/vol	78 mW @ 920 nm	150	25×1.05 NA	525/50 BP	230
Extended Data Fig. 9a Z = 90 µm	larval Tg[Elavl3:H2B-GCaMP6s] zebrafish	104	262.4 ms/vol	78 mW @ 920 nm	270	25×1.05 NA	525/50 BP	230
Extended Data Fig. 10 Z = 322.5 µm	larval Tg[Elavl3:H2B-GCaMP6s] zebrafish	132	96 ms/vol	121 mW @ 920 nm	37.5	16× 0.8 NA	525/50 BP	500
Extended Data Fig. 10 Z = 172.5 µm	larval Tg[Elavl3:H2B-GCaMP6s] zebrafish	182	96 ms/vol	121 mW @ 920 nm	187.5	16× 0.8 NA	525/50 BP	500
Extended Data Fig. 10 Z = 22.5 µm	larval Tg[Elavl3:H2B-GCaMP6s] zebrafish	56	96 ms/vol	121 mW @ 920 nm	337.5	16× 0.8 NA	525/50 BP	500
Supplementary Fig. 8c	Fluorescent lens tissue fiber	20961	259.7 ms/vol	21 mW @ 1035 nm	75	16× 0.8 NA	680 SP	100
Supplementary Fig. 8d	Fluorescent lens tissue fiber	20942	259.7 ms/vol	21 mW @ 1035 nm	155	16× 0.8 NA	680 SP	100
Supplementary Fig. 8g	Fluorescent lens tissue fiber	634	259.7 ms/vol	21 mW @ 1035 nm	395	16× 0.8 NA	680 SP	100
Supplementary Fig. 9b	Fluorescent lens tissue fiber	22198	365.7 ms/vol	21 mW @ 1035 nm	7.5	16× 0.8 NA	680 SP	100
Supplementary Fig. 9d	Fluorescent lens tissue fiber	1005	365.7 ms/vol	21 mW @ 1035 nm	307.5	16× 0.8 NA	680 SP	100
Supplementary Fig. 9g	Fluorescent lens tissue fiber	481	365.7 ms/vol	21 mW @ 1035 nm	772.5	16× 0.8 NA	680 SP	100

[1]On average, 1 photon is equivalent to 1300 pixel value.

[2]525/50 BP: band-pass filter with 525 nm center wavelength and 50 nm bandwidth; 680 SP: short-pass filter with 680 nm cutoff wavelength.

Supplementary Table 5: Estimated numbers of photons in activity measurement datasets

Figure number	Sample	75% percentile of photon counts in a ROI per frame	50% percentile of photon counts in a ROI per frame	25% percentile of photon counts in a ROI per frame	Average # of photon counts per ROI per frame	Standard deviation of photon counts per ROI per frame	Average # of photon counts per pixel within each ROI per frame
Fig. 2	Wild-type mouse transfected with JEDI-2P-Kv	414.1	298.7	203.0	316.2	138.6	1.4
Fig. 3		401.2	305.3	224.6	329.6	144.7	1.4
Fig. 4	Slc17a7-IRES2-Cre-D mouse	38.9	24.6	17.0	31.4	21.8	0.3
Fig. 5	Wild-type mouse transfected with GCaMp6s	48.8	29.1	19.0	38.8	29.2	0.3

Note: Number of photons were calculated from the baseline (F_0) trace of each ROI, using the conversion factor of a photon on average corresponding to 1300 in pixel value. The conversion factor was measured assuming Poisson statistics.

Supplementary Table 6: Estimated baseline noise level for activity measurement datasets

Figure number	Sample	Noise level for ROIs at 75% percentile of photon counts per frame	Noise level for ROIs at 50% percentile of photon counts per frame	Noise level for ROIs at 25% percentile of photon counts per frame	Noise level for average photon counts per frame
Fig. 2	Wild-type mouse transfected with JEDI-2P-Kv voltage indicator	4.9%	5.8%	7.1%	5.6%
Fig. 3		3.8%	4.6%	6.7%	5.5%
Fig. 4	Slc17a7-IRES2-Cre-D mouse	16.1%	20.2%	24.3%	17.8%
Fig. 5	Wild-type mouse transfected with GCaMp6s	14.4%	18.6%	23.0%	16.1%

Note: The baseline noise level is estimated from $\sqrt{N}$, where N represents the photon count from **Supplementary Table 5**. This noise level estimation is then normalized to baseline photon count N to represent the noise level in voltage traces.
