# Supplementary material for: FACED 2.0 enables large-scale voltage and calcium imaging in vivo
Source: Nat Methods. 2025 Dec 9;23(8):1611–21. doi: 10.1038/s41592-025-02925-7 (PMC13441978; doi:10.1038/s41592-025-02925-7)
Supplement: Supplementary file 2 [file 41592_2025_2925_MOESM2_ESM.pdf]

nature portfolio

Corresponding author(s): Na Ji

Last updated by author(s): Sep 28, 2025

Reporting Summary

Nature Portfolio wishes to improve the reproducibility of the work that we publish. This form provides structure for consistency and transparency in reporting. For further information on Nature Portfolio policies, see our Editorial Policies and the Editorial Policy Checklist.

Statistics

For all statistical analyses, confirm that the following items are present in the figure legend, table legend, main text, or Methods section.

n/a	Confirmed	
☐	☒	The exact sample size (*n*) for each experimental group/condition, given as a discrete number and unit of measurement
☐	☒	A statement on whether measurements were taken from distinct samples or whether the same sample was measured repeatedly
☐	☒	The statistical test(s) used AND whether they are one- or two-sided<br>*Only common tests should be described solely by name; describe more complex techniques in the Methods section.*
☒	☐	A description of all covariates tested
☒	☐	A description of any assumptions or corrections, such as tests of normality and adjustment for multiple comparisons
☐	☒	A full description of the statistical parameters including central tendency (e.g. means) or other basic estimates (e.g. regression coefficient) AND variation (e.g. standard deviation) or associated estimates of uncertainty (e.g. confidence intervals)
☐	☒	For null hypothesis testing, the test statistic (e.g. *F*, *t*, *r*) with confidence intervals, effect sizes, degrees of freedom and *P* value noted<br>*Give P values as exact values whenever suitable.*
☒	☐	For Bayesian analysis, information on the choice of priors and Markov chain Monte Carlo settings
☒	☐	For hierarchical and complex designs, identification of the appropriate level for tests and full reporting of outcomes
☒	☐	Estimates of effect sizes (e.g. Cohen's *d*, Pearson's *r*), indicating how they were calculated

Our web collection on statistics for biologists contains articles on many of the points above.

Software and code

Policy information about availability of computer code

Data collection	Data were collected using our customized program written with LabVIEW 2020, C++ 14, and Verilog 2005 for the imaging control and data acquisition of our ultrafast two-photon microscope. Acquisition codes can be found at https://github.com/JiLabUCBerkeley/FACED2PFM2p0-analysis
Data analysis	Imaging data were analyzed using Fiji (imageJ 2.3.0) and Python (3.11.2). Python modules: caiman 1.9.8, suite2p 0.13.1, and pykalman 0.9.5 are utilized for data processing. Analysis codes can be found at https://github.com/JiLabUCBerkeley/FACED2PFM2p0-analysis

For manuscripts utilizing custom algorithms or software that are central to the research but not yet described in published literature, software must be made available to editors and reviewers. We strongly encourage code deposition in a community repository (e.g. GitHub). See the Nature Portfolio guidelines for submitting code & software for further information.

Data

Policy information about availability of data

All manuscripts must include a data availability statement. This statement should provide the following information, where applicable:
- Accession codes, unique identifiers, or web links for publicly available datasets
- A description of any restrictions on data availability
- For clinical datasets or third party data, please ensure that the statement adheres to our policy

Raw structure images of Thy1-GFP mice can be found at https://figshare.com/s/fd97efe78295098ac37e. Raw blood flow and activity image data (> 1 TB) are available upon reasonable request to the correspondence author. Also see https://github.com/JiLabUCBerkeley/FACED2PFM2p0-analysis".

Human research participants

Policy information about studies involving human research participants and Sex and Gender in Research.

Reporting on sex and gender	Our study did not involve human subjects.
Population characteristics	Our study did not involve human subjects.
Recruitment	Our study did not involve human subjects.
Ethics oversight	Our study did not involve human subjects.

Note that full information on the approval of the study protocol must also be provided in the manuscript.

Field-specific reporting

Please select the one below that is the best fit for your research. If you are not sure, read the appropriate sections before making your selection.

☒ Life sciences ☐ Behavioural & social sciences ☐ Ecological, evolutionary & environmental sciences

For a reference copy of the document with all sections, see nature.com/documents/nr-reporting-summary-flat.pdf

Life sciences study design

All studies must disclose on these points even when the disclosure is negative.

Sample size	We imaged a total of 20 mice and 4 zebrafish larvae. No sample size calculation was performed. For demonstrating the capability of our microscope, the chosen sample numbers are sufficient.
Data exclusions	No data were excluded.
Replication	Similar results has been obtained over multiple experiments with distinct neurons recorded, with sample sizes reported in the paper.
Randomization	Randomized sampling were used to select representative activity traces to make figures.
Blinding	Blinding was not applicable, because the raw images by themselves indicated whether neurons were expressing activity indicators and whether blood flow were labeled. And analysis was only carried out for these neurons and blood vessels (neurons without expression and blood vessels without labeling did not provide data to be analyzed.)

Reporting for specific materials, systems and methods

We require information from authors about some types of materials, experimental systems and methods used in many studies. Here, indicate whether each material, system or method listed is relevant to your study. If you are not sure if a list item applies to your research, read the appropriate section before selecting a response.

Materials & experimental systems

n/a	Involved in the study
☒	☐ Antibodies
☒	☐ Eukaryotic cell lines
☒	☐ Palaeontology and archaeology
☐	☒ Animals and other organisms
☒	☐ Clinical data
☒	☐ Dual use research of concern

Methods

n/a	Involved in the study
☒	☐ ChIP-seq
☒	☐ Flow cytometry
☒	☐ MRI-based neuroimaging

Animals and other research organisms

Policy information about studies involving animals; ARRIVE guidelines recommended for reporting animal research, and Sex and Gender in Research

Laboratory animals	All experimental mice were purchased from Jackson Laboratories, including wildtype (C57BL/6J, stock no. 000664), Slc17a7-IRES2-Cre-D (stock no. 037512) × Ai162D (stock no. 031562) transgenic, and Thy1-GFP line M transgenic mice (stock no. 007788), and bred in house. All mice were >2-month old at the time of cranial window installation and housed under a reverse light cycle.<br>Nacre zebrafish larvae expressing Tg[Elavl3:H2B-GCaMP6s] bred by the Su Guo lab at UCSF were used.
Wild animals	Our study did not involve wild animals.
Reporting on sex	Both female and male were used.
Field-collected samples	Our study did not involve field-collected samples.
Ethics oversight	All animal experiments were conducted according to the National Institutes of Health guidelines for animal research. Procedures and protocols on mice and zebrafish were approved by the Institutional Animal Care and Use Committee at University of California, Berkeley.

Note that full information on the approval of the study protocol must also be provided in the manuscript.
