# Supplementary material for: FACED 2.0 enables large-scale voltage and calcium imaging in vivo
Source: Nat Methods. 2025 Dec 9;23(8):1611–21. doi: 10.1038/s41592-025-02925-7 (PMC13441978; doi:10.1038/s41592-025-02925-7)
Supplement: Supplementary file 3 [file 41592_2025_2925_MOESM3_ESM.pdf]

nature portfolio

Peer Review File

FACED 2.0 enables large-scale voltage and calcium imaging in vivo

Corresponding Author: Professor Na Ji

This file contains all editorial decision letters in order by version, followed by all author rebuttals in order by version.

A version of this paper was originally rejected for publication by Nature Methods, however that decision was reconsidered after appeal by the authors.

Version 0:

Decision Letter:

10th Jul 2024

Dear Na,

Thank you for your patience. Your Brief Communication entitled "FACED 2.0 enables large-scale voltage and calcium imaging in vivo" has now been seen by two reviewers, whose comments are attached. While they find your work of potential interest, they have raised serious concerns which in our view are sufficiently important that they preclude publication of the work in Nature Methods, at least in its present form.

As you will see, the reviewers raise concerns about pixel crosstalk and quality of the recorded traces. Should further experimental data allow you to fully address these criticisms we would be willing to look at a revised manuscript (unless, of course, something similar has by then been accepted at Nature Methods or appeared elsewhere). This includes submission or publication of a portion of this work somewhere else. We hope you understand that until we have read the revised paper in its entirety we cannot promise that it will be sent back for peer-review.

If you are interested in revising this manuscript for submission to Nature Methods in the future, please contact me to discuss your appeal before making any revisions. Otherwise, we hope that you find the reviewers' comments helpful when preparing your paper for submission elsewhere.

Best regards,
Nina

Nina Vogt, PhD
Senior Editor
Nature Methods

Reviewers' Comments:

Reviewer #2:
Remarks to the Author:
In this paper, Zhong et al. present FACED 2.0, an optical method for large-scale fluorescence imaging at high-speed in two and three dimensions. The presented approach incorporates hardware and software improvements compared to a previous technique (FACED 1.0) published by the same group (Wu et al. 2020). FACED 2.0 achieves pixel rate of 1 x 10^8 pixels/s and maintains the high spatial resolution typical of traditional 2P imaging scopes. The authors applied FACED 2.0 to monitor cerebral blood flow (Fig. 1), to measure signals from fluorescent voltage (Fig. 2) and calcium (Fig. 3) indicators in the mouse brain and to measure signals from fluorescent calcium indicators in the zebrafish brain (Suppl. Fig. 14-15). The data presented in the manuscript demonstrate: i) single-plane population voltage imaging from about 200 neurons in the mouse visual cortex and recording of supra- and sub-threshold cellular responses during visual stimulation; ii) volumetric population imaging of about 14000 neurons using genetically encoded calcium indicators in the mouse visual cortex; iii) population calcium imaging

of about 4700 neurons in zebrafish larvae. Performance of FACED 2.0 and its technical features are compared to those of other methods (including FACED 1.0) in Supplementary Table 1. Overall, this study presents important advancements for the field and relevant proof-of-principle applications of the improved method, which are of potential interest for the broad audience of Nature Methods. In my opinion, achievements of FACED 2.0 experiments in the mouse brain are very relevant to Circuit Neuroscience research, probably more than the technical improvements introduced by FACED 2.0 are to Applied Optics and Neurophotonics. The manuscript is well written and the results, in general, clearly presented. Below, please find a series of points to be addressed to improve the manuscript.

Major comments:
1) Raw traces of voltage and calcium indicators in mouse recordings are hardly visible in all current figures. Representative experimental traces from mouse recordings should be plotted at an expanded scale (similarly to what done in Suppl. Fig. 14 and 15 for the zebrafish data) to properly evaluate signal quality. This is particularly relevant for supra- and sub-threshold responses of voltage indicators. Example waveforms should be shown in both Fig. 2 and Fig. 3. This is an important first step to evaluate the quality of the recorded raw data.
2) Quantitative evaluation of signal-to-noise ratio (SNR) should be performed as a function of the imaging conditions (e.g., SNR vs photon/pixel). This is particularly important for sub-threshold responses of genetically encoded voltage indicators, which have very small amplitude (close to noise level). As the authors themselves underline, compared to electrophysiological approaches one key advantage of fast imaging approaches applied to fluorescence voltage indicators is the possibility to measure at the same time the sub- and supra-threshold response in a large number of cells (in this manuscript about 200 neurons) with cellular resolution. However, this advantage can be exploited only if the SNR of sub- and supra-threshold responses is high enough.
3) At the same laser repetition rate, FACED 2.0 enables acquiring 20% larger pixel/s values compared to FACED 1.0 (1x10^8 pixels/s vs 0.8x10^8 pixels/s), yet the authors are here able to record from much larger number of neurons compared to the number of neurons recorded in their previous publication (Wu et al. 2020). It thus appears important to quantify and discuss how much higher performance in imaging the large-scale population of neurons presented in the current study was achieved sacrificing SNR vs improving the optical method.
4) Results section: “the continual Z movement during image acquisition did not generate any in-frame motion artifacts”. This should be quantified and demonstrated.
5) Results, voltage indicator imaging section: it is stated that 4% photo-bleaching was observed during each 8s trial and that an overall photo-bleaching of 9% was observed across the entire 296 s-long recording. Please provide a graph to show the photo-bleaching amplitude as a function of trial number within the recording session.
6) Any sign of photo-damage introduced by FACED 2.0?
7) What is the group velocity dispersion of laser pulses in FACED 2.0? Was the laser beam pre-compensated? If I am not mistaken, in FACED 1.0 pre-chirping was used, but I could not find clear indication about pre-compensation in this study.
8) The system needs to be aligned every 5-30 minutes. This is quite a wide range of short durations, which may partially overlap with the duration of recording sessions (e.g., the 296 s-long recording presented in this study). Is there a way to monitor the quality of the alignment during the recording session?
9) The manuscript would greatly gain by the addition of a supplementary text critically describing the improvement of FACED 2.0 compared to FACED 1.0, while referring to Suppl. Table 1.
10) The software for data acquisition and system control is not shared.

Minor comments
1) In the methods, please add description of PSF measurements, including details about average laser power across conditions.
2) Please add dispersion quantification (e.g., standard deviation) to black dots in Fig. 2e.
3) Is laser power modulated during volumetric imaging? Please specify in methods.
4) Methods section: with regard to the formula “vn+1=vn + an”. “vn” indicates velocity and “an” indicates acceleration. The term “an” should be multiplied by “t” (time) to be in the same unit (m/s) as velocity. Please amend.
5) Suppl. Fig. 1: the red and pink colors are difficult to distinguish. Please consider changing the color code.
6) Please provide an explanation for the trend of the M and H traces to grow asymmetrically from negative values of the position to positive values (Supp. Fig. 2n and Suppl. Fig. 3n), while the opposite trend for L, H, and M traces is observed in Suppl. Fig. 2s and Suppl. Fig. 3s.

Reviewer #3:
Remarks to the Author:
Zhong and colleagues upgrade the free-space FACED two-photon fluorescence microscope (2PFM) and demonstrate its improved capabilities by performing in-vivo (1) large field-of-view (FOV) measurements of cerebral blood flow, (2) population (> 200 neurons simultaneously) voltage imaging at 385 Hz frame rate, and (3) volumetric calcium imaging of ~14,000 neurons down to 750 µm at 3.8 Hz volume rate in the mouse brain, as well as (4) “whole-brain” calcium imaging of zebrafish larvae. The FACED module uses a cylindrical lens to focus a laser beam into a cavity made by a pair of high-reflectivity mirrors that are slightly misaligned but otherwise parallel, producing an array of beamlets that are both temporally delayed and angularly separated. This array of beamlets forms a line scan under a typical 2PFM, and thus the 1-MHz laser repetition rate is effectively turned into a line scan rate (along the fast axis, or FACED scanning direction). When combined with a galvanometer that scans orthogonally (along the slow axis), the microscope achieves a kilohertz frame rate and can be applied to fast two-photon (2P) imaging. Compared to the original FACED microscope, FACED 2.0 reduces the temporal spacing between the beamlets to 1 ns, adds another galvo scanner along the FACED scanning direction to tile multiple adjoining scanning areas to one large FOV (e.g., 320 µm x 400 µm at 385 Hz frame rate), and uses a fast piezo motor to the objective to enable large-scale volumetric imaging (e.g., 1111 µm x 1000 µm x 400 µm at 3.8 Hz volume rate), with subcellular resolution (PSF < 1 µm lateral FWHM, and

< 5 µm axial FWHM).
The FACED microscope is impressive in leveraging its ultra-high laser point scanning speed to image a large area/volume at a high speed. Most notably, simultaneous large-scale voltage imaging reveals synchronized subthreshold membrane potential oscillations at 3-6 Hz across groups of neurons, in good agreement with recently published voltage imaging results by a target-illumination confocal microscope [1]. Also, cerebral capillaries and calcium imaging down to 750 µm by the FACED microscope reaches the depth limit of a 2PFM.
Overall, this paper is well-structured, the ideas are clearly presented and easy to follow, and the methods descriptions are very detailed. However, I believe the authors may have overstated the performance and impact of the FACED microscope because of two major concerns in this work.
(1) Although the excitation pulses are well-separated spatially (0.8-um inter-pulse separation, comparable to the lateral FWHM resolution), their temporal delays of 1 ns are too short for the ~3-ns fluorescence lifetime of GFP-based fluorophores (such as JEDI-2P and GCaMP6s used in this work), leading to enormous pixel crosstalk on the detection side (i.e., the information obtained between adjacent pixels are largely the same). This will deteriorate the spatial resolution and effectively reducing the number of resolvable points by at least 3 times (1 ns vs 3 ns). Sampling much faster than the fluorescence lifetime is ineffective for improving the imaging speed.
(2) One impactful achievement that the authors intend to claim is that FACED microscope allows for optical recordings of both suprathreshold and subthreshold activities in > 200 individual neurons simultaneously. This claim is not convincing. First, to capture the temporal dynamics of ~2.1 ms FWHM in the action potential, the 2.6 ms/frame is too slow. The effect can be seen when one compares the voltage traces with those in the original FACED paper obtained with ASAP3, an indicator considered inferior to the JEDI-2P used in this work. The “spikes” detected in the voltage traces in this work look much less distinguishable than those in the FACED 1.0 work. It seems like the authors simply lower the spike detection threshold from SNR > 7.5 (FACED 1.0) to SNR > 2.5 (FACED 2.0, this work). Furthermore, the collected photon numbers per neuron per frame (171 photons at 75-th percentile) attest to the low SNRs of voltage traces. Assuming photon shot noise limit, a -20 % deltaF over F translates into an SNR of ~2.6, meaning the voltage spiking activities can only be detected with subpar or even questionable fidelity, in only a quarter of the neurons imaged. In fact, if one inadvertently inverts the voltage traces, the spike detection algorithm may still report a good number of “activities” in many of the neurons imaged. If this is the case, then the suprathreshold voltage data should not be considered trustworthy. Also because of the low photon counts, the authors’ statement in the summary paragraph about the “dark state relaxation enhancing the photon yield by orders of magnitude” may not apply here either. Finally, when compared to similar large-scale voltage imaging work by other research groups, such as ref [1], the voltage imaging data presented here suffers from poor SNRs, and has no advancements in depth penetration or imaging FOV. However, the large-scale voltage imaging experiments in this work have their merit in detecting synchronized population-wide subthreshold oscillations at 3-6 Hz, which requires a much slower frame rate.
Therefore, unless the two major concerns described above are resolved, I would not recommend publication of this work as it is. There are also other points in the manuscript that the authors should also address:
About the imaging experiments:
• Even after normalization, the discontinuity in both spatial structure and laser illumination between adjoining areas in the FACED scan direction is visible. How do the authors deal with fluorescence fluctuations, say in neurons along the edges of tiled FACED scan, caused by motion artifacts convoluted with this discontinuity?
• In Fig. 2e, why is the firing rate discrete (i.e. only 0, 100 Hz, and 200 Hz)?
• Given the axial PSF and the voxel size, the microscope cannot image the entire volume; i.e. the axial sampling is not dense enough to cover every plane in the volume. It is therefore misleading to claim the volume size or whole-brain volumetric imaging of zebrafish larvae.
• For Figs. 1 and S4 to S6, 1035 nm was used for imaging Thy1-GFP mice. The 2P cross section at 1035 nm for GFP is very low compared to 920 nm. What’s the reason to use 1035 nm instead of 920 nm? Is this for deeper penetration? Given the much lower 2P cross section, is this a better choice to image deeper?
• It appears that the same excitation power was used for imaging for all depth (150 to 200 mW), but the number of frames averaged was increased for deeper imaging, for example, from 400 (S4) to 4000 (S5 and S6). For imaging at 750 to 800 um depth, however, the signal would decrease by ~1000x. Therefore, this 10x increase in time cannot compensate for the increase in imaging depth unless ~ 100x less signal was obtained in the deepest regions of the 3D volume. Please quantify the image quality in the deepest regions in terms of photons/frame.
•
About the microscope:
• What is the power throughput of the FACED module + microscope?
About the manuscript:
• In supp. table 1, it is meaningless to compare pixels/voxels per unit time. Comparison of the number of resolvable points per unit time is more reasonable. Also, the authors tend to understate the performance of other microscopes. Simple examples include maximum imaging depths of 600 µm in Light Beads Microscopy, and 540 µm in Scan Multiplier Unit. Instead, the table puts 500 µm for both works. There may be more instances. If this table is to be published, please make sure it is correct.
• In supp. Table 4, data is missing for the rest of the figures.

References
[1] Xiao, S. et al. Large-scale deep tissue voltage imaging with targeted-illumination confocal microscopy. Nat Methods 21, 1094–1102 (2024).

Although we cannot publish your paper in its current form, it may be appropriate for another journal in the Nature Portfolio. If you wish to explore the journals and transfer your manuscript please use our manuscript transfer

portal. You will not have to re-supply manuscript metadata and files, unless you wish to make modifications. For more information, please see our manuscript transfer FAQ page.

** For Nature Portfolio general information and news for authors, see http://npg.nature.com/authors.

Version 1:

Decision Letter:

16th Jan 2025

Dear Na,

Thank you for your letter asking us to reconsider our decision on your Brief Communication, "FACED 2.0 enables large-scale voltage and calcium imaging in vivo". After careful consideration we have decided that we are willing to consider a revised version of your manuscript.

Please note that I realize that you have included the revised manuscript, but I am sending an appeal-yes decision, so that you can make sure all relevant files including the videos and revised checklists are included in the resubmitted material.

When resubmitting your paper:

* include a point-by-point response to our referees and to any editorial suggestions

* please underline/highlight any additions to the text or areas with other significant changes to facilitate review of the revised manuscript

* address the points listed described below to conform to our open science requirements

* ensure it complies with our general format requirements as set out in our guide to authors at www.nature.com/naturemethods

* resubmit all the necessary files electronically by using the link below to access your home page

Link Redacted

Note: This URL links to your confidential home page and associated information about manuscripts you may have submitted, or that you are reviewing for us. If you wish to forward this email to co-authors, please delete the link to your homepage.

We hope to receive your revised paper within one month. If you cannot send it within this time, please let us know. In this event, we will still be happy to reconsider your paper at a later date so long as nothing similar has been accepted for publication at Nature Methods or published elsewhere.

OPEN SCIENCE REQUIREMENTS

REPORTING SUMMARY AND EDITORIAL POLICY CHECKLISTS
When revising your manuscript, please submit reporting summary and editorial policy checklists.

Reporting summary: https://www.nature.com/documents/nr-reporting-summary.zip
Editorial policy checklist: https://www.nature.com/documents/nr-editorial-policy-checklist.zip

If your paper includes custom software, we also ask you to complete a supplemental reporting summary.

Software supplement: https://www.nature.com/documents/nr-software-policy.pdf

Please submit these with your revised manuscript. They will be available to reviewers to aid in their evaluation if the paper is re-reviewed. If you have any questions about the checklist, please see http://www.nature.com/authors/policies/availability.html or contact me.

Please note that these forms are dynamic ‘smart pdfs’ and must therefore be downloaded and completed in Adobe Reader. We will then flatten them for ease of use by the reviewers. If you would like to reference the guidance text as you complete the template, please access these flattened versions at http://www.nature.com/authors/policies/availability.html.

Finally, please ensure that you retain unprocessed data and metadata files after publication, ideally archiving data in perpetuity, as these may be requested during the peer review and production process or after publication if any issues arise.

DATA AVAILABILITY
Please include a “Data availability” subsection in the Online Methods. This section should inform readers about the availability of the data used to support the conclusions of your study, including accession codes to public repositories, references to source data that may be published alongside the paper, unique identifiers such as URLs to data repository entries, or data set DOIs, and any other statement about data availability. At a minimum, you should include the following statement: “The data that support the findings of this study are available from the corresponding author upon request”, describing which data is available upon request and mentioning any restrictions on availability. If DOIs are provided, please include these in the Reference list (authors, title, publisher (repository name), identifier, year). For more guidance on how to write this section please see:
http://www.nature.com/authors/policies/data/data-availability-statements-data-citations.pdf

CODE AVAILABILITY
Please include a “Code Availability” subsection in the Online Methods which details how your custom code is made available. Only in rare cases (where code is not central to the main conclusions of the paper) is the statement “available upon request” allowed (and reasons should be specified).

We request that you deposit code in a DOI-minting repository such as Zenodo, Gigantum or Code Ocean and cite the DOI in the Reference list. We also request that you use code versioning and provide a license.

For more information on our code sharing policy and requirements, please see:
https://www.nature.com/nature-research/editorial-policies/reporting-standards#availability-of-computer-code

MATERIALS AVAILABILITY
As a condition of publication in Nature Methods, authors are required to make unique materials promptly available to others without undue qualifications.

Authors reporting new chemical compounds must provide chemical structure, synthesis and characterization details. Authors reporting mutant strains and cell lines are strongly encouraged to use established public repositories.

More details about our materials availability policy can be found at https://www.nature.com/nature-portfolio/editorial-policies/reporting-standards#availability-of-materials

ORCID
Nature Methods is committed to improving transparency in authorship. As part of our efforts in this direction, we are now requesting that all authors identified as ‘corresponding author’ on published papers create and link their Open Researcher and Contributor Identifier (ORCID) with their account on the Manuscript Tracking System (MTS), prior to acceptance. This applies to primary research papers only. ORCID helps the scientific community achieve unambiguous attribution of all scholarly contributions. You can create and link your ORCID from the home page of the MTS by clicking on ‘Modify my Springer Nature account’. For more information please visit please visit www.springernature.com/orcid.

We look forward to hearing from you soon.

Best regards,
Nina

Nina Vogt, PhD
Senior Editor
Nature Methods

Version 2:

Decision Letter:

14th Mar 2025

Dear Na,

Thank you for your patience. Your Brief Communication, "FACED 2.0 enables large-scale voltage and calcium imaging in vivo", has now been seen by two reviewers. As you will see from their comments below, although the reviewers find your work of considerable potential interest, they have raised a number of concerns. We are interested in the possibility of publishing your paper in Nature Methods, but would like to consider your response to these concerns before we reach a final decision on

publication. We therefore invite you to revise your manuscript to address these concerns.

We are committed to providing a fair and constructive peer-review process. Do not hesitate to contact us if there are specific requests from the reviewers that you believe are technically impossible or unlikely to yield a meaningful outcome.

When revising your paper:

* include a point-by-point response to the reviewers and to any editorial suggestions

* please underline/highlight any additions to the text or areas with other significant changes to facilitate review of the revised manuscript

* address the points listed described below to conform to our open science requirements

* ensure it complies with our general format requirements as set out in our guide to authors at www.nature.com/naturemethods

* resubmit all the necessary files electronically by using the link below to access your home page

Link Redacted

Note: This URL links to your confidential home page and associated information about manuscripts you may have submitted, or that you are reviewing for us. If you wish to forward this email to co-authors, please delete the link to your homepage.

We hope to receive your revised paper within two months. If you cannot send it within this time, please let us know. In this event, we will still be happy to reconsider your paper at a later date so long as nothing similar has been accepted for publication at Nature Methods or published elsewhere.

OPEN SCIENCE REQUIREMENTS

REPORTING SUMMARY AND EDITORIAL POLICY CHECKLISTS
When revising your manuscript, please update your reporting summary and editorial policy checklists.

Reporting summary: https://www.nature.com/documents/nr-reporting-summary.zip
Editorial policy checklist: https://www.nature.com/documents/nr-editorial-policy-checklist.zip

If your paper includes custom software, we also ask you to complete a supplemental reporting summary.

Software supplement: https://www.nature.com/documents/nr-software-policy.pdf

Please submit these with your revised manuscript. They will be available to reviewers to aid in their evaluation if the paper is re-reviewed. If you have any questions about the checklist, please see http://www.nature.com/authors/policies/availability.html or contact me.

Please note that these forms are dynamic 'smart pdfs' and must therefore be downloaded and completed in Adobe Reader. We will then flatten them for ease of use by the reviewers. If you would like to reference the guidance text as you complete the template, please access these flattened versions at http://www.nature.com/authors/policies/availability.html.

Finally, please ensure that you retain unprocessed data and metadata files after publication, ideally archiving data in perpetuity, as these may be requested during the peer review and production process or after publication if any issues arise.

EXTENDED DATA FIGURES
When re-submitting your manuscript, please ensure that any supplementary figures and tables that are crucial to the manuscript's conclusions are converted into Extended Data figures and tables to increase visibility of these data. Extended Data figures and tables are online-only (present in the online PDF and full-text HTML versions of the paper), peer-reviewed display items that provide essential background to the article but are not included in the main article due to space constraints. A maximum of ten Extended Data display items (figures and tables) is permitted.

DATA AVAILABILITY
We strongly encourage you to deposit all new data associated with the paper in a persistent repository where they can be freely and enduringly accessed. We recommend submitting the data to discipline-specific and community-recognized repositories; a list of repositories is provided here: http://www.nature.com/sdata/policies/repositories

All novel DNA and RNA sequencing data, protein sequences, genetic polymorphisms, linked genotype and phenotype data, gene expression data, macromolecular structures, and proteomics data must be deposited in a publicly accessible database, and accession codes and associated hyperlinks must be provided in the “Data Availability” section.

Refer to our data policies here: https://www.nature.com/nature-research/editorial-policies/reporting-standards#availability-of-data

To further increase transparency, we encourage you to provide, in tabular form, the data underlying the graphical representations used in your figures. This is in addition to our data-deposition policy for specific types of experiments and large datasets. For readers, the source data will be made accessible directly from the figure legend. Spreadsheets can be submitted in .xls, .xlsx or .csv formats. Only one (1) file per figure is permitted: thus if there is a multi-paneled figure the source data for each panel should be clearly labeled in the csv/Excel file; alternately the data for a figure can be included in multiple, clearly labeled sheets in an Excel file. File sizes of up to 30 MB are permitted. When submitting source data files with your manuscript please select the Source Data file type and use the Title field in the File Description tab to indicate which figure the source data pertains to.

Please include a “Data availability” subsection in the Online Methods. This section should inform readers about the availability of the data used to support the conclusions of your study, including accession codes to public repositories, references to source data that may be published alongside the paper, unique identifiers such as URLs to data repository entries, or data set DOIs, and any other statement about data availability. At a minimum, you should include the following statement: “The data that support the findings of this study are available from the corresponding author upon request”, describing which data is available upon request and mentioning any restrictions on availability. If DOIs are provided, please include these in the Reference list (authors, title, publisher (repository name), identifier, year). For more guidance on how to write this section please see: http://www.nature.com/authors/policies/data/data-availability-statements-data-citations.pdf

CODE AVAILABILITY
Please include a “Code Availability” subsection in the Online Methods which details how your custom code is made available. Only in rare cases (where code is not central to the main conclusions of the paper) is the statement “available upon request” allowed (and reasons should be specified).

We request that you deposit code in a DOI-minting repository such as Zenodo, Gigantum or Code Ocean and cite the DOI in the Reference list. We also request that you use code versioning and provide a license.

For more information on our code sharing policy and requirements, please see: https://www.nature.com/nature-research/editorial-policies/reporting-standards#availability-of-computer-code

MATERIALS AVAILABILITY
As a condition of publication in Nature Methods, authors are required to make unique materials promptly available to others without undue qualifications.

Authors reporting new chemical compounds must provide chemical structure, synthesis and characterization details. Authors reporting mutant strains and cell lines are strongly encouraged to use established public repositories.

More details about our materials availability policy can be found at https://www.nature.com/nature-portfolio/editorial-policies/reporting-standards#availability-of-materials

ORCID
Nature Methods is committed to improving transparency in authorship. As part of our efforts in this direction, we are now requesting that all authors identified as ‘corresponding author’ on published papers create and link their Open Researcher and Contributor Identifier (ORCID) with their account on the Manuscript Tracking System (MTS), prior to acceptance. This applies to primary research papers only. ORCID helps the scientific community achieve unambiguous attribution of all scholarly contributions. You can create and link your ORCID from the home page of the MTS by clicking on ‘Modify my Springer Nature account’. For more information please visit please visit www.springernature.com/orcid.

Please do not hesitate to contact me if you have any questions or would like to discuss these revisions further. We look forward to seeing the revised manuscript and thank you for the opportunity to consider your work.

Best regards,
Nina

Nina Vogt, PhD
Senior Editor
Nature Methods

Reviewers' Comments:

Reviewer #2 (Remarks to the Author):

In this revised version, the authors addressed most of my previous concerns. The manuscript is significantly improved and can be, in my opinion, of interest for the broad audience of Nature Methods.
I have a few minor points that should be considered:
1) Response to previous point #2 of this referee: while I understand that the filtering process makes it difficult to estimate the SNR on subthreshold activity, I think that stating that subthreshold signals are of good quality is not enough. Perhaps the authors can plot the amplitude distribution of the subthreshold signal to provide a more quantitative assessment of the quality of this signal.
2) Current Supplementary Figure 1: please provide spatial scale and intensity scale. Is the intensity scale the same in the ground truth (left) and volumetric (right) images?
3) A short text should be added to the discussion stating that 1035 nm laser excitation was used in the study and therefore that the performance of FACED 2.0 should significantly improve when 920 nm laser light will be used to excite JEDI-2P and GCaMP6s.
4) A short sentence should be added to the discussion stating the limitation of FACED regarding the need of regular and frequent alignment (every 5-30 minutes). This is critical information for experimenters willing to implement FACED 2.0 in their laboratories.

Reviewer #3 (Remarks to the Author):

We appreciate the authors' careful responses to all concerns, questions, and comments raised by both reviewers. To improve the quality of the data and enhance the credibility of the methods, the authors corrected their photon counts calibrations, performed new voltage imaging experiments which yielded higher photon counts (1.5 x) at double the frame rate, and compared the voltage imaging data obtained at 800 Hz and 400 Hz. Upon reading the additional data and re-examining the entire manuscript, we believe that the major issues are still not resolved. We list them below.
1. Compared to FACED1, FACED2 reduced the pulse (pixel) separation from 2 ns to 1 ns. The authors claimed that this is one of the main improvements of FACED2. We respectfully disagree.
The short delay time between adjacent pixels using FACED microscopes is a major limitation for imaging fluorescence, with most fluorescent probes have fluorescence lifetimes of 2 to 6 ns. For GFP-based probes (lifetime ~ 3 ns), and at 1 ns delay between pulses, the fluorescence overlap between adjacent pixels is ~ 72%. Put it in another way, the adjacent pixels mostly have the same information even though they are spatially separated. Therefore, the number of independent pixels per line is effectively reduced by ~ 2x. One may argue that the overlap in fluorescence can be subtracted out, but this will inevitably increase the photon noise. Depending on the signal strength difference between the adjacent pixels, this additional photon noise can be overwhelming.
The authors argued that this overlap in fluorescence may not be important for imaging the soma where the pixels are binned anyway. This statement essentially says that when imaging the soma, a high spatial resolution is not important. While this may be true in some situations, it remains a major limitation as a fluorescence microscope. In addition, if a low spatial resolution can be used for imaging the voltage signal, a much better way is to use a larger focal spot and higher pulse energy (e.g., twice the spot area with twice the pulse energy at half the "effective repetition rate"). Due to the two-photon response, the resulting signal will be higher. Therefore, to improve from FACED1, FACED2 should not reduce the time separation between the pulses to 1 ns to increase the nominal number of pixels per line (even 2 ns is too short for many fluorescent probes). Instead, FACED2 should have enlarged the focal spot size and used less number of foci (while maintaining the same average power) to maximize the performance of FACED microscope.
The authors went on to claim that "this (1 ns separation between the pulses) is a feature, not a bug". We disagree. We would like the authors to compare (1) FACED with 1 ns pulse separation and 100 foci vs (2) a setup (e.g., reverb or lightbeads, not using FACED) with 10 ns pulse separation and 100 foci, which will fill the 1000 ns between the pulses of a 1-MHz laser. What is the advantage of (1) FACED approached with 1 ns delay vs (2)? If there is no advantage, then the short delay time between adjacent pulses is a limitation of the FACED approach, not a useful feature.

2. The quantification of the SNR of the voltage and Ca imaging. For fast and/or large scale recording of neuronal activity, the SNR is perhaps the most important parameter to quantify the performance of the system. We found the revised manuscript still does not provide adequate quantifications of the SNR, and the SNRs we calculated from the data reported appear to be too low.
The SNR of the voltage recording should be calculated directly from the photon counts per neuron per frame (F) and the measured (or reported) delta F/F for the probe . The main text cited a peak delta F over F0 of ~ 20 %, which is consistent with the previously reported AP response of JEDI-2P-Kv. With a reported photon counts of ~ 300 photons/neuron/frame (Supplementary Table 5, 50% percentile), the calculated SNR should be ~ 3.5. This is substantially below the SNR reported for FACED1. We would like the authors to directly compare the SNR of FACED2 vs FACED1 to show improvement,
For Ca-imaging, we can convert the reported pixel values (average of pixel values in
the top 99% of image frame, Supplementary Table 4) to photon counts using the calibration factor (~ 1300) provided by the authors. For example, at 517 um depth, the photons/neuron/frame is (195/1300)*100 ~ 15 (or ~ 41 photons/neuron/s), which is not sufficient for Ca-imaging (using the d' calculation for GCaMP6s). Therefore, data for neuronal activity recording at depth beyond 500 um has too low an SNR to be useful. To show the ability to image throughout the 750 um depth, the authors need to show, for each depth, the distribution of the d' values for the calcium data.

3. The use of a constant excitation power throughout the imaging depth of ~ 750 um.
This approach for imaging a 3D volume is very unusual, and it is certainly not optimum. It's not clear why the authors did this instead of increasing the excitation power with increasing imaging depth. We would like the authors to provide some reasons for this approach. Even more mysterious is the fact that the authors reported a total fluorescence signal attenuation of ~50x (GCaMP6s, 920 nm excitation, Supplementary Table 4) from 97.5 um to ~ 757.5 um, and ~ 80x (1035 nm excitation, in the rebuttal letter) from ~ 50 um to ~ 750 um. These attenuation factors appear to be off by one order of magnitude, and these data will lead to scattering lengths for the mouse brain in vivo of ~ 340 um at 920 nm and ~320 nm at 1035 nm. These scattering lengths are 1.5x to 2x longer than typical data reported in the past at these wavelengths (by many research groups). Would this be measurement errors? The authors need to provide the explanations.
4. A minor point:
Please report the power throughput for the FACED module and the microscope separately. In addition, the authors need to report the intensity non-uniformity of FACED illumination line, which will also impact the recording SNR across the FOV.

Version 3:

Decision Letter:

Our ref: NMETH-A56364C

4th Sep 2025

Dear Na,

Thank you for submitting your revised manuscript "FACED 2.0 enables large-scale voltage and calcium imaging in vivo" (NMETH-A56364C). As I mentioned before, it has now been seen by the original referees and their comments are below. The reviewers find that the paper has improved in revision. Taking into account your responses you have provided to the remaining concerns, we'll be happy in principle to publish it in Nature Methods, pending minor revisions to satisfy the referees' final requests and to comply with our editorial and formatting guidelines.

I would like to send the responses you have provided to the remaining concerns to reviewer #3. Please let me know whether that's okay.

We are now performing detailed checks on your paper and will send you a checklist detailing our editorial and formatting requirements within two weeks or so. Please do not upload the final materials and make any revisions until you receive this additional information from us.

TRANSPARENT PEER REVIEW
Nature Methods offers a transparent peer review option for new original research manuscripts. We encourage increased transparency in peer review by publishing the reviewer comments, author rebuttal letters and editorial decision letters if the authors agree. Such peer review material is made available as a supplementary peer review file. **Please state in the cover letter 'I wish to participate in transparent peer review' if you want to opt in, or 'I do not wish to participate in transparent peer review' if you don't.** Failure to state your preference will result in delays in accepting your manuscript for publication.

Please note: we allow redactions to authors' rebuttal and reviewer comments in the interest of confidentiality. If you are concerned about the release of confidential data, please let us know specifically what information you would like to have removed. Please note that we cannot incorporate redactions for any other reasons. Reviewer names will be published in the peer review files if the reviewer signed the comments to authors, or if reviewers explicitly agree to release their name. For more information, please refer to our FAQ page.

ORCID
IMPORTANT: Non-corresponding authors do not have to link their ORCIDs but are encouraged to do so. Please note that it will not be possible to add/modify ORCIDs at proof. Thus, please let your co-authors know that if they wish to have their ORCID added to the paper they must follow the procedure described in the following link prior to acceptance:
https://www.springernature.com/gp/researchers/orcid/orcid-for-nature-research

Thank you again for your interest in Nature Methods. Please do not hesitate to contact me if you have any questions. We will be in touch again soon.

Best regards,
Nina

Nina Vogt, PhD
Senior Editor
Nature Methods

Reviewer #2 (Remarks to the Author):

The authors effectively addressed my remaining requests

Reviewer #3 (Remarks to the Author):

We thank the authors for their detailed responses. We believe there still exist issues to be discussed and clarified.
1. The quantification of the SNR of the voltage and Ca imaging.
For voltage imaging, the calculations of the SNR by the authors do not agree with our calculations. In our previous responses, we stated that the SNR of the voltage recording should be calculated directly from the photon counts per neuron per frame (F) and the measured (or reported) delta F/F for the probe. We calculated SNR = delta F/sqrt(F) = delta F/F * sqrt(F) = 20% [cited in the main text] * sqrt(300) [Supp Table 5, 50% percentile] ~= 3.5. However, the authors insisted on an SNR ~ 7.5 with the same definition. SNR is a crucial quantity that determines the recording quality, and these two drastically different quantifications of SNRs could lead to entirely opposite understandings of the performance. We would like the authors to point out what makes up the difference in the SNR calculations.
For volumetric Ca imaging, the SNR, or the d' value, as calculated in the previous response, is not sufficient for depths beyond 500 um (see DOI: https://doi.org/10.1016/j.bpj.2012.07.058 and https://doi.org/10.7554/eLife.53205). The authors claimed that the responses in calcium activities to the drifting grating stimuli are still visible to the algorithm and human eyes from a single trial which lasts 3-5 seconds or by averaging over multiple trials. This is true for repeating grating stimuli experiments, but for calcium imaging in general, the ability to reliably detect single, perhaps rarely repeatable activity events is still crucial. The FACED microscope presented here failed to demonstrate such a capability.
2. The improvement of FACED 2.0 vs FACED 1.0.
The authors replied that "the improvement from FACED 1.0 to FACED 2.0 was NOT about the increased number of independent pixels," but about enlarging the imaging FOV while sustaining the high axial resolution that is critical for high 2P fluorescence signals. Essentially, the larger imaging FOV comes at the expense of even higher pixel crosstalk (previously 2-ns interpixel delays and 1-ns interpixel delays now, compared to 3-ns to 4-ns fluorescence lifetime of common fluorophores), which effectively compromises the spatial resolution along the FACED scanning direction. While the authors argued in Supp Note 4 that the pixel crosstalk led to minimal contamination in neuronal activities, this feature/bug, unique to the FACED microscope, could preclude its use in the wider imaging applications.
For the reason discussed above, statements in the main text like "FACED 2.0 achieves high-speed imaging without sacrificing spatial resolution" are not true for the FACED-scanning direction. Modifications of such statements are needed to avoid misleading messages.
3. The use of a constant excitation power throughout an imaging depth of ~ 750 um is a bad practice and not justifiable under any circumstance. It should be explicitly stated as such to discouraged others from doing the same.
The authors chose not to vary the laser power with imaging depth because "doing so would require the inclusion and synchronization of a Pockel cell, which increases system cost and complexity." We believe many would agree that the integration of a Pockels cell is way less expensive or complex than building a FACED microscope. Many biological imaging labs have been equipped with Pockels cells in the system, and there exists commercial firmware that supports Pockels cell synchronization as a standard option.
The authors went on to cite publications that discredit the need to greatly vary the excitation power with imaging depth. However, some of these examples evidently show that a greater change in laser power is preferred. For example, in the authors' own previous work (FACED 1.0 in vivo blood circulation imaging, Ref. 7 in the authors' rebuttal on page 11), they instructed in the methods that "Given the finite half-life of injected fluorescent dyes (~2 h for 70-kDa dyes) and tissue attenuation of the excitation light, we first collected data from the deepest blood vessels and shifted the imaging plane gradually toward the brain surface." This indicates that the deeper blood vessels were inherently brighter than the superficial ones when imaging acquisition occurred. With a 5.3x for 671 um depth difference (should it be ~ 530 um because the deepest video was obtained from a different mouse/experiment?), the signal level or SNR of the videos/kymographs in deeper layers is visibly lower than that in shallow layers. Therefore, the drop in signal level in deeper regions, compounded with the fact that deeper blood vessels were inherently brighter, suggests that a greater difference in laser power across imaging depth is required to reach approximately uniform signal levels.
We also understand that the authors are able to integrate a Pockels cell into the system. Imaging a volume as thick as > 700 um without varying the laser power is a bad idea, and should not be encouraged or emulated.
To conclude, although the authors have demonstrated specific biological imaging experiments using FACED 2.0, judging from the angle of technology development, we don't see a substantial improvement of FACED 2.0 over FACED 1.0. The main technical disadvantages of the FACED method (i.e., the pixel cross talk due to the short time delay between adjacent pixels) is not addressed at all and could limit its adoption to the wider imaging community.

Reviewer #4 (Remarks to the Author):

I co-reviewed this manuscript with one of the reviewers who provided the listed reports. This is part of the Nature Methods initiative to facilitate training in peer review and to provide appropriate recognition for Early Career Researchers who co-review manuscripts.

Version 4:

Decision Letter:

21st Oct 2025

Dear Na,

I am pleased to inform you that your Article, "FACED 2.0 enables large-scale voltage and calcium imaging in vivo", has now been accepted for publication in Nature Methods. The received and accepted dates will be May 8th, 2024 and October 21st, 2025. This note is intended to let you know what to expect from us over the next month or so, and to let you know where to address any further questions.

Over the next few weeks, your paper will be copyedited to ensure that it conforms to Nature Methods style. Once your paper is typeset, you will receive an email with a link to choose the appropriate publishing options for your paper and our Author Services team will be in touch regarding any additional information that may be required. It is extremely important that you let us know now whether you will be difficult to contact over the next month. If this is the case, we ask that you send us the contact information (email, phone and fax) of someone who will be able to check the proofs and deal with any last-minute problems.

After the grant of rights is completed, you will receive a link to your electronic proof via email with a request to make any corrections within 48 hours. If, when you receive your proof, you cannot meet this deadline, please inform us at rjsproduction@springernature.com immediately.

Authors may need to take specific actions to achieve compliance with funder and institutional open access mandates. If your research is supported by a funder that requires immediate open access (e.g. according to Plan S principles or the NIH public access policy) then you should select the gold OA route, and we will direct you to the compliant route where possible. Because authors warrant under our subscription licensing terms that they haven’t committed to licensing any version of their article under a licence inconsistent with the terms of our agreement – including the applicable embargo period – publication under the subscription model isn’t suitable for authors whose funders require no embargo.

If you have any questions about our publishing options, costs, Open Access requirements, or our legal forms, please contact ASJournals@springernature.com

If you have posted a preprint on any preprint server, please ensure that the preprint details are updated with a publication reference, including the DOI and a URL to the published version of the article on the journal website.

You may wish to make your media relations office aware of your accepted publication, in case they consider it appropriate to organize some internal or external publicity. Once your paper has been scheduled you will receive an email confirming the publication details. This is normally 3-4 working days in advance of publication. If you need additional notice of the date and time of publication, please let the production team know when you receive the proof of your article to ensure there is sufficient time to coordinate. Further information on our embargo policies can be found here: https://www.nature.com/authors/policies/embargo.html

To assist our authors in disseminating their research to the broader community, our SharedIt initiative provides you with a unique shareable link that will allow anyone (with or without a subscription) to read the published article. Recipients of the link with a subscription will also be able to download and print the PDF.

As soon as your article is published, you will receive an automated email with your shareable link.

If you are active on Twitter/X or Bluesky, please e-mail me your and your coauthors’ handles so that we may tag you when the paper is published.

You can now use a single sign-on for all your accounts, view the status of all your manuscript submissions and reviews, access usage statistics for your published articles and download a record of your refereeing activity for the Nature journals.

Please note that you and any of your coauthors will be able to order reprints and single copies of the issue containing your article through Nature Portfolio's reprint website, which is located at http://www.nature.com/reprints/author-reprints.html. If there are any questions about reprints please send an email to author-reprints@nature.com and someone will assist you.

Please feel free to contact me if you have questions about any of these points.

Best regards,
Nina

Nina Vogt, PhD
Senior Editor
Nature Methods

** Visit the Springer Nature Editorial and Publishing website at <a href="http://editorial-jobs.springernature.com?

utm_source=ejP_NMeth_email&utm_medium=ejP_NMeth_email&utm_campaign=ejp_Nmeth">www.springernature.com/editorial-and-publishing-jobs for more information about our career opportunities. If you have any questions please click here.**

Open Access This Peer Review File is licensed under a Creative Commons Attribution 4.0 International License, which permits use, sharing, adaptation, distribution and reproduction in any medium or format, as long as you give appropriate credit to the original author(s) and the source, provide a link to the Creative Commons license, and indicate if changes were made. In cases where reviewers are anonymous, credit should be given to 'Anonymous Referee' and the source.
The images or other third party material in this Peer Review File are included in the article's Creative Commons license, unless indicated otherwise in a credit line to the material. If material is not included in the article's Creative Commons license and your intended use is not permitted by statutory regulation or exceeds the permitted use, you will need to obtain permission directly from the copyright holder.
To view a copy of this license, visit https://creativecommons.org/licenses/by/4.0/

We thank both reviewers for their helpful comments and suggestions, which have motivated us to acquire new data and perform additional analyses. Below, we provide detailed point-by-point responses and indicate where revisions were made in the updated manuscript (text in red). We trust that the revisions have addressed the reviewers' concerns and hope the revised manuscript will now be considered suitable for publication in *Nature Methods*.

Reviewer #2:

Remarks to the Author:

In this paper, Zhong et al. present FACED 2.0, an optical method for large-scale fluorescence imaging at high-speed in two and three dimensions. The presented approach incorporates hardware and software improvements compared to a previous technique (FACED 1.0) published by the same group (Wu et al. 2020). FACED 2.0 achieves pixel rate of 1 x 10^8 pixels/s and maintains the high spatial resolution typical of traditional 2P imaging scopes. The authors applied FACED 2.0 to monitor cerebral blood flow (Fig. 1), to measure signals from fluorescent voltage (Fig. 2) and calcium (Fig. 3) indicators in the mouse brain and to measure signals from fluorescent calcium indicators in the zebrafish brain (Suppl. Fig. 14-15). The data presented in the manuscript demonstrate: i) single-plane population voltage imaging from about 200 neurons in the mouse visual cortex and recording of supra- and sub-threshold cellular responses during visual stimulation; ii) volumetric population imaging of about 14000 neurons using genetically encoded calcium indicators in the mouse visual cortex; iii) population calcium imaging of about 4700 neurons in zebrafish larvae. Performance of FACED 2.0 and its technical features are compared to those of other methods (including FACED 1.0) in Supplementary Table 1. Overall, this study presents important advancements for the field and relevant proof-of-principle applications of the improved method, which are of potential interest for the broad audience of Nature Methods. In my opinion, achievements of FACED 2.0 experiments in the mouse brain are very relevant to Circuit Neuroscience research, probably more than the technical improvements introduced by FACED 2.0 are to Applied Optics and Neurophotonics. The manuscript is well written and the results, in general, clearly presented. Below, please find a series of points to be addressed to improve the manuscript.

Major comments:

1) Raw traces of voltage and calcium indicators in mouse recordings are hardly visible in all current figures. Representative experimental traces from mouse recordings should be plotted at an expanded scale (similarly to what done in Suppl. Fig. 14 and 15 for the zebrafish data) to properly evaluate signal quality. This is particularly relevant for supra- and sub-threshold responses of voltage indicators. Example waveforms should be shown in both Fig. 2 and Fig. 3. This is an important first step to evaluate the quality of the recorded raw data.

We appreciate the reviewer’s suggestion.

We acquired new voltage imaging datasets at 800 Hz and 385 Hz (new Fig. 2 and Extended Data Fig. 11) and added the voltage traces with expanded scale in these figures. In Extended Data Figs. 8 and 12, we also provided represented traces for all neurons imaged in these datasets. We note that although these datasets have higher signals than the original Fig. 2, the biological conclusions remain the same.

For calcium imaging data in Fig. 3, in addition to the example calcium traces presented in the original Supplementary Fig. 16 (now Extended Data Fig. 20), we included example traces in Fig. 3e.

These figures are reproduced below.

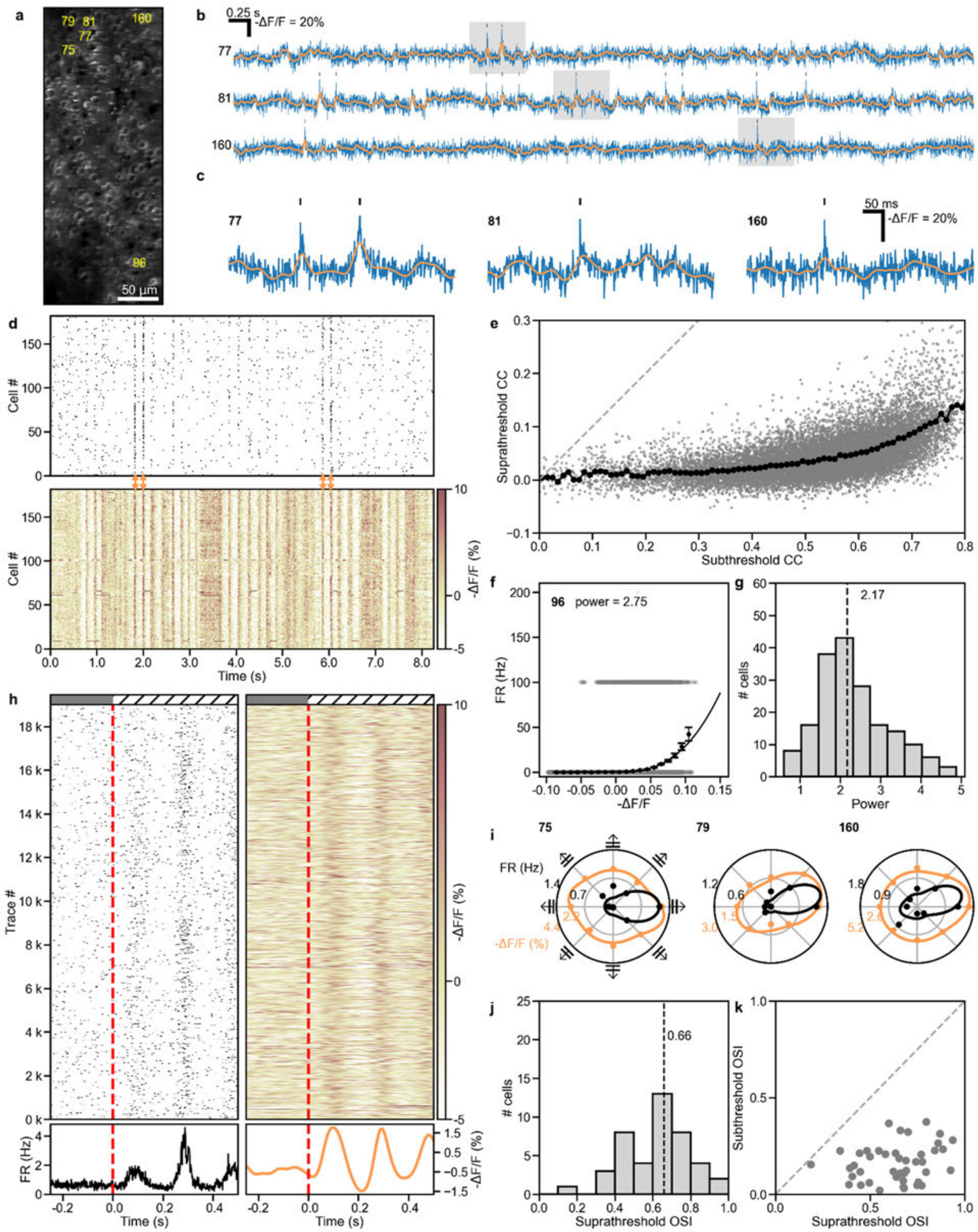

Figure 2. Imaging voltage activity from population of neurons *in vivo* at 1.25 ms/frame. (**a**) JEDI-2P-kv-expressing L2/3 neurons in a 160 µm × 400 µm FOV within a mouse visual cortex imaged at 132 µm depth and 1.25 ms/frame. Pixel size: 0.8 µm × 0.8 µm. 1035 nm excitation at 163 mW post 1.05-NA objective. Numbers: representative neurons. (**b**) Representative -ΔF/F voltage traces (in blue) of numbered neurons in **a**. Black ticks: suprathreshold spikes. Orange traces: subthreshold -ΔF/F. (**c**) Zoom-in view of voltage traces within the gray boxes in **b**. (**d**) Raster plots of (top) spikes and (bottom) subthreshold -ΔF/F traces of 182 neurons during a representative 8.25-s trial. (**e**) Suprathreshold versus subthreshold pairwise correlation coefficients (CC, gray dots) for 16,471 cell pairs from the 182 neurons in **a**. Black dots: binned averages with 0.01 bin size for subthreshold CC. Dashed gray line: reference line with slope = 1. (**f**) Plot of firing rate (FR) vs subthreshold -ΔF/F for Neuron 96, fit with a power-law function (black curve; power-law exponent = 2.75). Gray dots: FR vs subthreshold -ΔF/F. Black dots: binned average FR with 0.01 bin size in -ΔF/F. Error bar: s.e.m. (**g**) Histogram of fitted power-law exponent values of 182 neurons. Dashed line: median. (**h**) Spikes (left) and subthreshold responses (right) relative to stimulus onset (red dashed lines) extracted from 19,008 voltage traces of 88 neurons exhibiting visually evoked activity across 27 trials × 8 drifting grating orientations. Top: raster plots of individual traces; Bottom: Average firing rate (black curve) and average subthreshold -ΔF/F (orange curve). (**i**) Representative polar-plot tuning curves for supra- (black) and subthreshold (orange) voltage responses of Neuron 75, 79, 160, respectively. Dots: measured responses; curves: double-Gaussian fit. (**j**) Histogram of suprathreshold orientation-selectivity index (OSI) of 43 orientation selective (OS) neurons. Dashed line: median. (**k**) Scatter plot of subthreshold OSI vs. suprathreshold OSI for 43 OS neurons. Dashed line: reference line with slope = 1.

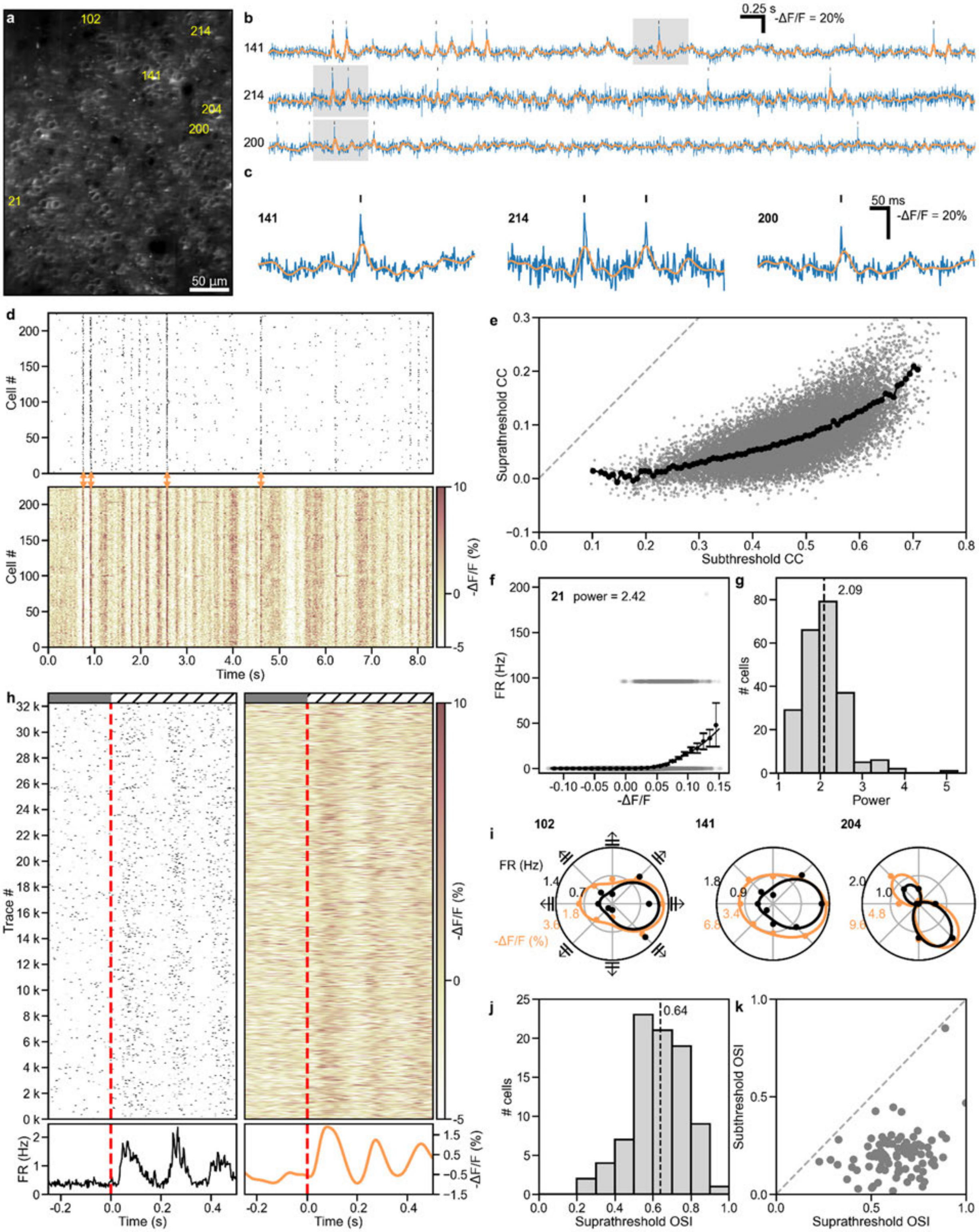

Extended Data Figure 11. Imaging voltage activity from population of neurons in vivo at 2.6 ms/frame. (**a**) JEDI-2P-kv-expressing L2/3 neurons in a 320 μm × 400 μm FOV within a mouse visual cortex imaged at 140 μm depth and 2.6 ms/frame. Pixel size: 0.8 μm × 0.8 μm. 1035 nm excitation at 164 mW post 1.05-NA objective. Numbers: representative neurons. (**b**) Representative -ΔF/F voltage traces (in blue) of numbered neurons in **a**. Black ticks: suprathreshold spikes. Orange traces:

subthreshold -ΔF/F. (**c**) Zoom-in view of voltage traces from the gray boxes in **b**. (**d**) Raster plots of (top) spikes and (bottom) subthreshold -ΔF/F traces of 225 neurons during a representative 8.32-s trial. (**e**) Suprathreshold versus subthreshold pairwise correlation coefficients (CC, gray dots) for 25,200 cell pairs from the 225 neurons in **a**. Black dots: binned averages with 0.01 bin size for subthreshold CC. Dashed gray line: reference line with slope = 1. (**f**) Plot of firing rate (FR) vs subthreshold -ΔF/F for Neuron 20, fit with a power-law function (black curve; power-law exponent = 2.42). Gray dots: FR vs subthreshold -ΔF/F. Black dots: binned average FR with 0.01 bin size in -ΔF/F. Error bar: s.e.m. (**g**) Histogram of fitted power-law exponent values of 225 neurons. Dashed line: median. (**h**) Suprathreshold (left) and subthreshold (right) responses relative to stimulus onset extracted from 32,240 voltage traces of 155 neurons exhibiting visually evoked activity across 26 trials × 8 drifting grating orientations. Top to bottom: raster plot, averaged firing rate (black curve, bottom left panel) and averaged subthreshold -ΔF/F (orange curve, bottom right panel). Red dashed line: onset of drifting grating stimuli. (**i**) Representative polar-plot tuning curves for supra- (black) and subthreshold (orange) voltage responses of Neuron 101, 140, 203, respectively. Dots: measured responses; curves: double-Gaussian fit. (**j**) Histogram of suprathreshold orientation-selectivity index (OSI) of 86 orientation selective (OS) neurons. Dashed line: median. (**k**) Scatter plot of subthreshold OSI vs. suprathreshold OSI for 86 OS neurons. Dashed line: reference line with slope = 1.

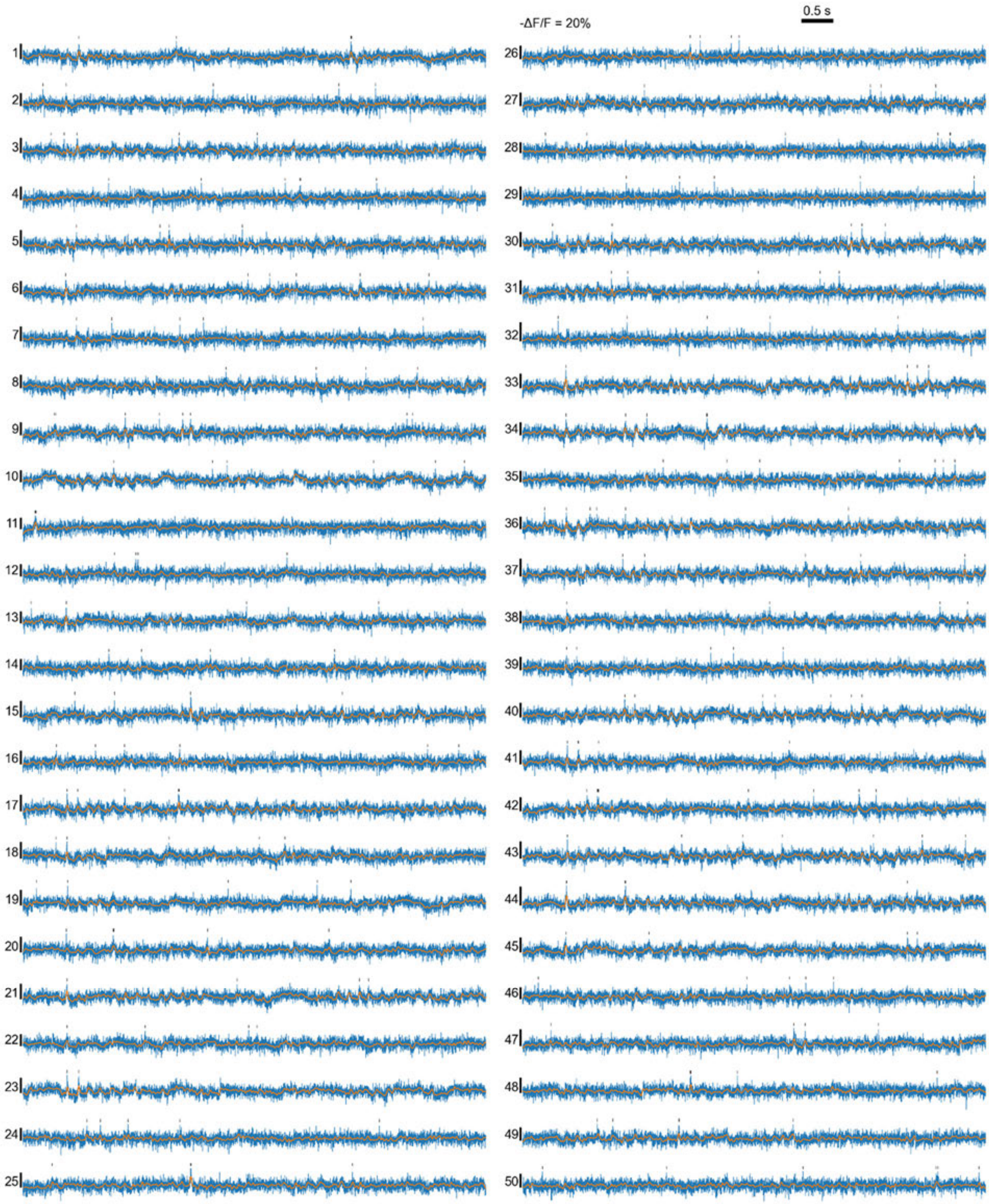
0.5 s
-ΔF/F = 20%
1
2
3
4
5
6
7
8
9
10
11
12
13
14
15
16
17
18
19
20
21
22
23
24
25
26
27
28
29
30
31
32
33
34
35
36
37
38
39
40
41
42
43
44
45
46
47
48
49
50

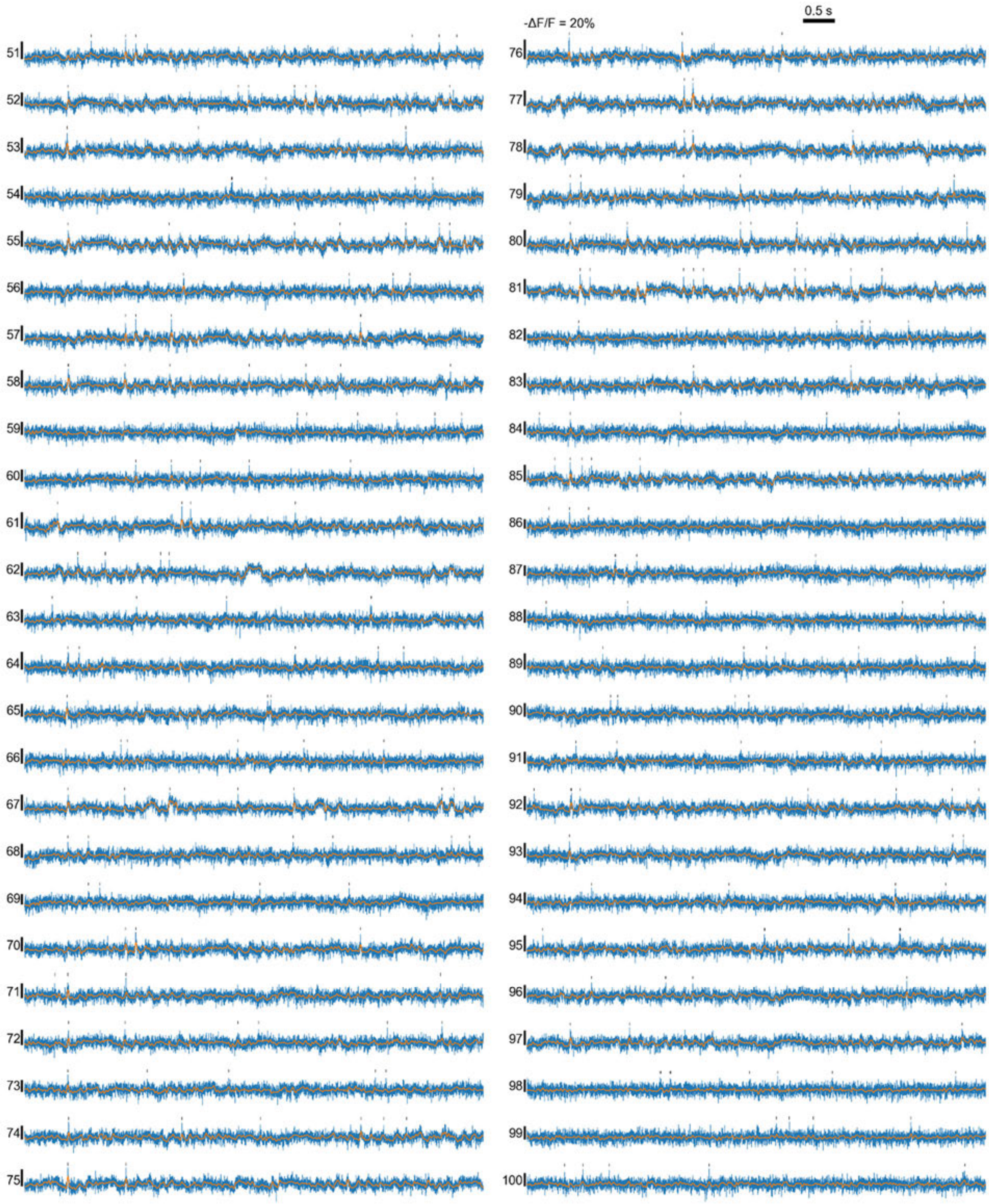

0.5 s
-ΔF/F = 20%
51
52
53
54
55
56
57
58
59
60
61
62
63
64
65
66
67
68
69
70
71
72
73
74
75
76
77
78
79
80
81
82
83
84
85
86
87
88
89
90
91
92
93
94
95
96
97
98
99
100

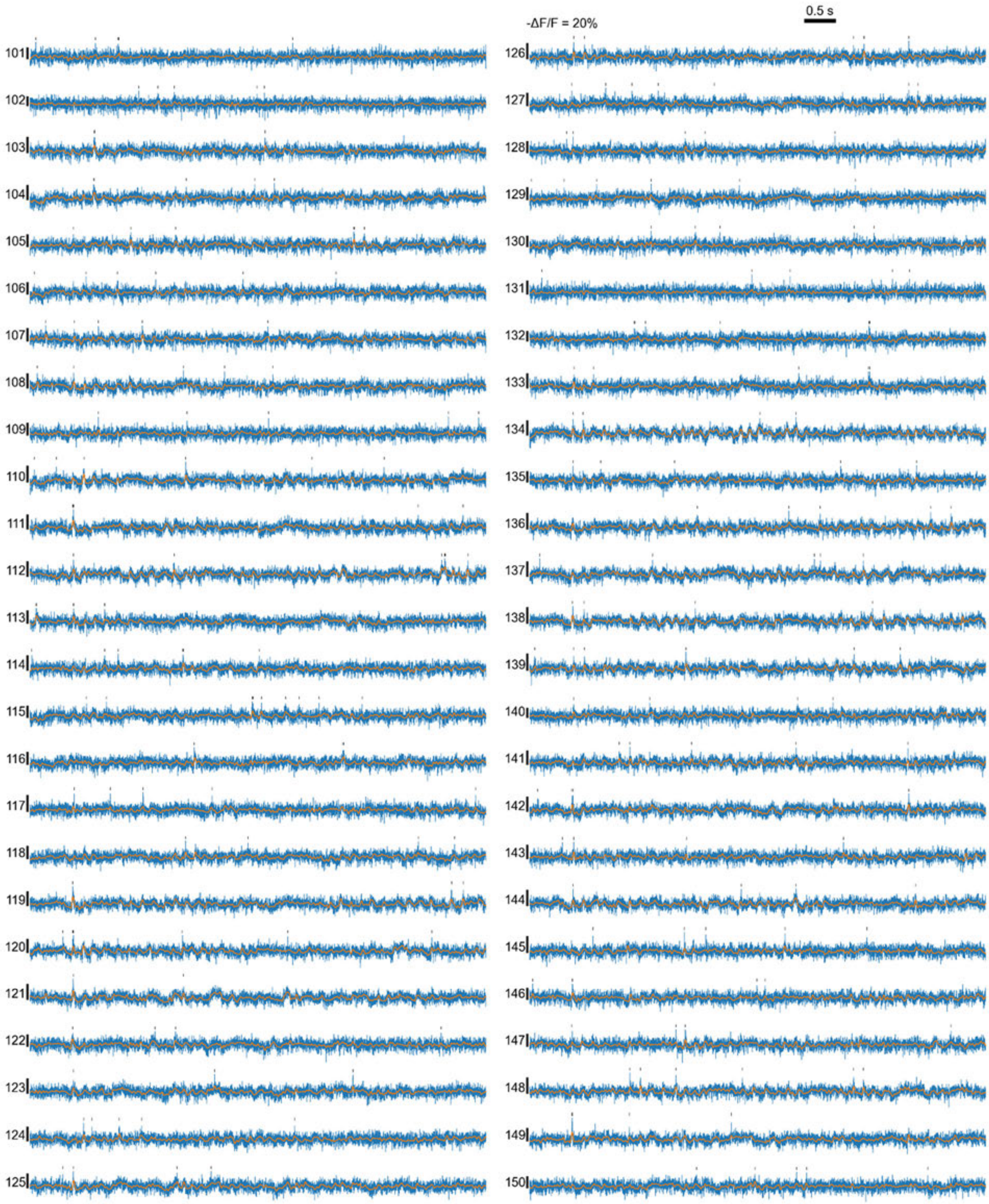

-ΔF/F = 20%
0.5 s
101
102
103
104
105
106
107
108
109
110
111
112
113
114
115
116
117
118
119
120
121
122
123
124
125
126
127
128
129
130
131
132
133
134
135
136
137
138
139
140
141
142
143
144
145
146
147
148
149
150

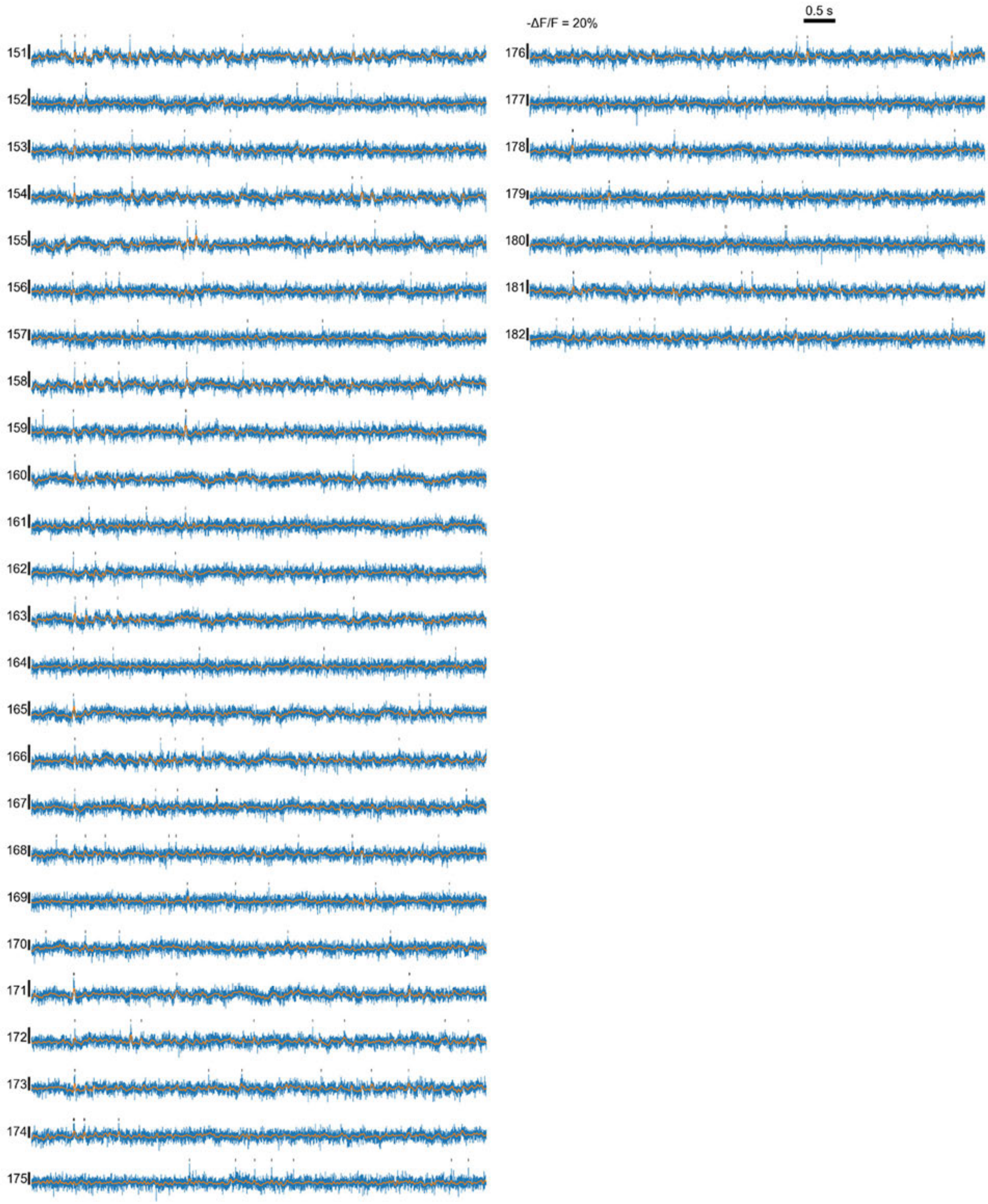

Extended Data Figure 8. Representative -ΔF/F voltage traces (in blue) of all 182 neurons in Fig. 2. Black ticks: suprathreshold spikes. Orange traces: extracted subthreshold -ΔF/F.

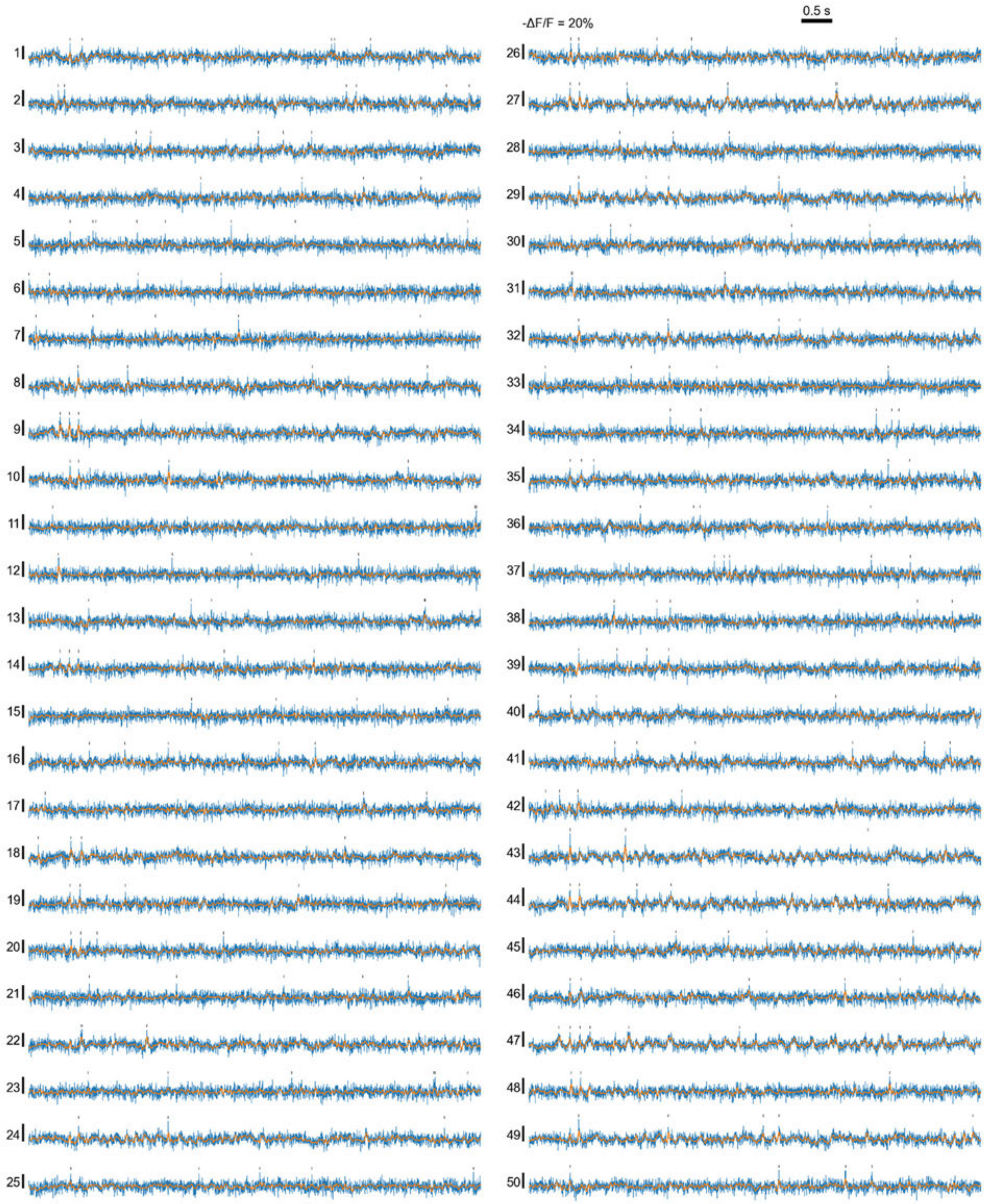
-ΔF/F = 20%
0.5 s
1
2
3
4
5
6
7
8
9
10
11
12
13
14
15
16
17
18
19
20
21
22
23
24
25
26
27
28
29
30
31
32
33
34
35
36
37
38
39
40
41
42
43
44
45
46
47
48
49
50

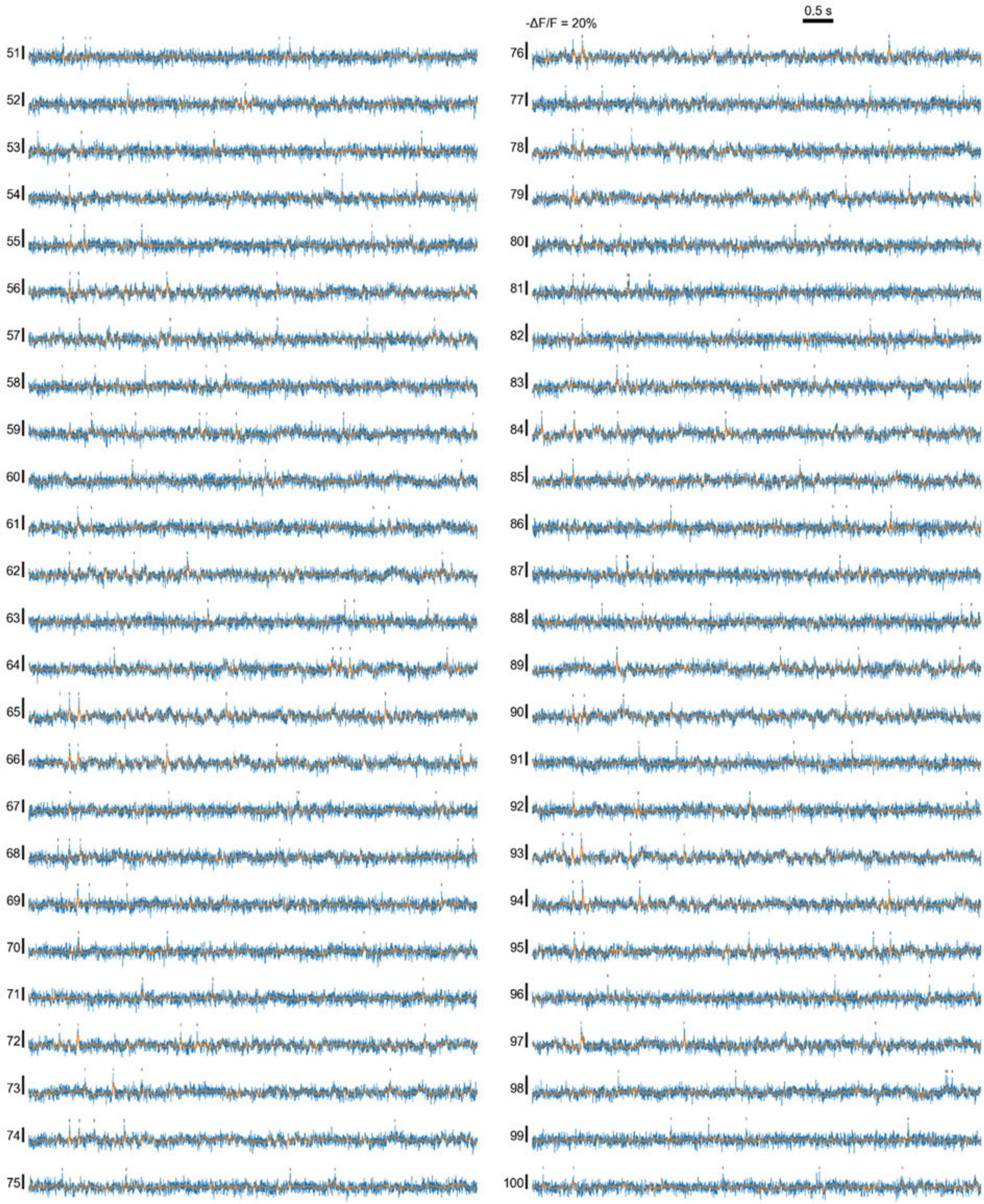
0.5 s
-ΔF/F = 20%
51
52
53
54
55
56
57
58
59
60
61
62
63
64
65
66
67
68
69
70
71
72
73
74
75
76
77
78
79
80
81
82
83
84
85
86
87
88
89
90
91
92
93
94
95
96
97
98
99
100

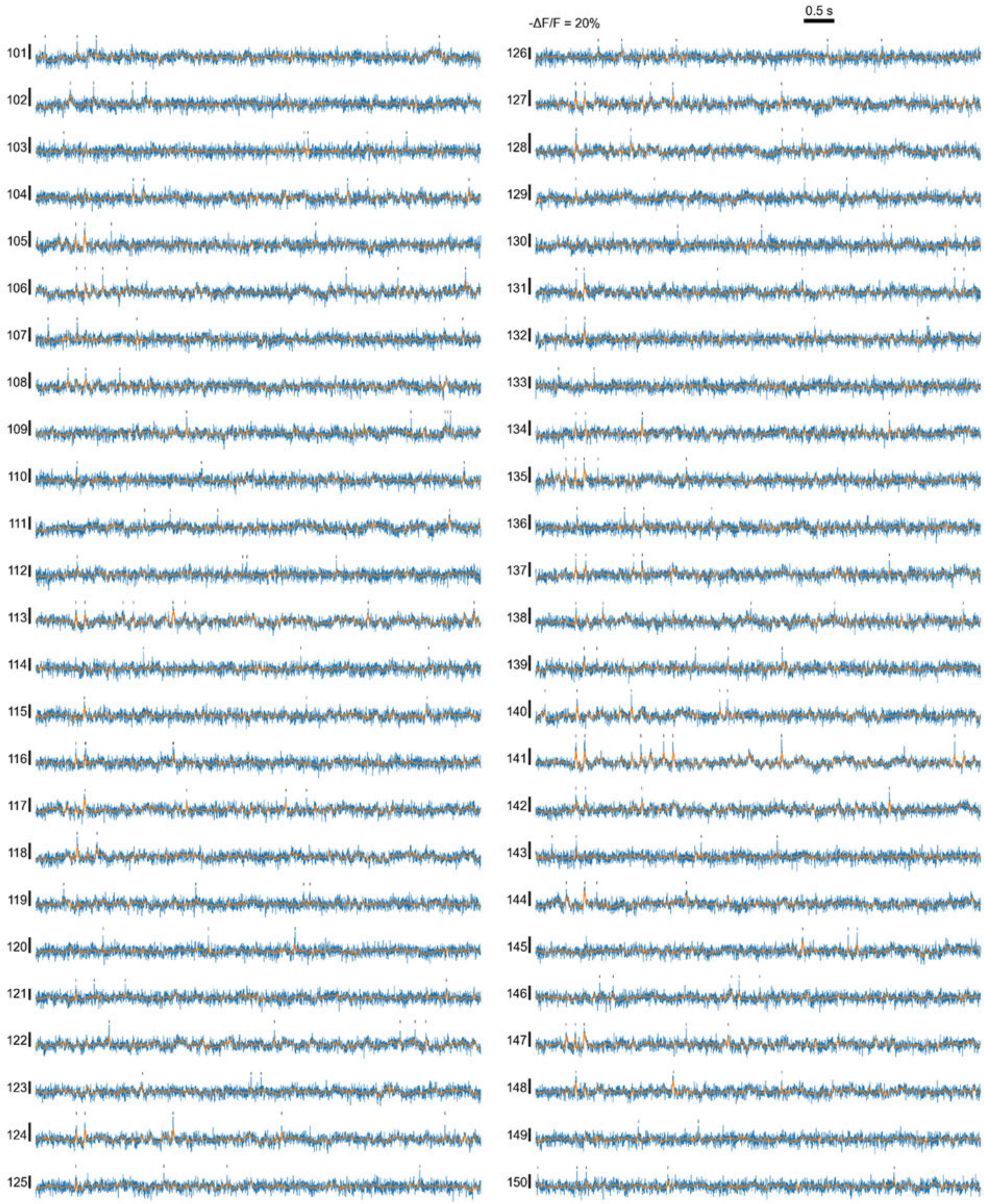

-ΔF/F = 20%
0.5 s
101
102
103
104
105
106
107
108
109
110
111
112
113
114
115
116
117
118
119
120
121
122
123
124
125
126
127
128
129
130
131
132
133
134
135
136
137
138
139
140
141
142
143
144
145
146
147
148
149
150

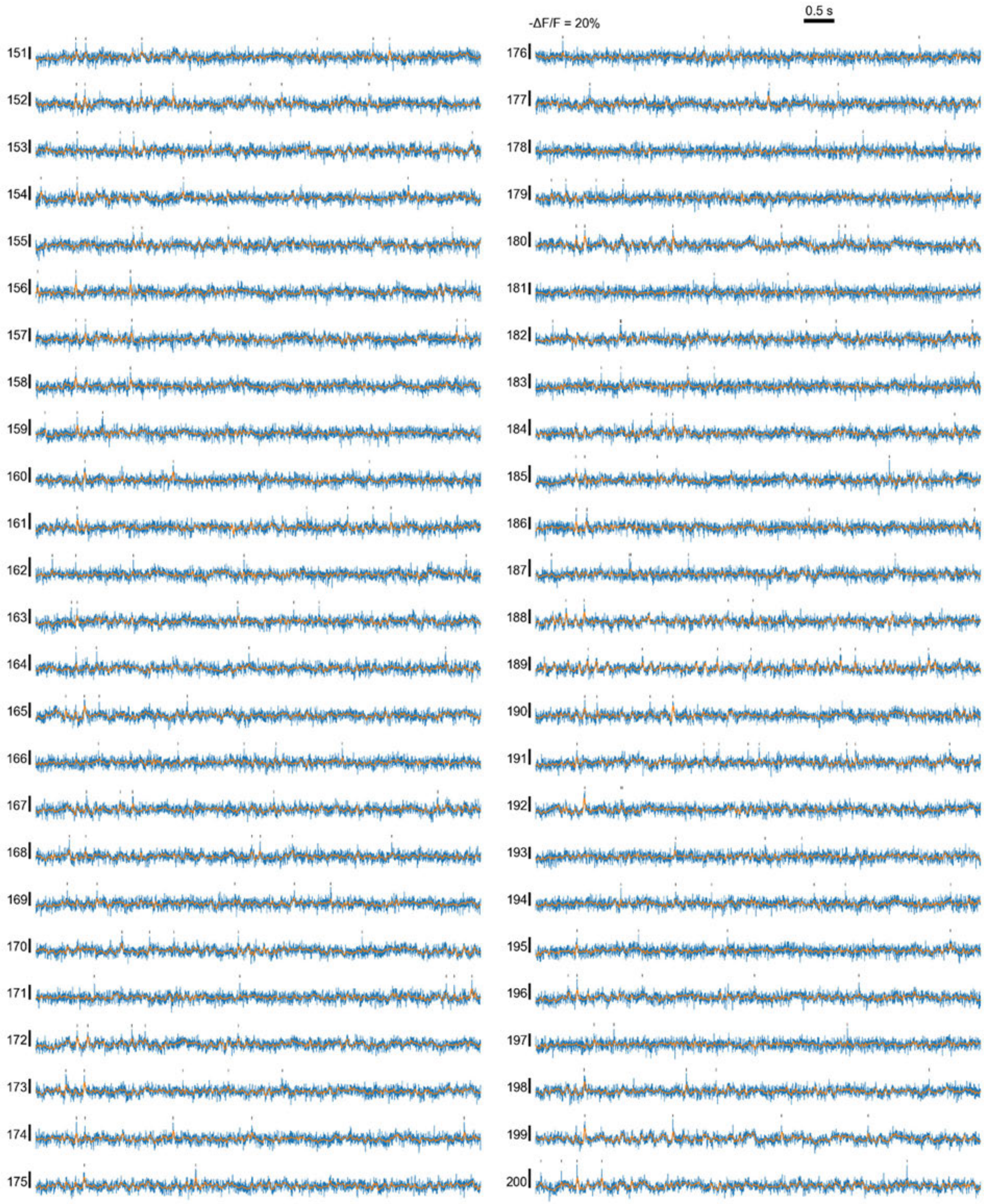
-ΔF/F = 20%
0.5 s
151
152
153
154
155
156
157
158
159
160
161
162
163
164
165
166
167
168
169
170
171
172
173
174
175
176
177
178
179
180
181
182
183
184
185
186
187
188
189
190
191
192
193
194
195
196
197
198
199
200

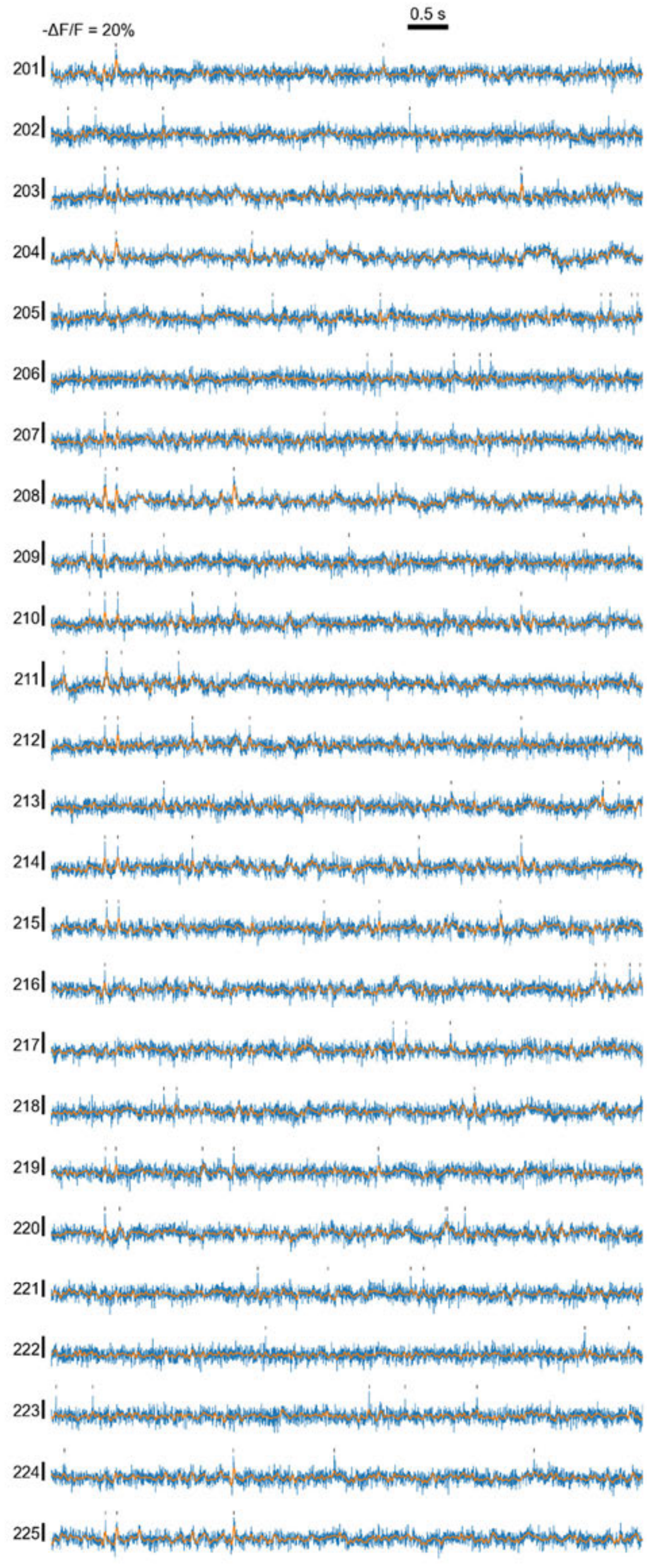

Extended Data Figure 12. Representative -ΔF/F voltage traces (in blue) of all 225 neurons in Extended Data Fig. 11. Black ticks: suprathreshold spikes. Orange traces: subthreshold -ΔF/F.

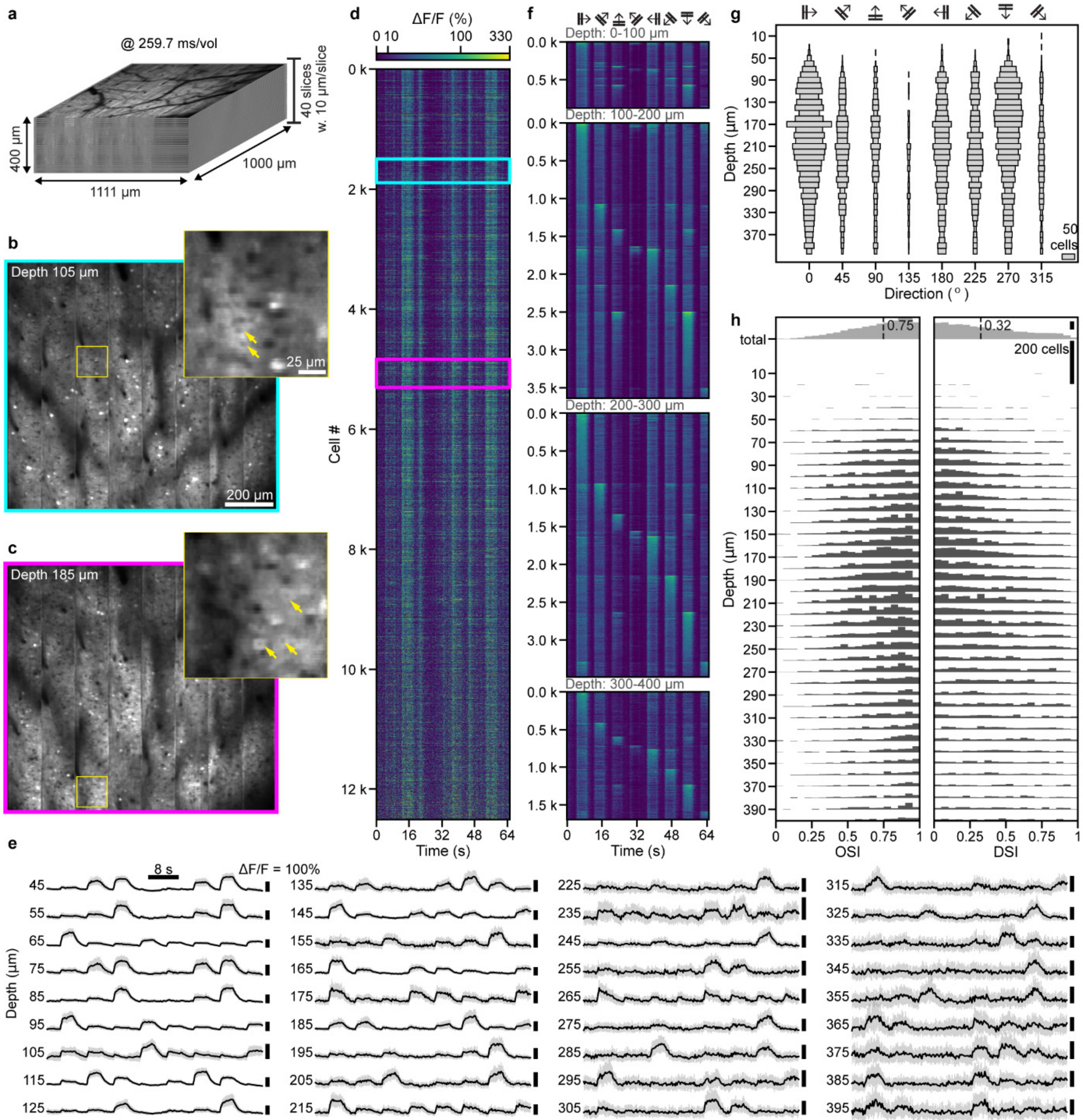

Figure 3. Large-scale in vivo volumetric imaging of calcium activity. (**a**) An 1,111 µm × 1,000 µm × 400 µm volume in the visual cortex of a Slc17a7-IRES2-Cre × Ai162D mouse imaged at 260 ms/vol. Voxel size: 1.4 µm × 2 µm × 10 µm. 920 nm excitation at 102 mW post 0.8-NA objective. (**b**,**c**) Example images at 105 µm and 185 µm depths. Insets: zoomed-in views of areas in yellow boxes. Arrows: neurons showing nucleus-excluded GCaMP6s expression. (**d**) Simultaneously recorded ΔF/F calcium traces from 12,511 neurons during a 64-s trial with drifting grating visual stimulation. Cyan and magenta boxes: calcium traces from neurons in b and c, respectively. (**e**) Example trial-averaged ΔF/F traces of neurons in **a** at 45 to 395 µm below dura. Gray shade: standard deviation. (**f**) 36-trial-averaged calcium traces from 9,811 neurons with visually evoked activity, grouped by depth 0-100 µm, 100-200 µm, 200-300 µm, and 300-400 µm. (**g**) Horizontal bars with their lengths indicating the numbers of neurons with their maximal ΔF/F at a specific grating direction for all 9,767 OS neurons across depths. (**h**) Histograms showing the OSI (left) and direction-selectivity index (DSI, right) distributions of OS neurons at each depth. Gray histogram (top): OSI and DSI distributions for all neurons. Dashed lines: median values.

2) Quantitative evaluation of signal-to-noise ratio (SNR) should be performed as a function of the imaging conditions (e.g., SNR vs photon/pixel). This is particularly important for sub-threshold responses of genetically encoded voltage indicators, which have very small amplitude (close to noise level). As the authors themselves underline, compared to electrophysiological approaches one key advantage of fast imaging approaches applied to fluorescence voltage indicators is the possibility to measure at the same time the sub- and supra-threshold response in a large number of cells (in this manuscript about 200 neurons) with cellular resolution. However, this advantage can be exploited only if the SNR of sub- and supra-threshold responses is high enough.

We appreciate the reviewer's suggestions and have incorporated SNR analysis for spikes and spike detection in new Supplementary Note 2 and Supplementary Figs. 3-5. In Supplementary Table 4, we provided brightness for all images contained in the manuscript. In Supplementary Table 5, we provided photon count statistics for both ROIs and individual pixels for activity imaging data. In Supplementary Table 6, we provided the noise level for spike detection. See Pages 33-37 of this document and Supplementary Note 2 for additional discussions on SNR.

Regarding the concern whether our SNR is sufficient for detecting subthreshold activity: Because subthreshold activity at the cell body lasts much longer than action potentials, we used a 20-Hz 5th-order Butterworth filter to low-pass-filter the -ΔF/F traces and extract subthreshold activity. Because of the filtering process, it is difficult to estimate the SNR of the subthreshold traces. However, it can be seen from our data that the extracted subthreshold activity traces (orange traces below) are of good quality:

The extracted subthreshold activity (orange traces) in Fig. 2 (1.25 ms/frame):

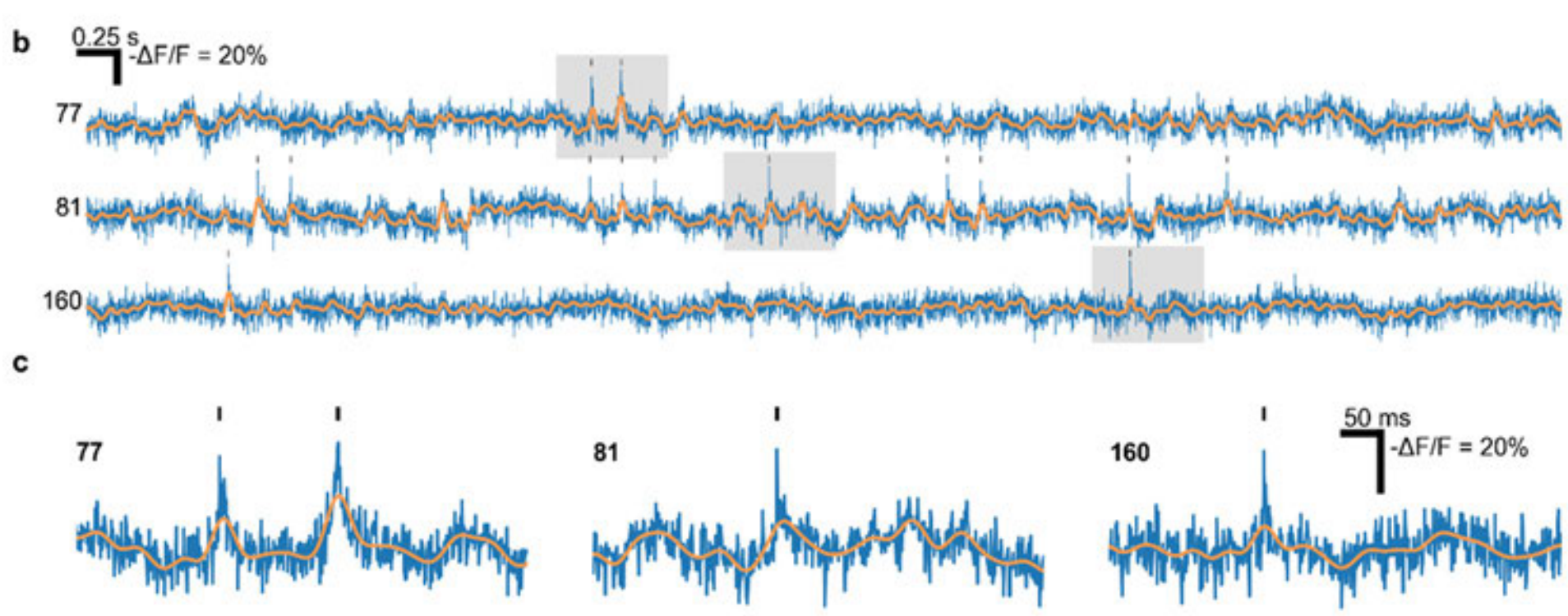

The extracted subthreshold activity (orange traces) in Extended Data Fig. 11 (2.6 ms/frame):

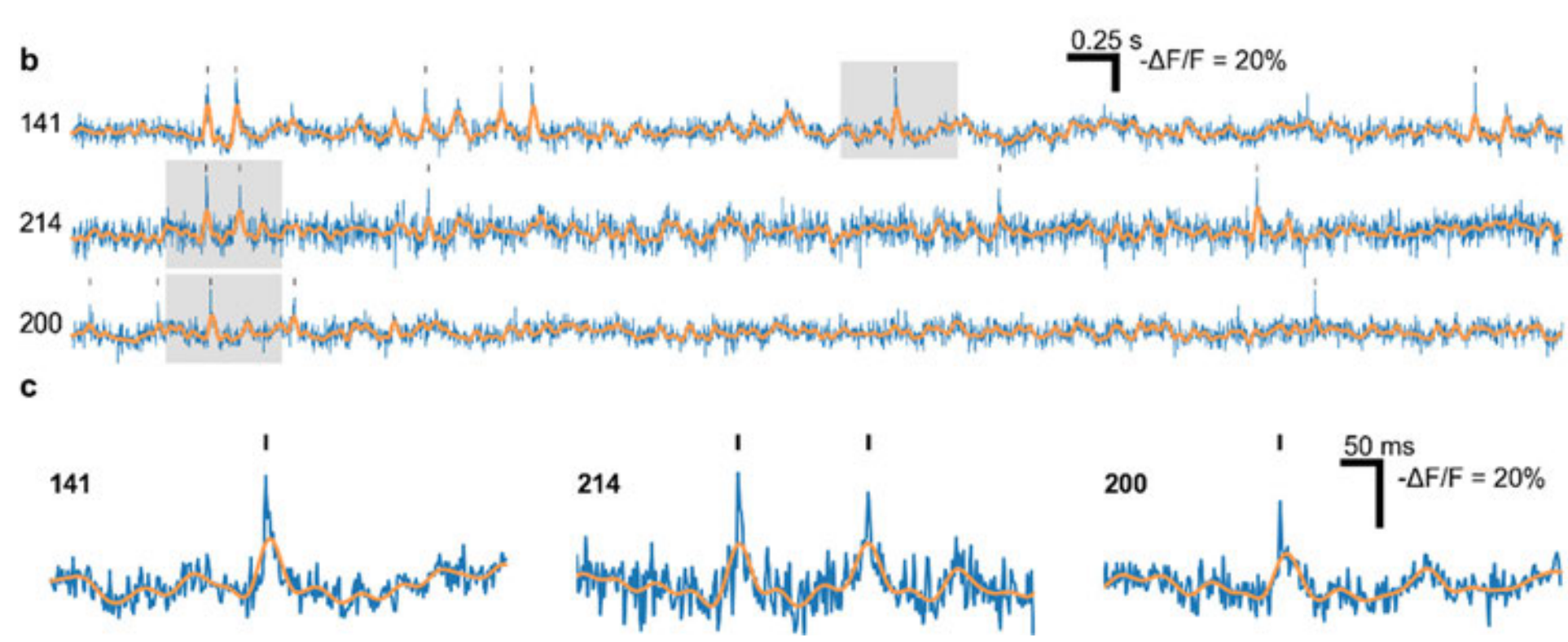

Such filtering approaches have been widely used in previous publications (e.g., https://doi.org/10.1038/s41592-024-02275-w, https://doi.org/10.1126/science.abg0501). Previous studies have also shown that frame rates of tens of Hz were adequate for capturing subthreshold activity (e.g., https://www.nature.com/articles/s41467-021-27444-9#Sec8, https://doi.org/10.1126/science.abg0501).

3) At the same laser repetition rate, FACED 2.0 enables acquiring 20% larger pixel/s values compared to FACED 1.0 (1x10^8 pixels/s vs 0.8x10^8 pixels/s), yet the authors are here able to record from much larger number of neurons compared to the number of neurons recorded in their previous publication (Wu et al. 2020). It thus appears important to quantify and discuss how much higher performance in imaging the large-scale population of neurons presented in the current study was achieved sacrificing SNR vs improving the optical method.

Please see our detailed response to Comment 2 of the other reviewer (Pages 33-37 of this document), which shows that the increase in neuron numbers does not arise from sacrificing SNR. Instead, the improved voltage imaging performance comes from three factors detailed below:

1. The improvement in performance of JEDI-2P over ASAP3. As described in Fig. 2 of the JEDI-2P paper (https://doi.org/10.1016/j.cell.2022.07.013, panels reproduced below), JEDI-2P has larger -ΔF/F for spikes (2× folds, panel E) and higher brightness (1.7×, panel I) under 2P excitation than ASAP3. Together, these factors allow us to sample the somata of JEDI-2P-expressing neurons with fewer pixels (pixel size: 0.8 μm × 0.8 μm) than for ASAP3-expressing neurons (pixel size: 0.625 μm × 0.27 μm), enabling a larger FOV and higher number of neurons.

[redacted]

2. JEDI-2P mice used in current manuscript had higher labeling density than the ASAP3 mice used in our previous work. This allowed us to collect data from more neurons from the same-sized imaging area. See on the right a side-by-side comparison of 50 μm × 250 μm FOV of our previous publication (left: cropped from Supplementary Fig. 11a of Wu et al.) and this work (right: cropped from Fig. 2a).
3. A complete rewrite of the microscope control software for FACED 2.0 allowed us to increase the Y galvo scanning rate from 500 Hz (i.e., 1 kHz frame rate in Wu et al) over a 250 μm range to 833 Hz (1.67 kHz frame rate) over a 585 μm range for the 25× 1.05-NA objective. Adding an additional X galvo that scans along the FACED line scan direction in FACED 2.0 allowed us to tile FACED FOVs to further enlarge the area that can be scanned at high speed.

[redacted]

Together, the three factors above increased the number of neurons that we can simultaneously collect voltage imaging data from.

For blood vessel and calcium imaging data, we used a 16× NA-0.8 objective, whose longer focal length (12.5 mm) enables us to cover a 3× larger area of 140 μm × 1,035 μm in each FACED frame than with the 25× NA-1.05 objective (focal length of 7.2 mm). Together with the slower frame rates required for calcium imaging and the newly implemented ability of z-scanning in FACED 2.0, this greatly improved the number of neurons that we could record calcium signal from.

4) Results section: "the continual Z movement during image acquisition did not generate any in-frame motion artifacts". This should be quantified and demonstrated.

We thank the reviewer for suggesting this additional analysis. To support this statement, we compared 3D image stacks of static fluorescent fiber structures acquired at different rates (Supplementary Fig. 1,2).

For ground-truth measurements, we stepped and stopped the objective piezo at a Z position and acquired 100 XY images of 1,110 μm × 1,000 μm FOVs at 5.3 ms/frame; we repeated this at 40 Z positions with 10 μm Z-step size (Supplementary Fig. 1) or at 52 Z positions with 15 μm Z-step size (Supplementary Fig. 2) to cover the same volumes as in Fig. 3 and Extended Data Fig. 16, respectively. The averages of the 100 XY images at each Z position are used as the ground-truth volumetric datasets (left panels, Supplementary Figs. 1, 2).

We then imaged the same structures by continuously moving the objective in Z to achieve the same volumetric rates as in Fig. 3 (259.7 ms/volume by scanning the objective piezo continuously at 1,887 μm /s) and Extended Data Fig.16 (365.7 ms/volume by scanning the objective piezo continuously at 2,830 μm/s). We acquired 100 volumes and compared the volume-averaged XY images at the same depths (right panels, Supplementary Figs. 1, 2) with those from the ground-truth datasets.

Visual inspection indicates that the image stacks acquired by these two methods are highly similar. To quantify the similarity between the two stacks, we calculated the normalized cross-correlation coefficients (ρ_{XY}), which measures how well the images match, with higher values indicating greater similarity:

$$\rho_{XY} = \frac{\frac{1}{NML}\sum_{i=1}^{N}\sum_{j=1}^{M}\sum_{k=1}^{L}(X_{ijk}-\mu_X)(Y_{ijk}-\mu_Y)}{\sigma_X\sigma_Y}$$

Here, X_{ijk} and Y_{ijk} are the pixel values at position (i, j, k) in the volumetric (X) and ground-truth stacks (Y), respectively. N, M, and L represent the number of pixels along the X, Y, and Z axes. μ_X and μ_Y are the means, and σ_X and σ_Y are the standard deviations of pixel values within the entire image stacks:

$$\mu_X = \frac{1}{NML}\sum_{i=1}^{N}\sum_{j=1}^{M}\sum_{k=1}^{L}(X_{ijk}),\ \mu_Y = \frac{1}{NML}\sum_{i=1}^{N}\sum_{j=1}^{M}\sum_{k=1}^{L}(Y_{ijk})$$

$$\sigma_X = \sqrt{\frac{1}{NML}\sum_{i=1}^{N}\sum_{j=1}^{M}\sum_{k=1}^{L}(X_{ijk}-\mu_X)^2},\ \sigma_Y = \sqrt{\frac{1}{NML}\sum_{i=1}^{N}\sum_{j=1}^{M}\sum_{k=1}^{L}(Y_{ijk}-\mu_Y)^2}$$

We found $\rho_{XY} = 98\%$ for data in Supplementary Fig. 1 and $\rho_{XY} = 95\%$ for data in Supplementary Fig. 2, demonstrating a high level of agreement between the stack acquired through continuous Z scanning and the ground-truth stack acquired through step-and-stop Z scanning.

These figures and analyses are now included as Supplementary Figs. 1,2 (reproduced below) and Supplementary Note 1.

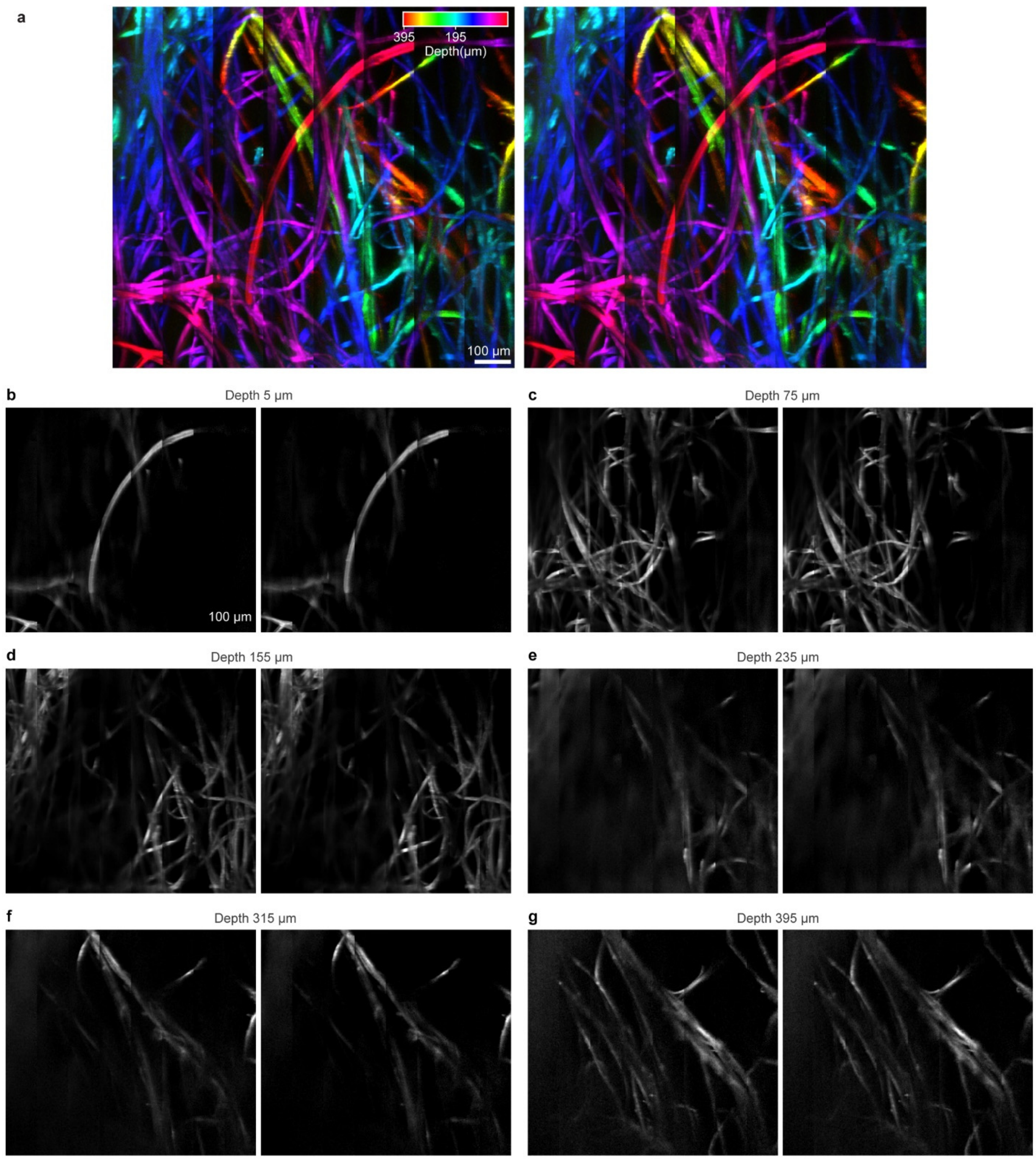

Supplementary Figure 1. FACED volumetric imaging accurately captures 3D structures in a 1,111 µm × 1,000 µm × 400 µm volume at 259.7 ms/vol without in-frame motion artifacts. Comparison of ground-truth (left, acquired by keeping the objective lens stationary at each depth) and volumetric (right, acquired by continuous Z scanning) images of fluorescent fibers. **(a)** Maximum intensity projection of 3D fiber structures (color-coded by depth) across the entire imaging volume for both ground-truth (left) and volumetric (right) datasets. **(b-g)** Representative ground-truth (left) and volumetric (right) images at depths of 5 µm, 75 µm, 155 µm, 235 µm, 315 µm, and 395 µm. Voxel size: 1.4 µm × 2 µm × 10 µm. 1035 nm excitation at 21 mW post 16× 0.8-NA objective.

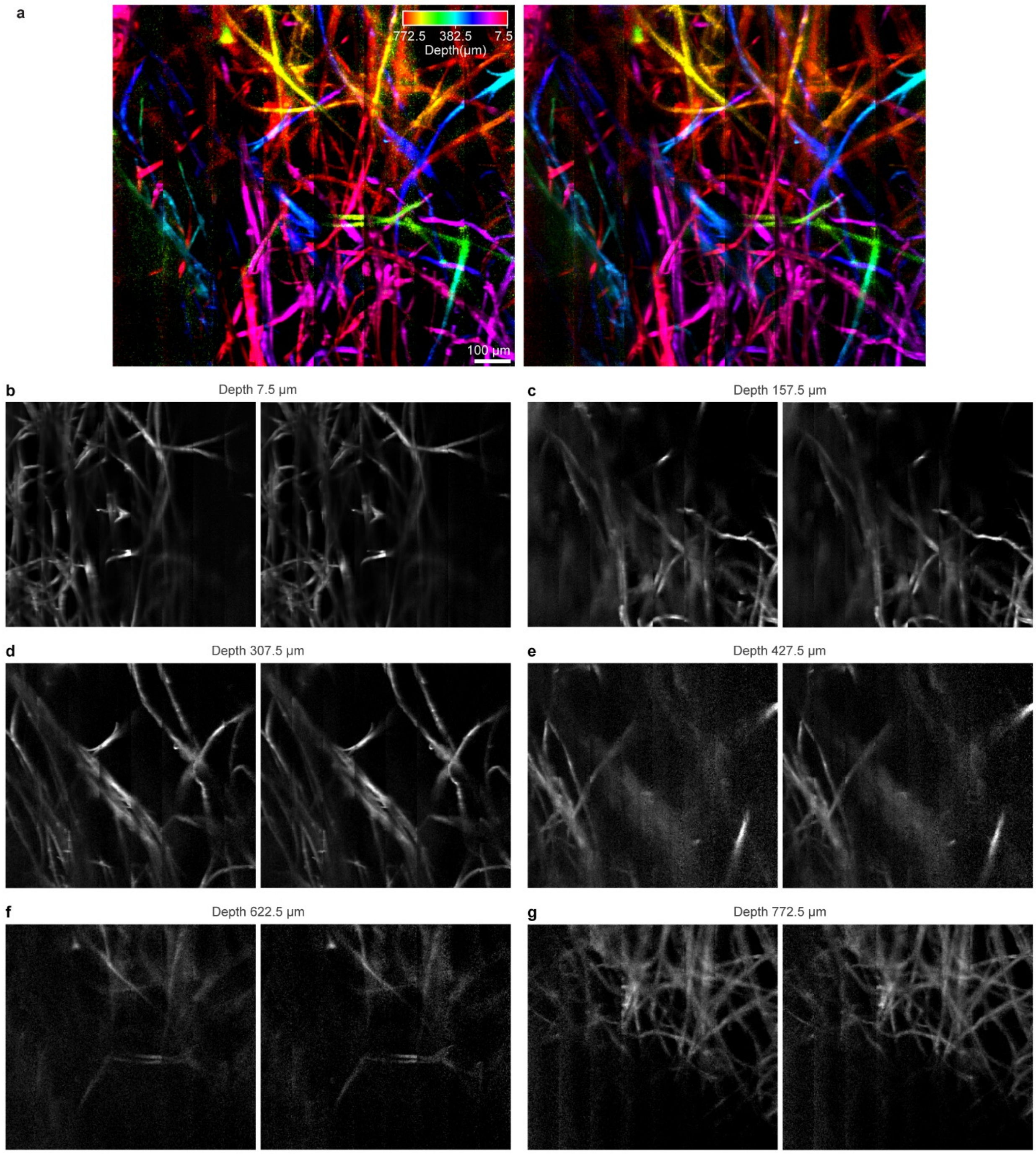

Supplementary Figure 2. FACED volumetric imaging accurately captures 3D structures in a 1,111 μm × 1,000 μm × 780 μm volume at 365.7 ms/vol without in-frame motion artifacts. Comparison of ground-truth (left, acquired by keeping the objective lens stationary at each depth.) and volumetric (right, acquired by continuous Z scanning) images of fluorescent fibers. **(a)** Maximum intensity projection of 3D fiber structures (color-coded by depth) across the entire imaging volume for both ground-truth (left) and volumetric (right) datasets. **(b-g)** Representative ground truth (left) and volumetric (right) images at depths of 7.5 μm, 157.5 μm, 307.5 μm, 427.5 μm, 622.5 μm, and 772.5 μm. Voxel size: 1.4 μm × 2 μm × 15 μm. 1035 nm excitation at 21 mW post 16× 0.8-NA objective.

5) Results, voltage indicator imaging section: it is stated that 4% photo-bleaching was observed during each 8s trial and that an overall photo-bleaching of 9% was observed across the entire 296 s-long recording. Please provide a graph to show the photo-bleaching amplitude as a function of trial number within the recording session.

Thank you for the suggestion. we now include Extended Data Fig. 9 (reproduced below) on the voltage imaging datasets in Fig. 2 and Extended Data Fig. 11.

We computed the fluorescence signal of each frame as the mean pixel value of the frame. In Extended Data Fig. 9b,d, we plotted the time trace of the fluorescence signal during 27 (for panel b) or 26 (for panel d) visual stimulation trials (each trial included 8 blank and 8 drifting-grating visual stimuli). Here the florescence signal traces were low-pass filtered using a 1/3 Hz, 3rd-order Butterworth filter to better visualize the photobleaching dynamics. In Extended Data Fig. 9a,c, we further plotted the time-averages of the fluorescence signals of each trial (8.25 s for Extended Data Fig. 9a and 8.32 s for Extended Data Fig. 9c).

For the JEDI-2P data in Fig. 2 (Extended Data Fig. 9a,b), we observed 15% photobleaching over the entire 222.75-s acquisition duration, and an average of 4% photobleaching within an 8.25-s-long trial. For the JEDI-2P data in Extended Data Fig. 11 (Extended Data Fig. 9c,d), we observed 10% photobleaching over the entire 216.32-s acquisition duration, and an average of 5% photobleaching within an 8.32-s-long trial. Note that JEDI-2P showed partial recovery of brightness between trials (Extended Data Fig. 9b,d), a phenomenon that we also observed in our previous FACED study with ASAP3 (Wu et al., Supplementary Figure 9).

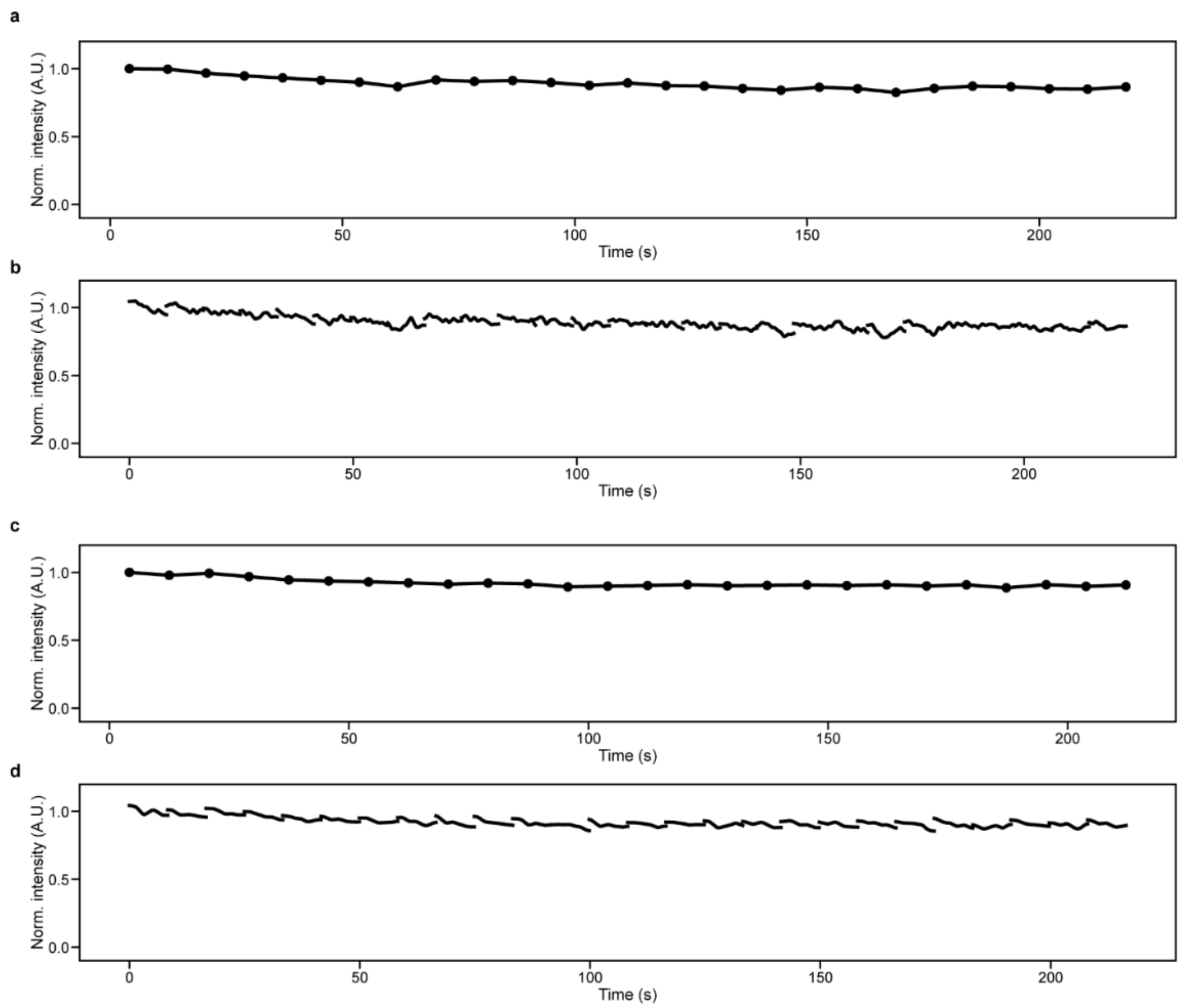

Extended Data Figure 9. Photobleaching of JEDI-2P-kv fluorescence during FACED 2PFM imaging. (a) Time-averaged fluorescence signal over each 8.25-s continuous imaging trial across 27 trials. Same data as Fig. 2, 160 µm × 400 µm FOV acquired at 1.25 ms/frame for a total duration of 222.75 s using 1035 nm excitation at 163 mW post-objective from a depth of 132 µm below dura. A 15% photobleaching observed over the entire 222.75-s acquisition duration. (b) Fluorescence signal over each 8.25-s continuous imaging trial across 27 trials. Same data as (a) and an average of 4% photobleaching observed within each 8.25-s-long trial. (c) Time-averaged fluorescence signal over each 8.32-s continuous imaging trial across 26 trials. Same data as Extended Data Fig. 11, 320 µm × 400 µm FOV acquired at 2.6 ms/frame for a total duration of 216.32 s using 1035 nm excitation at 164 mW post-objective from a depth of 140 µm below dura. A 10% photobleaching over the entire 216.32-s acquisition duration. (d) Fluorescence signal over each 8.32-s continuous imaging trial across 26 trials. Same data as (c) and an average of 5% photobleaching observed within an 8.32-s-long trial. Pixel size for all data: 0.8 µm × 0.8 µm.

6) Any sign of photo-damage introduced by FACED 2.0?

No evidence of photodamage was observed in the datasets included in this manuscript. However, in mice with compromised brain tissue health (indicated by dense autofluorescent punctates), when imaging superficial brain layers (depth < 100 µm) under high excitation power (>220 mW) and high numerical aperture (1.05 NA), we occasionally observed photodamage in the form of growing autofluorescent blob-like structures. No data with photo-damage was included in the manuscript.

7) What is the group velocity dispersion of laser pulses in FACED 2.0? Was the laser beam pre-compensated? If I am not mistaken, in FACED 1.0 pre-chirping was used, but I could not find clear indication about pre-compensation in this study.

The estimated group delay dispersion (GDD) for the 920 nm and 1035 nm light paths was 11445 fs^2 and 11257 fs^2, respectively. For FACED 2.0, the laser beam was not pre-compensated with a dispersion compensator for either 1035 nm or 920 nm excitation.

For the 1035 nm excitation, the pulse width of the excitation light (i.e., output from the Monaco laser) is ~300 fs. The amount of GDD would only elongate a 300-fs-long pulse to 318 fs. Therefore, dispersion compensation would not substantially enhance signal.

For the 920 nm excitation, the output from our OPA system at 920 nm was around 1.8 W and had ~167 fs pulse width. For 167 fs pulses, the amount of GDD present would elongate the pulse to 253 fs. Fully compensating the GDD of the pulse would lead to <50% increase in signal brightness. However, our dispersion compensator (used for FACED 1.0) has degraded coatings and currently a power throughput of 50%. With this dispersion compensator, the system power throughput would drop from 9.3% to 4.6%, limiting the post objective excitation power to 84 mW (from 168 mW without dispersion compensation). Utilizing the dispersion compensation would thus lead to a reduction in signal strength. As a result, we decided not to employ pre-compensation for our excitation light.

8) The system needs to be aligned every 5-30 minutes. This is quite a wide range of short durations, which may partially overlap with the duration of recording sessions (e.g., the 296 s-long recording presented in this study). Is there a way to monitor the quality of the alignment during the recording session?

Large temperature fluctuations in our lab (±3 °F) cause drift in the light path. We continuously monitored the temporal profile of the FACED foci using a fast photodiode and adjusted alignment of the FACED mirror pairs using piezo actuators throughout the experiment. Datasets were discarded if large drift occurred during imaging. We are developing an active beam stabilization system to eliminate the need for frequent alignment.

9) The manuscript would greatly gain by the addition of a supplementary text critically describing the improvement of FACED 2.0 compared to FACED 1.0, while referring to Suppl. Table 1.

We appreciate the reviewer's suggestion. We added Supplementary Note 5 (reproduced below) to compare FACED 2.0 with FACED 1.0:

"FACED 2.0 substantially enhances the imaging capabilities of FACED 2PFM by offering larger imaging FOVs and longer continuous imaging durations than FACED 1.0, in addition to having volumetric imaging capability. These improvements, achieved via both hardware and software optimization, allow for selecting imaging area/volume/throughput based on the temporal dynamics of biological events, enabling the study of larger neuronal populations over wider spatial domains and longer timescales at higher throughput than FACED 1.0.

In FACED 1.0, the FACED module was configured to generate linescans containing 80 pixels at a 2 ns inter-pixel delay, with its imaging FOV restricted to 50 µm × 250 µm (25× 1.05 NA objective). In FACED 2.0, the FACED module is configured to generate linescans containing 100 pixels at a 1 ns inter-pixel delay, with a single FACED FOV reaching 80 µm × 585 µm (25× 1.05 NA objective) or 140 µm × 1035 µm (16× 0.8 NA objective).

A complete rewrite of the microscope control software for FACED 2.0 allows us to increase Y galvo scanning to 833 Hz (1.67 kHz frame rate) over 585 µm for 25× 1.05-NA objective and 1,035 µm for 16×

0.8 NA objective, a substantial improvement over from FACED 1.0 with 500 Hz Y galvo scanning over 250 µm for 25× 1.05-NA objective. Adding an additional X galvo that scans along the FACED linescan direction in FACED 2.0 allows tiling FACED FOVs to further enlarge the area that can be scanned at high speed. For example, using the 16× 0.8 NA objective, an area of 1111 µm × 1000 µm can be scanned at 192 Hz.

The control software also synchronizes 2D FACED imaging with continuous axial scanning of the microscope objective, enabling volumetric imaging. The large FOV and high frame rate of FACED 2.0 enable calcium activity of >10,000 neurons to be collected over large volumes at true cellular resolution (e.g., 1111 µm × 1000 µm × 780 µm at 365.7 ms/volume).

FACED 1.0 utilized a PXIe-5160 digitizer (National Instruments), which limits the maximal continuous imaging duration to 6 s. FACED 2.0 uses a high-speed digitizer with onboard FPGA (ADQ7-DC, Teledyne SP Devices). Together with a custom multi-thread data acquisition program that optimizes data transfer and storage, it enables continuous data streaming, limited in its duration only by disk storage size."

10) The software for data acquisition and system control is not shared.

We have updated our GitHub repository (URL: https://github.com/JiLabUCBerkeley/FACED2PFM2p0-analysis) to include the latest software for data acquisition and system control.

Minor comments:

1) In the methods, please add description of PSF measurements, including details about average laser power across conditions.

We added a description of PSF measurements to **Point spread function (PSF) measurements** section in **Methods** (reproduced below):

"**Point spread function (PSF) measurements**

PSF measurements (**Extended Data Figs. 2,3**) were performed using 0.2-µm-diameter red fluorescent beads (Invitrogen™ F8786) positioned in the focal plane of the microscope objective. Using the galvo mirrors and the objective piezo stage, we moved the FACED focal array relative to the fluorescent beads in 3D and recorded the fluorescence signal at each position. For the 25× 1.05 NA objective, step sizes of 0.1 µm in X, 0.05 µm in Y, and 0.2 µm in Z were utilized. For the 16× 0.8 NA objective, step sizes of 0.15 µm in X, 0.05 µm in Y, and 0.5 µm in Z were used. We then measured the full widths at half maximum (FWHM) of the PSF profiles excited by each FACED focus along the X, Y, and Z axes. The same process was repeated for beads located along the horizontal, vertical, and the two diagonal directions of the entire FOV."

2) Please add dispersion quantification (e.g., standard deviation) to black dots in Fig. 2e.

We appreciate the reviewer's suggestion. We have incorporated standard errors of the mean (s.e.m.) as error bars on the black dots in panel e (now panel f) of the updated Fig. 2 and the additional Extended Data Fig. 11, following Priebe et al. Nat Neurosci 7, 1113–1122 (2004).

3) Is laser power modulated during volumetric imaging? Please specify in methods.

The laser power stayed constant and was not modulated during the volumetric imaging. We now make this point explicit in **FACED 2.0** section of **Methods**.

4) Methods section: with regard to the formula "vn+1=vn + an". "vn" indicates velocity and "an" indicates acceleration. The term "an" should be multiplied by "t" (time) to be in the same unit (m/s) as velocity. Please amend.

We thank the reviewer for bringing this to our attention. We have updated in the relevant section of the manuscript.

5) Suppl. Fig. 1: the red and pink colors are difficult to distinguish. Please consider changing the color code.

We thank the reviewer for the suggestion and have replaced the original color coding in these figures (now Extended Data Figures 2 and 3).

6) Please provide an explanation for the trend of the M and H traces to grow asymmetrically from negative values of the position to positive values (Supp. Fig. 2n and Suppl. Fig. 3n), while the opposite trend for L, H, and M traces is observed in Suppl. Fig. 2s and Suppl. Fig. 3s.

Panel n and panel s characterized the PSFs at different locations within the FOV. As we referred to in the main text: "Even though field-dependent aberrations in the imaging setup caused modest variations in resolution (Extended Data Figs. 2,3)", the current microscope has residual aberrations within the optical system that vary across the FOV. Coupled with the varying effective NA of each excitation focus within the FACED focal array, they led to the spatially varying PSF profile as reported here. In an ongoing project, we corrected optical system aberration at the central FOV, leading to a more symmetric PSF distribution (see the spatial distribution of axial PSF below):

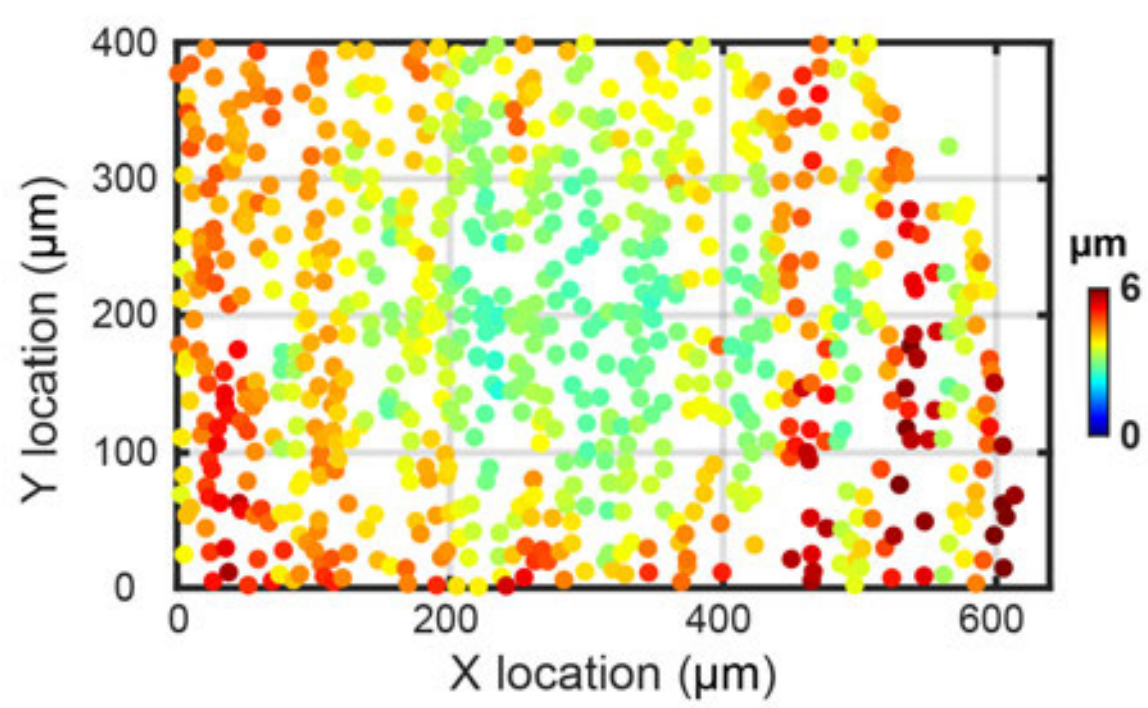

Reviewer #3:

Zhong and colleagues upgrade the free-space FACED two-photon fluorescence microscope (2PFM) and demonstrate its improved capabilities by performing in-vivo (1) large field-of-view (FOV) measurements of cerebral blood flow, (2) population (> 200 neurons simultaneously) voltage imaging at 385 Hz frame rate, and (3) volumetric calcium imaging of ~14,000 neurons down to 750 µm at 3.8 Hz volume rate in the mouse brain, as well as (4) "whole-brain" calcium imaging of zebrafish larvae.

The FACED module uses a cylindrical lens to focus a laser beam into a cavity made by a pair of high-reflectivity mirrors that are slightly misaligned but otherwise parallel, producing an array of beamlets that are both temporally delayed and angularly separated. This array of beamlets forms a line scan under a typical 2PFM, and thus the 1-MHz laser repetition rate is effectively turned into a line scan rate (along the fast axis, or FACED scanning direction). When combined with a galvanometer that scans orthogonally

(along the slow axis), the microscope achieves a kilohertz frame rate and can be applied to fast two-photon (2P) imaging. Compared to the original FACED microscope, FACED 2.0 reduces the temporal spacing between the beamlets to 1 ns, adds another galvo scanner along the FACED scanning direction to tile multiple adjoining scanning areas to one large FOV (e.g., 320 µm x 400 µm at 385 Hz frame rate), and uses a fast piezo motor to the objective to enable large-scale volumetric imaging (e.g., 1111 µm x 1000 µm x 400 µm at 3.8 Hz volume rate), with subcellular resolution (PSF < 1 µm lateral FWHM, and < 5 µm axial FWHM).
The FACED microscope is impressive in leveraging its ultra-high laser point scanning speed to image a large area/volume at a high speed. Most notably, simultaneous large-scale voltage imaging reveals synchronized subthreshold membrane potential oscillations at 3-6 Hz across groups of neurons, in good agreement with recently published voltage imaging results by a target-illumination confocal microscope [1]. Also, cerebral capillaries and calcium imaging down to 750 µm by the FACED microscope reaches the depth limit of a 2PFM.

Overall, this paper is well-structured, the ideas are clearly presented and easy to follow, and the methods descriptions are very detailed. However, I believe the authors may have overstated the performance and impact of the FACED microscope because of two major concerns in this work.

(1) Although the excitation pulses are well-separated spatially (0.8-um inter-pulse separation, comparable to the lateral FWHM resolution), their temporal delays of 1 ns are too short for the ~3-ns fluorescence lifetime of GFP-based fluorophores (such as JEDI-2P and GCaMP6s used in this work), leading to enormous pixel crosstalk on the detection side (i.e., the information obtained between adjacent pixels are largely the same). This will deteriorate the spatial resolution and effectively reducing the number of resolvable points by at least 3 times (1 ns vs 3 ns). Sampling much faster than the fluorescence lifetime is ineffective for improving the imaging speed.

We appreciate the reviewer bringing this point up. We agree that 1-ns inter-pulse delay is smaller than the fluorescence lifetime of our sensors, but we do not agree that our choice here degrades the performance of our microscope. Instead, as we detailed below, sampling as fast as we do here improves our ability to detect small neural activity signals.

Firstly, FACED is distinct from other 2PFM techniques that utilize temporal multiplexing for fast scanning. Those techniques utilize much longer delays (for example, 3 ns delay in https://www.nature.com/articles/nmeth.1552, 6.7 ns delay in https://www.nature.com/articles/s41592-021-01239-8, 8 ns delay in https://www.nature.com/articles/s41592-023-01820-3), because consecutive laser pulses and thus acquired data points sample distinct structures **tens of microns apart** along either lateral or axial direction. For such implementations, it is essential to have large temporal delay in order to avoid crosstalk of signals from distinct structures. In contrast, for voltage imaging with FACED 2.0, neighboring pulses in FACED probe structures that are **0.8 µm (for voltage imaging) or 1.4 µm (for calcium imaging) apart** along the X direction. Therefore, the neighboring pulses mostly sample the cell body of the same cells, and as the reviewer pointed out, contain similar activity information (as we discuss below, this is a feature, not a bug).

Second, for neuronal activity imaging, spatial resolution is important so far as it enables the structural compartments of interest to be resolved. Two types of sample preparation are common in neuronal activity imaging: sparse fluorescence labeling is utilized when synaptic imaging is desired, and dense fluorescence

labeling is utilized for population activity imaging from neuronal cell bodies. Although sparse labeling is not the focus of this work, FACED can resolve synapses (e.g., Fig. 1e). To further improve spatial resolution of synaptic imaging, in an ongoing work, we have developed a temporal deconvolution method that reassigns fluorescent photons to the pixels of their origin. For population imaging of somatic voltage and calcium activity of densely labeled neurons, the focus of this work, even with a small amount of crosstalk between neighboring pixels, FACED 2.0 clearly resolves neighboring cell bodies.

We want to emphasize here that for population imaging analysis, ROIs are selected to include the entire somata. The values of pixels inside each ROI are then averaged to obtain its activity trace, because these pixels contain the same information and pixel averaging gives rise to higher SNR. At our pixel sizes, each cell body is represented by on average 244 pixels for voltage imaging and 100 pixels for calcium imaging. With the FACED foci scanning from left to right, the only pixels that may be affected by lifetime are those at the left edge of the ROI, with their signal contaminated by pixels to the left of the ROI. Typically there are on average 17 pixels (for voltage imaging) or 9 pixels (for calcium imaging) at the left edge of the ROI, corresponding to 6.8% and 8.5% of total pixels for voltage and calcium ROIs, respectively.

In the case of voltage imaging, due to soma-targeting of JEDI-2P-kv, neuropil to the left of the ROIs had much lower brightness than the ROIs themselves. For calcium imaging, expression level in the neuropil was higher, but neuropil activity is of much smaller magnitude than somatic activity (e.g., see Fig. 3 of https://www.biorxiv.org/content/10.1101/061507v2.full.pdf). Together, these factors further minimize contamination in the activity traces due to fluorescence lifetime.

That crosstalk due to fluorescence lifetime minimally contaminates the activity traces of individual neurons is supported by quantitative analysis (Supplementary Fig. 7): If there is substantial crosstalk, then two neurons that are neighbors along the FACED scan direction (X axis) would have more correlated activity (because fluorescence from the neuron on the left would contaminate the signal of the neuron on the right) than two neurons that are neighbors along the Y axis. We calculated the Pearson correlation coefficients (PCC) between the activity traces of the nearest neighbors along X direction ("NNx", blue data points, Supplementary Fig. 7) and along Y direction ("NNy", blue data points, Supplementary Fig. 7) for both voltage and calcium imaging data:

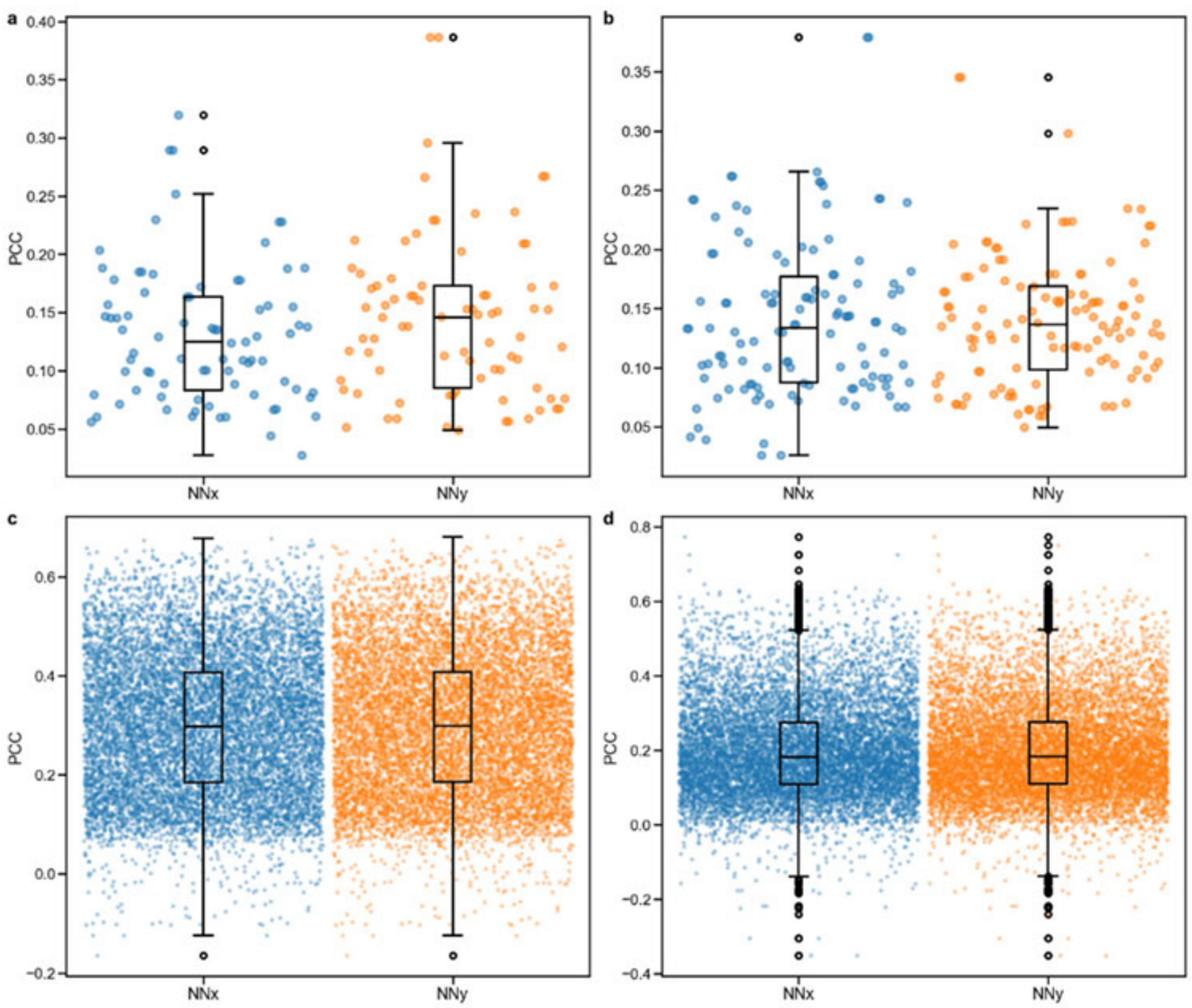

Supplementary Figure 7. Comparison of Pearson correlation coefficients (PCCs) between activity traces of neurons that are nearest neighbors along X axis (direction with potential contamination due to fluorescence lifetime) and Y axis (direction without the possibility of contamination). (**a,b**) Scatter and box-and-whisker plots of PCCs of voltage traces from Fig. 2 and Extended Data Fig. 11, respectively. (**c,d**) Scatter and box-and-whisker plots of PCCs of calcium traces from Fig. 3 and Extended Data Fig. 16, respectively. Blue dots and "NNx": PCC between two neurons that are nearest neighbors along X axis; Orange dots and "NNy": PCC between two neurons that are nearest neighbors along Y axis. Box-and-whisker plots: box represents interquartile range (IQR) extending from the first quartile to the third quartile; horizontal line indicates median; whisker extends to the most extreme data points within 1.5×IQR from box edge; black circles: outliers. One-sided t-tests comparing PCC(NNx) and PCC(NNy) yields p-values of 0.92, 0.42, 0.73, and 0.70 for the data in **a**, **b**, **c**, and **d**, respectively. Each scatter plot in **a**, **b**, **c**, and **d** contains data points from 81, 118, 9575, 9638 pairs of neurons, respectively.

As shown by Supplementary Fig. 7, the PCCs of activity traces of the closest neighbors along X axis and along Y axis exhibited similar distributions. One-sided t-tests on whether the PCC distribution along X is greater than the PCC distribution along Y have p-values of 0.95 (Supplementary Fig. 7a), 0.51 (Supplementary Fig. 7b), 0.73 (Supplementary Fig. 7c), and 0.70 (Supplementary Fig. 7d), indicating that there is no statistically significant difference between these distributions. Therefore, activity traces of neighboring neurons along X do not exhibit a higher correlation than those along Y, indicating that activity imaging does not suffer from contamination due to fluorescence lifetime.

Lastly, we would like to emphasize that we chose 1 ns inter-pulse delay not because we want to increase imaging speed per se. Instead, it allows us to optimize the scanning range (the spatial length of the linescans) and spatial sampling (the number of pixels/foci within the linescans). As we demonstrated throughout the manuscript, the new configurations enabled us to carry out blood flow measurements and activity imaging over large areas. For our applications, the advantages of having a longer scan range with dense spatial sampling substantially outweigh the minimal crosstalk due to fluorescence lifetime.

We added "Additional analysis indicates that the choice of 1 ns delay does not cause detectable contamination in activity imaging (**Supplementary Note 4**, **Supplementary Fig. 7**)" to Methods and included new Supplementary Note 4 and Supplementary Fig. 7.

(2) One impactful achievement that the authors intend to claim is that FACED microscope allows for optical recordings of both suprathreshold and subthreshold activities in > 200 individual neurons simultaneously. This claim is not convincing. First, to capture the temporal dynamics of ~2.1 ms FWHM in the action potential, the 2.6 ms/frame is too slow. The effect can be seen when one compares the voltage traces with those in the original FACED paper obtained with ASAP3, an indicator considered inferior to the JEDI-2P used in this work. The "spikes" detected in the voltage traces in this work look much less distinguishable than those in the FACED 1.0 work. It seems like the authors simply lower the spike detection threshold from SNR > 7.5 (FACED 1.0) to SNR > 2.5 (FACED 2.0, this work). Furthermore, the collected photon numbers per neuron per frame (171 photons at 75-th percentile) attest to the low SNRs of voltage traces. Assuming photon shot noise limit, a -20 % deltaF over F translates into an SNR of ~2.6, meaning the voltage spiking activities can only be detected with subpar or even questionable fidelity, in only a quarter of the neurons imaged. In fact, if one inadvertently inverts the voltage traces, the spike detection algorithm may still report a good number of "activities" in many of the neurons imaged. If this is the case, then the suprathreshold voltage data should not be considered trustworthy. Also because of the low photon counts, the authors' statement in the summary paragraph about the "dark state relaxation enhancing the photon yield by orders of magnitude" may not apply here either. Finally, when compared to similar large-scale voltage imaging work by other research groups, such as ref [1], the voltage imaging data presented here suffers from poor SNRs, and has no advancements in depth penetration or imaging FOV. However, the large-scale voltage imaging experiments in this work have their merit in detecting synchronized population-wide subthreshold oscillations at 3-6 Hz, which requires a much slower frame rate.

Below we address, with newly acquired data and additional analyses, the important points raised by the reviewer one-by-one.

1. The concern over whether 2.6 ms/frame is sufficient to detect the voltage response reported by JEDI-2P, which has a reported FWHM of ~2.1 ms for action potentials.

In the original JEDI-2P paper, the in vivo mouse brain imaging data, from which the FWHM of 2.1 ms for action potentials was determined, was acquired at 440 Hz (see Fig. 5, https://doi.org/10.1016/j.cell.2022.07.013) or 2.3 ms/frame. (To get the higher-temporal resolution trace to determine the FWHM, JEDI-2P paper utilized a spike-triggered analysis using the simultaneously recorded membrane potential with electrophysiology as described in "High-resolution optical spike waveforms" section of Method Details.) This was why we initially used 2.6 ms/frame for voltage imaging with FACED 2.0.

Fig. 5C of the JEDI-2P paper (reproduced below) shows simultaneously recorded membrane voltage trace acquired with in vivo juxtacellular recording (upper black traces) and JEDI-2P fluorescence traces acquired at 2.3 ms/frame (lower green traces). For optical recording, VolPy was used for detecting spikes associated with APs (the same method used in our manuscript, see below for more discussion on our spike detection method). Comparing the APs identified separately from electrical and optical recordings (black and green ticks), the authors of the JEDI-2P paper reported a Pearson's correlation coefficient of 0.64 ± 0.18 (range: 0.43–0.84 across individual neurons) between the two methods, with the lower correlation coming from

neurons with poorer SNRs. These previously published results support our statement in the original manuscript "Given that APs reported by JEDI-2P-kv had a full width at half maximum (FWHM) of ~2.1 ms[15], our imaging rate should be high enough to capture most APs."

[redacted]

During revision, we collected new datasets taken at 1.25 ms/frame (182 neurons recorded simultaneously) and 2.6 ms/frame (225 neurons recorded simultaneously), respectively, and now presented them in the updated Fig. 2 and Extended Data Fig. 11. We also added voltage traces with expanded scale in these figures. In addition, we also provided representative traces for all neurons imaged in these datasets in Extended Data Figs. 8 and 12. (Please see Pages 2-14 of this document.)

Compared with the dataset in Fig. 2 of our original submission, the new datasets have higher SNR (see Pages 33-37 below for a detailed discussion regarding SNR). However, none of the biological conclusions have changed, indicating that our previous 2.6 ms/frame data also have sufficiently sampling rate for revealing population voltage dynamics.

With data acquired at 800 Hz, we carried out additional analysis to determine whether ~400 Hz frame rate is sufficient for revealing most population activity characteristics. We down-sampled the 800 Hz data (by keeping alternate data points in time) to 400 Hz and then carried out the same analyses on correlated activity, timing and magnitude of visually evoked response, input-output function, and population orientation tuning properties (Extended Data Fig. 14):

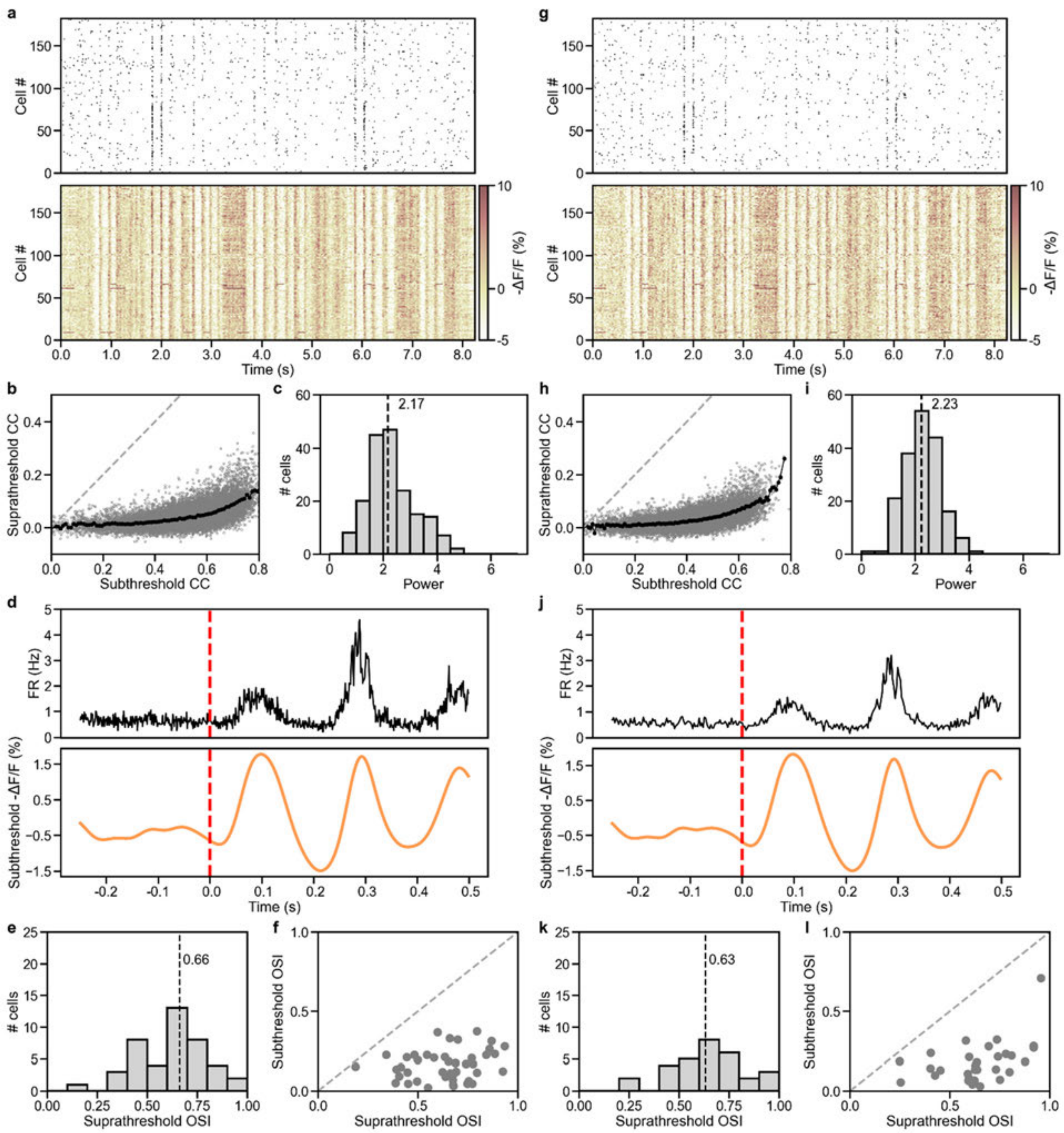

Extended Data Figure 14. Comparison of analyses from 800-Hz voltage imaging data and its 2× down-sampled (to 400 Hz) data. (**a-f**) Analyses of 800-Hz data (1.25 ms/frame; same as Fig. 2). (**g-l**) Analyses of down-sampled data (2.5 ms/frame). (**a, g**) Raster plots of (top) spikes and (bottom) subthreshold -ΔF/F traces of 182 neurons during a representative 8.25-s trial. (**b, h**) Suprathreshold versus subthreshold pairwise correlation coefficients (CC, gray dots) for 16,471 cell pairs from the 182 neurons in a. Black dots: binned averages with 0.01 bin size for subthreshold CC. Dashed gray line: reference line with slope = 1. (**c**, **i**) Histogram of fitted power-law exponent values of 182 neurons. Dashed line: median. (**d**, **j**) Average firing rate (black curve, top panel) and subthreshold -ΔF/F (orange curve, bottom panel) relative to the onset of drifting grating stimuli (red dashed line) extracted from 19,008 voltage traces of 88 neurons exhibiting visually evoked activity across 27 trials × 8 drifting grating orientations. (**e**, **k**) Histogram of suprathreshold orientation-selectivity index (OSI) of 43 orientation selective (OS) neurons in **e** and 30 OS neurons in **k**. Dashed line: median. (**f**, **l**) Scatter plot of subthreshold OSI vs. suprathreshold OSI for 43 OS neurons in **f** and 30 OS neurons in **l**. Dashed line: reference line with slope = 1.

Unsurprisingly, given the much slower variation of subthreshold activity with time, properties related to subthreshold voltage activity alone do not show perceivable differences between the original 800 Hz data and the down-sampled 400 Hz data (lower panels, Extended Data Fig. 14a,d,g,j). Measured spike rate is lower in the down-sampled 400-Hz data than in 800-Hz data (upper panels, Extended Data Fig. 14d,j), likely because the slower sampling rate misses some APs. However, both datasets have similar timing and temporal variation of firing rates (upper panels, Extended Data Fig. 14a,d,g,j).

Activity correlation across the population is insensitive to frame rate except for neuron pairs with highest correlated spiking activity (Extended Data Fig. 14b,h). Distributions of the exponent of the input-output function also have very similar median values (2.17 vs 2.23), with the main difference being that 800 Hz sampling revealed more highly nonlinear neurons (e.g., those with exponents larger than 3) than the down-sampled 400 Hz data (Extended Data Fig. 14c,i). These differences are likely caused by 400 Hz data tending to miss more APs during burst firing.

Although the numbers of neurons that were found to have visually evoked AP firing are the same for the 800 Hz and 400 Hz data (88 out of the total 182 neurons), the number of neurons with suprathreshold orientation selectivity is higher for the 800 Hz data (43 neuros at 800 Hz vs. 30 neurons at 400 Hz). However, OSI distributions are again highly similar between the two datasets (median: 0.66 vs. 0.63; Extended Data Fig. 14e,f,k,l).

The above analysis indicates that imaging JEDI-2P-kv-expressing neurons at ~400 Hz can inform on neuronal activity with similar accuracy as imaging at 800 Hz for most application scenarios. 400 Hz data become less accurate only when high-fidelity recording of high-frequency firing is required.

We now include the above analysis as Supplementary Note 3, as it provides guidance on choosing the optimal frame rate based on the biological questions under investigation.

We also added to Main Text:

Tiling four FACED FOVs for each frame, we can double the imaging area to 320 µm × 400 µm at a pixel size of 0.8 µm × 0.8 µm and 2.6 ms/frame. Previous characterization on JEDI-2P dynamics indicates that imaging at 2.6 ms/frame should be able to capture most APs[15]. In one mouse, we recorded voltage activities from 225 layer 2/3 visual cortical neurons simultaneously (**Extended Data Figs. 11-13**), and observed low photobleaching (**Extended Data Fig. 9c,d**). Importantly, the population voltage dynamics observed at 2.6 ms/frame are highly similar to **Fig. 2** data acquired at 1.25 ms/frame, including activity correlation, input-output function, timing and magnitude of visually evoked responses, and population orientation tuning properties (out of 225 neurons in **Extended Data Fig. 11**, 155 neurons exhibited visually evoked suprathreshold activity, and 86 neurons displayed suprathreshold activity with orientation selectivity).

To test how the frame rate of imaging data affects the extracted voltage dynamics, we down-sampled the 800 Hz data in **Fig. 2** to 400 Hz and compared the activity characteristics from the down-sampled data with the original data (**Extended Data Fig. 14**). Consistent with the observations above, 400 Hz sampling can inform on neuronal activity with similar accuracy as 800 Hz in most application scenarios for JEDI-2P-kv and 800 Hz is only required when accurate recording of high-frequency firing is needed (**Supplementary Note 3**).

We thank the reviewer for raising the question of frame rate, which has led to these analyses.

2. Regarding the SNRs and spike detection threshold of our voltage traces.

First, we apologize for having the wrong photon counts in our original submission. Our previous method underestimated the number of photons by 1.7 fold. We have recalibrated our detection system using a variance and Poisson statistics method during the revision. The photon counts for our new voltage imaging data can be found in **Supplementary Table 5** (reproduced below):

Figure number	Sample	75% percentile of photon counts in a ROI per frame	50% percentile of photon counts in a ROI per frame	25% percentile of photon counts in a ROI per frame	Average # of photon counts per ROI per frame	Standard deviation of photon counts per ROI per frame	Average # of photon counts per pixel within each ROI per frame
Fig. 2	Wild-type mouse transfected with JEDI-2P-kv voltage indicator	414.1	298.7	203.0	316.2	138.6	1.4
Extended Data Fig. 11		401.2	305.3	224.6	329.6	144.7	1.4
Fig. 3	Slc17a7-IRES2-Cre-D mouse	38.9	24.6	17.0	31.4	21.8	0.3
Extended Data Fig. 16	Wild-type mouse transfected with GCaMp6s	48.8	29.1	19.0	38.8	29.2	0.3

Note: Number of photons were calculated from the baseline (F_0) trace of each ROI, using the conversion factor of a photon corresponding to 1300 in pixel value. The conversion factor was measured assuming Poisson statistics.

Importantly, the new datasets do not change our biological conclusions, indicating that our previous data had sufficiently SNR for accurately revealing population voltage dynamics. On Page 17 of this document, we listed several factors that have contributed to the increased imaging throughput in FACED 2.0.

As detailed below, there is a misunderstanding on how we detect spikes in the Reviewer's comment that we "simply lower the spike detection threshold from SNR > 7.5 (FACED 1.0) to SNR > 2.5 (FACED 2.0, this work)".

Spike detection criteria in FACED 1.0 and FACED 2.0:

In FACED 1.0, spike detection was based on the signal-to-noise ratio (SNR), defined as $-\Delta F/\sqrt{F}$, where F represents baseline fluorescence and ΔF is the fluorescence change relative to baseline. Spikes were detected when SNR was above 7.5.

In FACED 2.0, spikes were identified based on ΔF/F traces, as we described in Methods: "Image sequences and ROI masks were inputted into VolPy, which extracted the voltage traces ΔF/F ("dFF" output from Volpy) and detected spikes. From spikes identified by VolPy, we further rejected spikes with its peak -ΔF/F value lower than the mean+2.5×SD within a 20-frame rolling window in the corresponding voltage trace."

As our description indicates, spike detection in FACED 2.0 is a two-step process:

We first employed VolPy, a template-matching based method, to detect spikes from voltage traces. VolPy's spike detection leverages signal information across multi frames (thus utilizing photons across multiple frames), allowing for more accurate spike detection than simple thresholding. Subsequently, we implemented a standard deviation (SD) based rejection step, which discards VolPy detection results with peak -ΔF/F values lower than mean+2.5×SD as described above. This rejection step further minimizes false spikes. Importantly, the 2.5 value here is NOT SNR, and should not be compared with SNR threshold of 7.5 used in FACED 1.0. Therefore, spike detection in FACED 2.0 is not a simple lowering of the SNR threshold from 7.5 to 2.5.

Below, we computed the SNR (defined as SNR = $-\Delta F/\sqrt{F}$) for spikes detected in the new FACED 2.0 datasets in Fig. 2 and Extended Data Fig. 11 using the two-step process. We found that 96.2 % and 75.1% of neurons, respectively, had spike SNR values greater than 7.5 (Supplementary Fig. 3). Furthermore, applying the $-\Delta F/\sqrt{F}$ thresholding criterion in FACED 1.0 to the FACED 2.0 data produced quantitatively similar population-level activity results (Supplementary Figs. 4,5).

Together these analyses indicate that spikes detected in FACED 2.0 and FACED 1.0 have similar SNR.

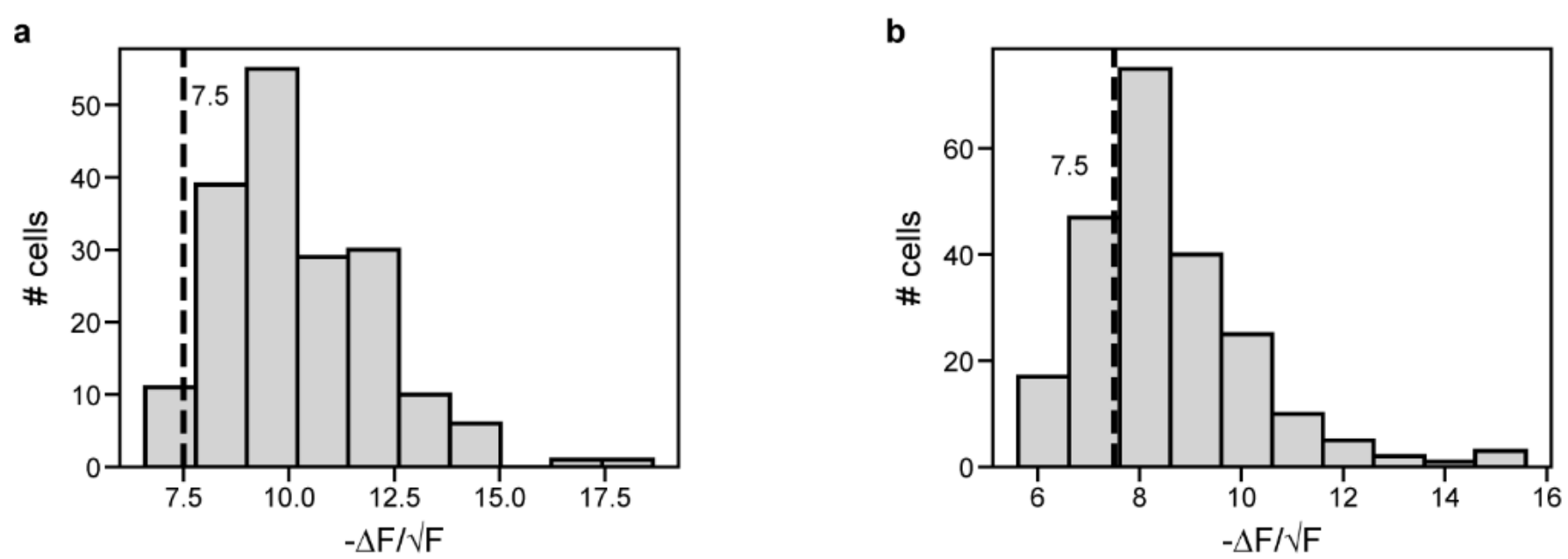

Supplementary Fig. 3. Histograms of the average -ΔF/√F of detected spikes for 182 neurons in Fig. 2 (a) and 225 neurons in Extended Data Fig. 11 (b). Dashed line: -ΔF/√F = 7.5. 96.2% neurons in **a** and 75.1% neurons in **b** have -ΔF/√F > 7.5.

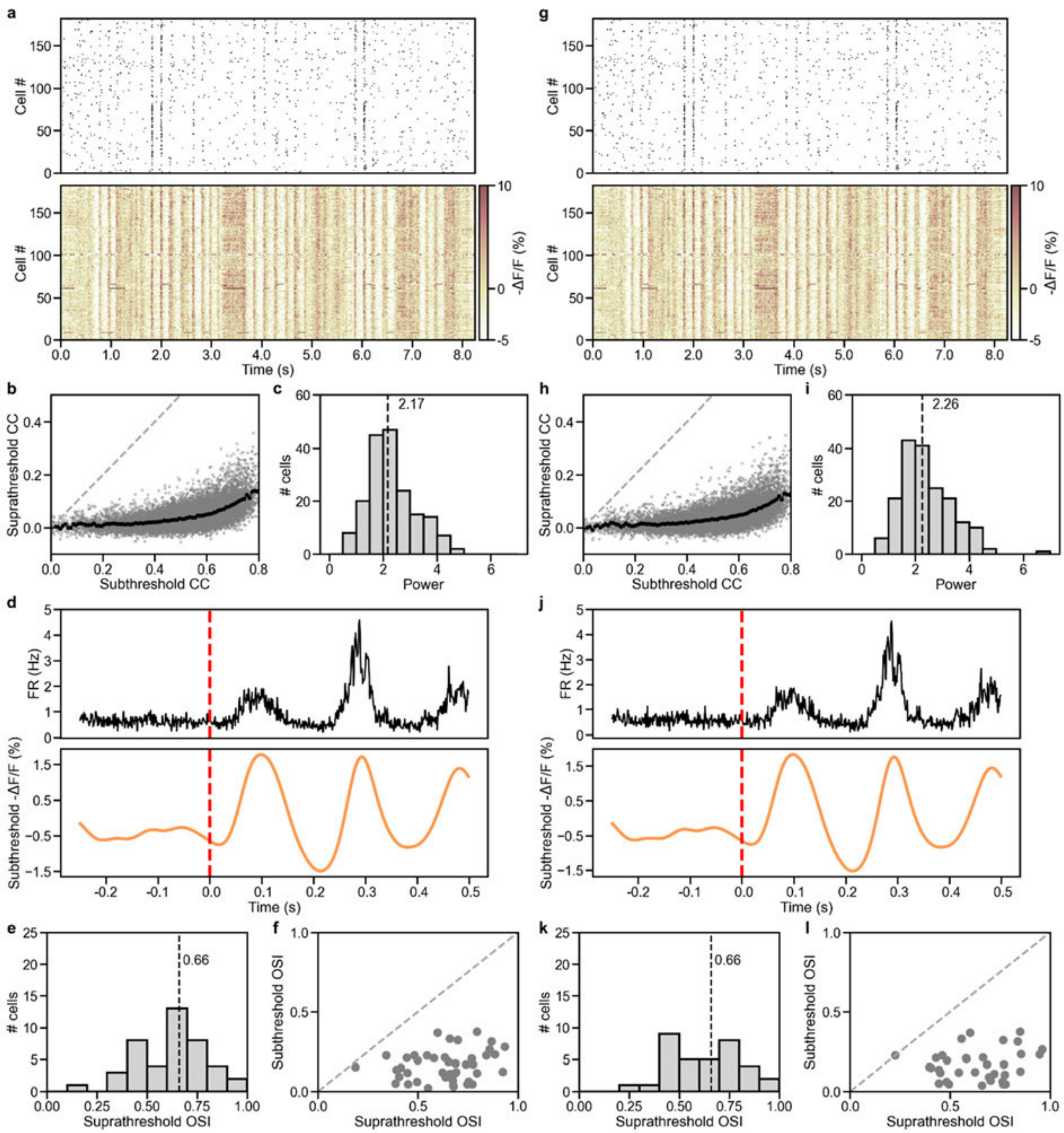

Supplementary Fig. 4. Comparison of voltage imaging analysis results of the dataset in Fig. 2, using two spike detection methods: our method (left) and -ΔF/√F > 7.5 threshold-based detection (right). (**a, g**) Raster plots of (top) spikes and (bottom) subthreshold -ΔF/F traces of 182 neurons during a representative 8.25-s trial. (**b, h**) Suprathreshold versus subthreshold pairwise correlation coefficients (CC, gray dots) for 16,471 cell pairs from the 182 neurons. Black dots: binned averages with 0.01 bin size for subthreshold CC. Dashed gray line: reference line with slope = 1. (**c**, **i**) Histogram of fitted power-law exponent values of 182 neurons. Dashed line: median. (**d**, **j**) Average firing rate (black curve, top panel) and subthreshold -ΔF/F (orange curve, bottom panel) relative to the onset of drifting grating stimuli (red dashed line). **d** is computed from 19,008 voltage traces from 88 neurons exhibiting visually evoked activity across 27 trials × 8 drifting grating orientations. **j** is computed from 16,416 voltage traces from 76 neurons exhibiting visually evoked activity across 27 trials × 8 drifting grating orientations. (**e**, **k**) Histogram of suprathreshold orientation-selectivity index (OSI) of 43 orientation selective (OS) neurons in **e** and 35 OS neurons in **k**. Dashed line: median. (**f**, **l**) Scatter plot of subthreshold OSI vs. suprathreshold OSI for 43 OS neurons in **f** and 35 OS neurons in **l**. Dashed line: reference line with slope = 1.

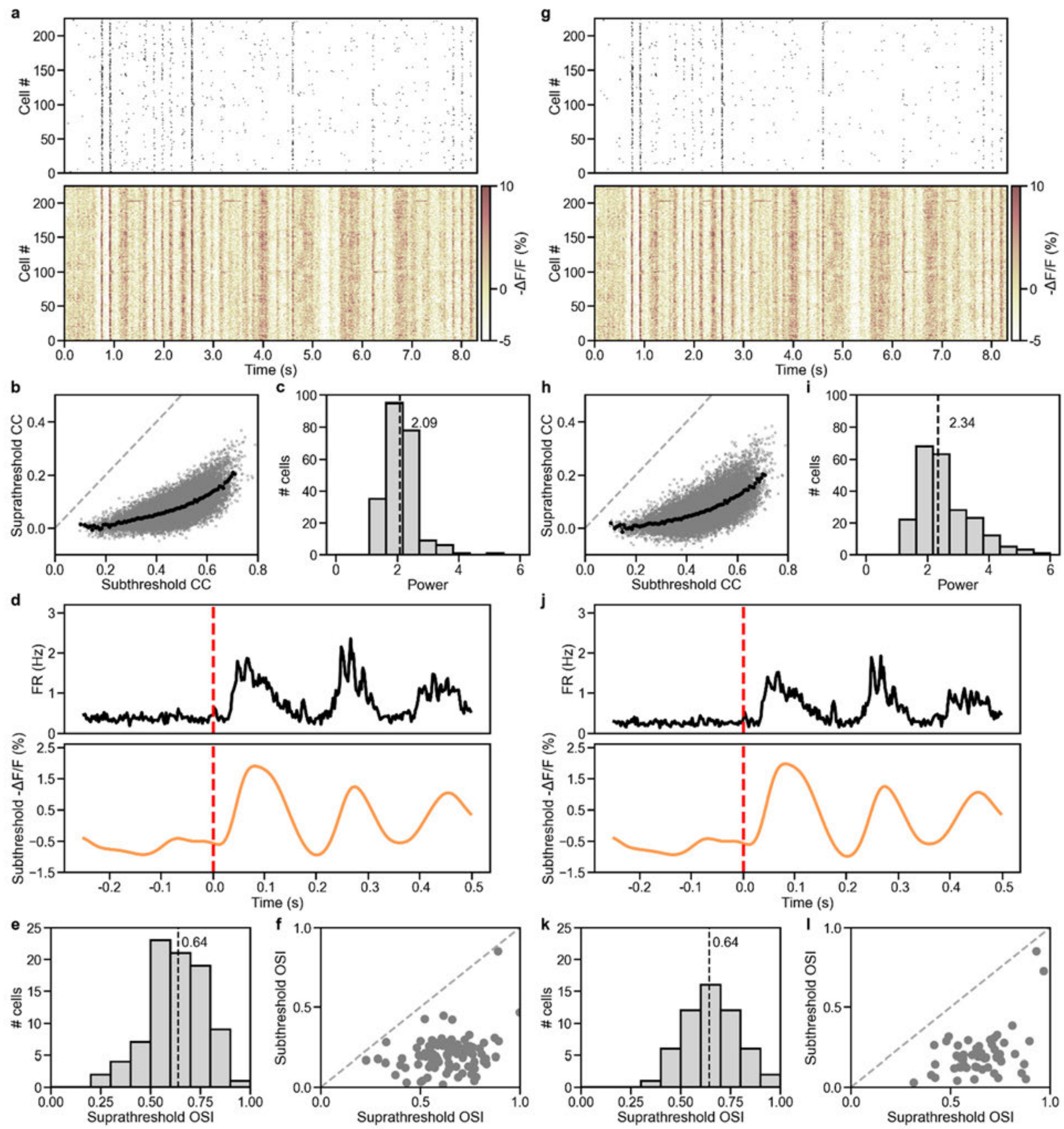

Supplementary Figure 5. Comparison of voltage imaging analysis results from the dataset in Extended Data Fig. 11, using two spike detection methods: our method (left) and -ΔF/ √ F > 7.5 threshold-based detection (right). (**a, g**) Raster plots of (top) spikes and (bottom) subthreshold -ΔF/F traces of 182 neurons during a representative 8.32-s trial. (**b, h**) Suprathreshold versus subthreshold pairwise correlation coefficients (CC, gray dots) for 25,200 cell pairs from the 225 neurons. Black dots: binned averages with 0.01 bin size for subthreshold CC. Dashed gray line: reference line with slope = 1. (**c**, **i**) Histogram of fitted power-law exponent values of 225 neurons. Dashed line: median. (**d**, **j**) Averaged firing rate (black curve, top panel) and subthreshold -ΔF/F (orange curve, bottom panel) relative to the onset of drifting grating stimuli (red dashed line) computed from 32,240 voltage traces from 155 neurons exhibiting visually evoked activity across 26 trials × 8 drifting grating orientations. (**e**, **k**) Histogram of suprathreshold orientation-selectivity index (OSI) of 86 orientation selective (OS) neurons in **e** and 55 OS neurons in **k**. Dashed line: median. (**f**, **l**) Scatter plot of subthreshold OSI vs. suprathreshold OSI for 86 OS neurons in **f** and 55 OS neurons in **l**. Dashed line: reference line with slope = 1.

Consistent with the above analysis, in representative traces for all neurons in Fig. 2 and Extended Data Fig. 11 (Extended Data Figs. 8 and 12), Spikes are easily visualized, indicating that the numbers of photons from each neuron are high enough for reliable identification of action potentials.

We further validated the accuracy of our spike detection pipeline using simulating voltage traces. The simulated traces contain supra- and subthreshold -ΔF/F activity similar to the in vivo data (at 1.25 ms time step size, Supplementary Fig. 6). The simulated suprathreshold spikes had 20% -ΔF/F and 2 ms FWHM. For simulated subthreshold activity, we set the mean peak fluctuation amplitude to be 8% ΔF/F. Poisson noise, corresponding to the photon count per frame, was then added to the ground-truth traces to approximate photon noise (Supplementary Fig. 6a).

We then processed these simulated traces with FACED 2.0's spike detection pipeline and calculated the true positive rates (the percentage of ground-truth spikes that were detected by our pipeline) and the false positive rates (the number of incorrectly detected spikes divided by the total number of time points of the recording) at different photon counts (Supplementary Fig. 6b,c). As expected, higher photon counts are associated with more accurate spike detection.

At the average photon counts for neurons in Fig. 2 (316.2 photons per ROI, Supplementary Table 5), our method achieved >90% true positive rates and ~0.04% false positive rates (i.e., ~4 false spikes in 10,000 time points or during a 12.5-s-long recording).

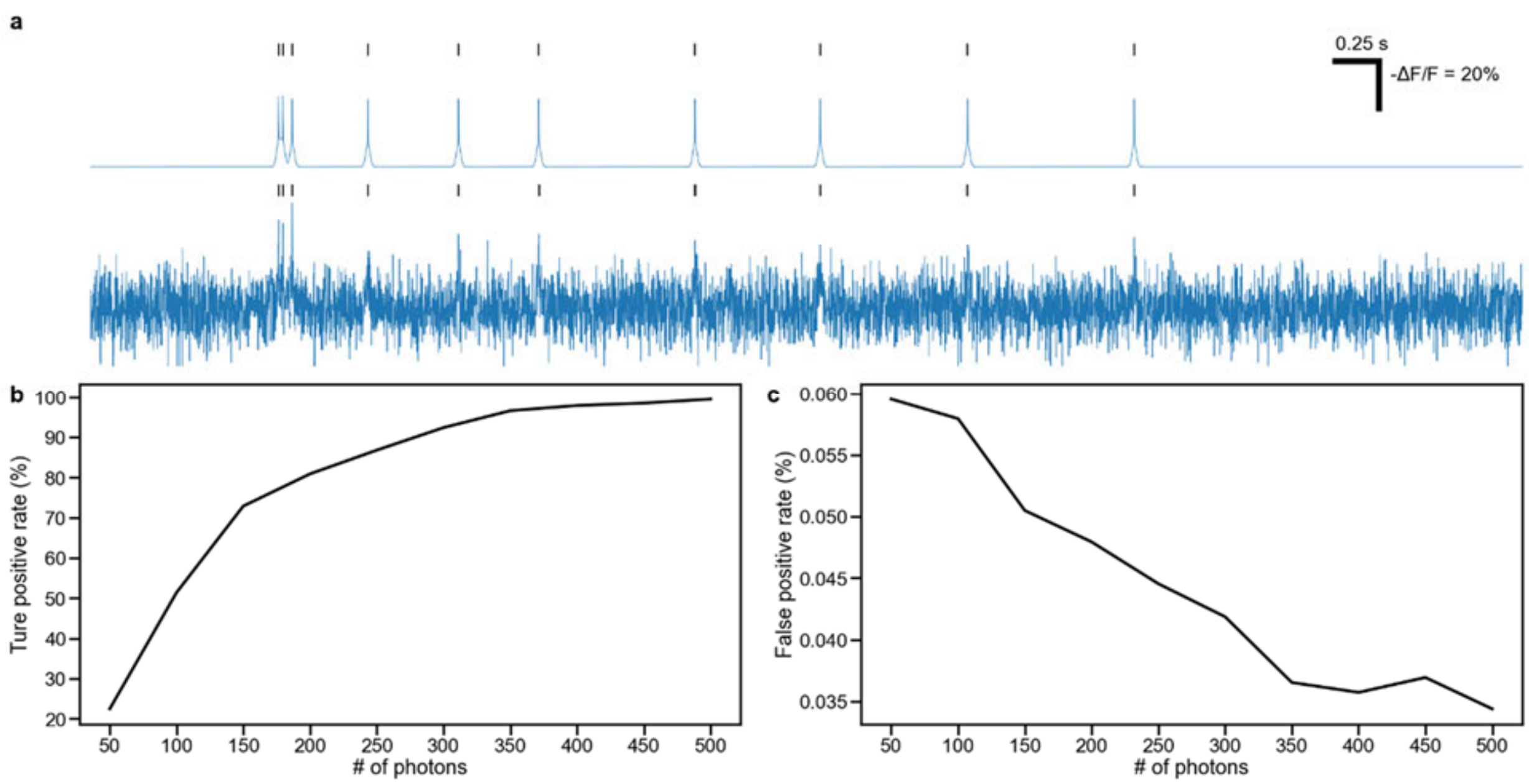

Supplementary Fig. 6. Validation of the spike detection method on simulated voltage traces. (**a**) Top trace: an example ground-truth voltage trace. Top black ticks: ground-truth spikes. Bottom trace: corresponding noise-added voltage trace with a SNR from 150 photons/frame. Bottom black ticks: spikes detected from noise-added voltage trace. (**b**) True positive rate of spike detection across different SNR conditions (SNR is quantified by number of photons per frame) (**c**) False positive rate of spike detection across different SNR conditions (SNR is quantified by number of photons per frame). For **b** and **c**, data point for each SNR condition is computed from 1000 simulated noisy traces with the number of spikes randomly selected (1 to 50) and randomly positioned within the trace duration (**Supplementary Codes**).

We now include the above analysis in Supplementary Note 2 and Supplementary Fig. 6.

3. Performance comparison of FACED 2.0 with recently published techniques including single-photon voltage imaging methods.

The reviewer stated that "when compared to similar large-scale voltage imaging work by other research groups, such as Ref [1] (Xiao et al Nature Methods 2024), the voltage imaging data presented here suffers from poor SNRs, and has no advancements in depth penetration or imaging FOV". We respectfully disagree that FACED 2.0 is inferior to single-photon (1P) methods.

(i) Above, we already addressed the concerns on SNR.

(ii) FACED 2.0 uses 2P excitation, which is intrinsically optically sectioning and therefore does not suffer from out-of-focus contamination. In contrast, 1P voltage imaging methods require either sparse labeling or sparse illumination to reduce out-of-focus contamination. As a result, fewer neurons per unit area are recorded simultaneously with 1P methods. Below we compare our FACED 2.0 FOVs with a FOV from Ref [1]. All FOVs are plotted on the same spatial scale.

[redacted]

As shown by the above comparison, the sparsity requirement limits 1P methods to biological inquiries on neurons that are further away from one another. Furthermore, crosstalk/contamination due to out-of-focus fluorescence excitation needs to be carefully removed for 1P methods but is not a concern for 2P methods such as FACED 2.0.

We added the following the discussion section of Main Text to further distinguish FACED 2.0 from 1P methods: "Whereas either sparse labeling or sparse illumination is required for population voltage imaging utilizing single-photon excitation[54,55], the intrinsic optical sectioning through two-photon absorption enables FACED 2.0 to be applicable to activity imaging in dense neuronal populations."

[redacted]

[redacted]

[redacted] synapses.

[redacted]

New 2P voltage indicators with better brightness and sensitivity would enable FACED to image voltages from submicron structures such as neuronal processes and synapses.

In conclusion, our new data and analyses have shown that FACED 2.0 is a versatile microscopy technique with subcellular resolution that can be applied to both sparsely and densely labeled samples and simultaneously record voltage and calcium activity from a large number of neurons. It offers substantial advancements as well as complement existing techniques and would be of substantial interest to the *Nature Methods* readership.

Minor comments:

• Even after normalization, the discontinuity in both spatial structure and laser illumination between adjoining areas in the FACED scan direction is visible. How do the authors deal with fluorescence

fluctuations, say in neurons along the edges of tiled FACED scan, caused by motion artifacts convoluted with this discontinuity?

The discontinuities between FACED FOVs were caused by overlapping (along X) and slight mismatch (along Y) between neighboring FACED FOVs, not by sample motion. Mice were habituated to head fixation prior to experiments, and we observed no visible image motion during 94% of the experimental duration. In rare instances when we observed sample motion, those images were excluded from further analysis. We do not analyze neurons that are cut off at the edges.

• In Fig. 2e, why is the firing rate discrete (i.e. only 0, 100 Hz, and 200 Hz)?

The discrete firing rates represented by the gray dots in Fig. 2e of the original manuscript (now Fig. 2f and Extended Data Fig. 11f) result from the discrete sampling of the data acquisition system. These gray dots indicate the raw firing rate and subthreshold estimates derived from the voltage traces. To estimate the firing rate, we employed a 10-ms rolling window. Due to the nature of discrete digital sampling, only a discrete number of spikes can be detected within this estimation window, leading to the observed discrete firing rates. For instance, when analyzing the voltage imaging dataset acquired at 1.25 ms/frame, the firing rate estimation window encompasses eight frames of digital sampling. The spike positions are recorded as time points corresponding to each frame, allowing for a potential count of spikes ranging from 0 to 8 within the estimation window. This yields potential firing rates of 0/(8 × 1.25 ms) = 0 Hz, 1/(8 × 1.25 ms) = 100 Hz, 2/(8 × 1.25 ms) = 200 Hz, 3/(8 × 1.25 ms) = 300 Hz, 4/(8 × 1.25 ms) = 400 Hz, 5/(8 × 1.25 ms) = 500 Hz, 6/(8 × 1.25 ms) = 600 Hz, 7/(8 × 1.25 ms) = 700 Hz and, 8/(8 × 1.25 ms) = 800 Hz.

• Given the axial PSF and the voxel size, the microscope cannot image the entire volume; i.e. the axial sampling is not dense enough to cover every plane in the volume. It is therefore misleading to claim the volume size or whole-brain volumetric imaging of zebrafish larvae.

We appreciate the reviewer's suggestions. Using a stricter definition of "whole-brain imaging" in which all neurons were imaged, our Z step size is too big to ensure sampling all neurons. We have rephrased our description of the volumetric imaging capabilities to "For animal models with smaller brains such as zebrafish larvae, FACED 2.0 is capable of performing simultaneous calcium imaging of multiple planes across the brain volume."

• For Figs. 1 and S4 to S6, 1035 nm was used for imaging Thy1-GFP mice. The 2P cross section at 1035 nm for GFP is very low compared to 920 nm. What's the reason to use 1035 nm instead of 920 nm? Is this for deeper penetration? Given the much lower 2P cross section, is this a better choice to image deeper?

Indeed, 920 nm would have been a better choice than 1035 nm for green fluorophores. In addition to the GFP data mentioned by the reviewer, we utilized 1035 nm excitation for voltage imaging, which is also not ideal for JEDI-2P. The reason for choosing 1035 nm for these experiments was because, during the time span of the project, the optics within our optical parametric amplifier system (OperaF, Coherent Inc.) developed damage spots and degraded optics, which caused low output power, poor mode quality and pulse-to-pulse stability for its 920 nm output. We used 1035 nm output from the pump laser (Monaco, Coherent Inc.) instead for later experiments because it has superior mode quality and stability. Laser issues apart, our ability to acquire voltage data even at non-optimal excitation wavelength of 1035 nm suggests that with a better optical parametric amplifier, the performance of FACED 2.0 would be further improved. The

continued development in laser technology would also improve the accessibility and performance of our technique.

• It appears that the same excitation power was used for imaging for all depth (150 to 200 mW), but the number of frames averaged was increased for deeper imaging, for example, from 400 (S4) to 4000 (S5 and S6). For imaging at 750 to 800 um depth, however, the signal would decrease by ~1000x. Therefore, this 10x increase in time cannot compensate for the increase in imaging depth unless ~ 100x less signal was obtained in the deepest regions of the 3D volume. Please quantify the image quality in the deepest regions in terms of photons/frame.

The primary goal of these figures (now Extended Data Figs. 5,6) is to demonstrate FACED's capability to resolve subcellular structures at up to 750 µm depth *in vivo*, which is clearly supported by these figures. The ~1000× value given by the reviewer is an overestimation of how much signal decays with depth. We compared the fluorescence intensity of similar neuronal structures in raw images acquired at depths of 50 µm and 750 µm and observed a 79.3 fold and 76.0 fold signal decrease for images in Extended Data Fig. 5 and 6, respectively (see below). As requested, we also estimated the number of photons captured per single frame for the raw datasets at 750 µm for Extended Data Fig. 5 and 6, yielding average values of 7122 and 1996 photons per frame, respectively. (Note that these values are the photons per frame, thus depend on the fluorescence labeling density at these depths.)

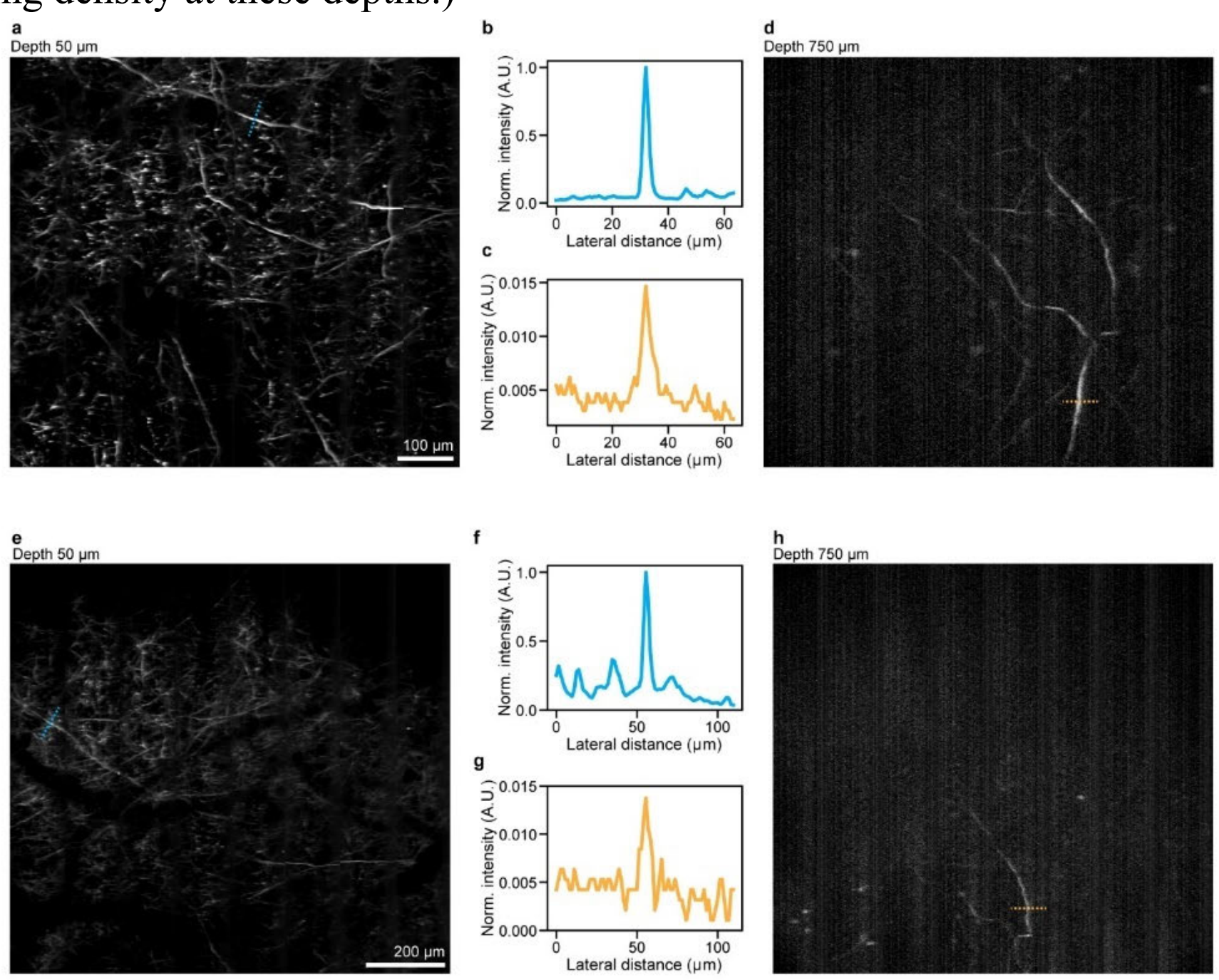

Brightness comparison of neuronal processes in the Thy1-GFP line M mouse cortex at depths of 50 µm and 750 µm. (**a**) Neuronal processes at 50 µm depth imaged with 25× 1.05 NA objective. (**d**) Neuronal processes at 750 µm depth imaged with 25× 1.05 NA objective. (**b**) Lateral signal profile along the blue dashed line in **a**. (**c**) Lateral signal profile along the orange dashed line in **d**, normalized to the peak brightness in **b**. (**e**) Neuronal processes at 50 µm depth imaged with 16× 0.8 NA objective. (**h**) Neuronal processes at 750 µm depth imaged with 16× 0.8 NA objective. (**f**) Lateral signal profile along the blue dashed line in **e**. (**g**) Lateral signal profile along the orange dashed line in **h**, normalized to the peak brightness in **f**. (**a-d**) are from **Extended Data Fig. 5**, which were acquired using a 25× 1.05 NA objective, with a 640 µm × 585 µm field of view (FOV) and pixel size of 0.8 µm × 0.65 µm, under 1035 nm excitation at 140 mW post-objective. (**e-h**) are from **Extended Data Fig. 6**, which were acquired using a 16× 0.8 NA objective, with a 1,111 µm × 1,035 µm FOV and pixel size of 1.4 µm × 1.15 µm, under 1035 nm excitation at 170 mW post-objective.

• What is the power throughput of the FACED module + microscope?

The power throughput of the FACED module + microscope is 9.3%. We added this information to **Methods**.

• In supp. table 1, it is meaningless to compare pixels/voxels per unit time. Comparison of the number of resolvable points per unit time is more reasonable. Also, the authors tend to understate the performance of other microscopes. Simple examples include maximum imaging depths of 600 μm in Light Beads Microscopy, and 540 μm in Scan Multiplier Unit. Instead, the table puts 500 μm for both works. There may be more instances. If this table is to be published, please make sure it is correct.

We apologize for the errors in the original table. We missed these values when reviewing these papers – it was not our intention to underrepresent the performance of other microscopy systems. We have revised the Table to correct these errors.

We used pixels or voxels per unit time as a comparative metric, following Demas et al. Nat Methods 18, 1103–1111 (2021). We still believe that this metric is of value: as we detailed in response to your Comment 1, having more pixels per unit time enables more pixels to be acquired from each ROI (e.g., cell body) per unit time. Averaging more pixels in each ROI leads to higher SNRs in population activity imaging.

The goal of Supplementary Table 1 is not to show that FACED 2.0 has the highest pixels/voxels per unit time among all the 2PFM techniques. Indeed, it does not, as other techniques have even higher pixels/voxels per unit time values (e.g., multifocal excitation, light beam microscopy). Instead, by including other metrics such as lateral and axial resolution, maximal demonstrated imaging depth, area, and volume, Supplementary Table 1 shows that FACED 2.0 is a versatile microscopy technique with subcellular resolution that can be applied to both sparsely and densely labeled samples and simultaneously record voltage and calcium activity over large area/volume, thus offering substantial advancements over existing 2PFM techniques.

• In supp. Table 4, data is missing for the rest of the figures.

Supplementary Table 4 (now Supplementary Table 5) contains the photon counts for ROIs in voltage and calcium imaging experiments. To provide similar information for the other figures in the manuscript, we would need to drawing ROIs surrounding all the fluorescent structures, a labor-intensive process that does not provide more insight. Instead, in the new Supplementary Table 4, we provided the pixel values, calculated by averaging the pixel values that are above 99% of each image frame, for all the images. This allows for easy comparison of brightness levels across all the figures presented.

We thank both reviewers for their comments. Please see below our detailed response:

Reviewer #2 (Remarks to the Author):

"In this revised version, the authors addressed most of my previous concerns. The manuscript is significantly improved and can be, in my opinion, of interest for the broad audience of Nature Methods.

I have a few minor points that should be considered:

1) Response to previous point #2 of this referee: while I understand that the filtering process makes it difficult to estimate the SNR on subthreshold activity, I think that stating that subthreshold signals are of good quality is not enough. Perhaps the authors can plot the amplitude distribution of the subthreshold signal to provide a more quantitative assessment of the quality of this signal."

Thank you for your suggestion! Below are the amplitude distributions of the raw and low-pass-filtered subthreshold signals for the example neurons in **Fig. 2b,c**, as well as those for all the neurons in **Fig. 2**.

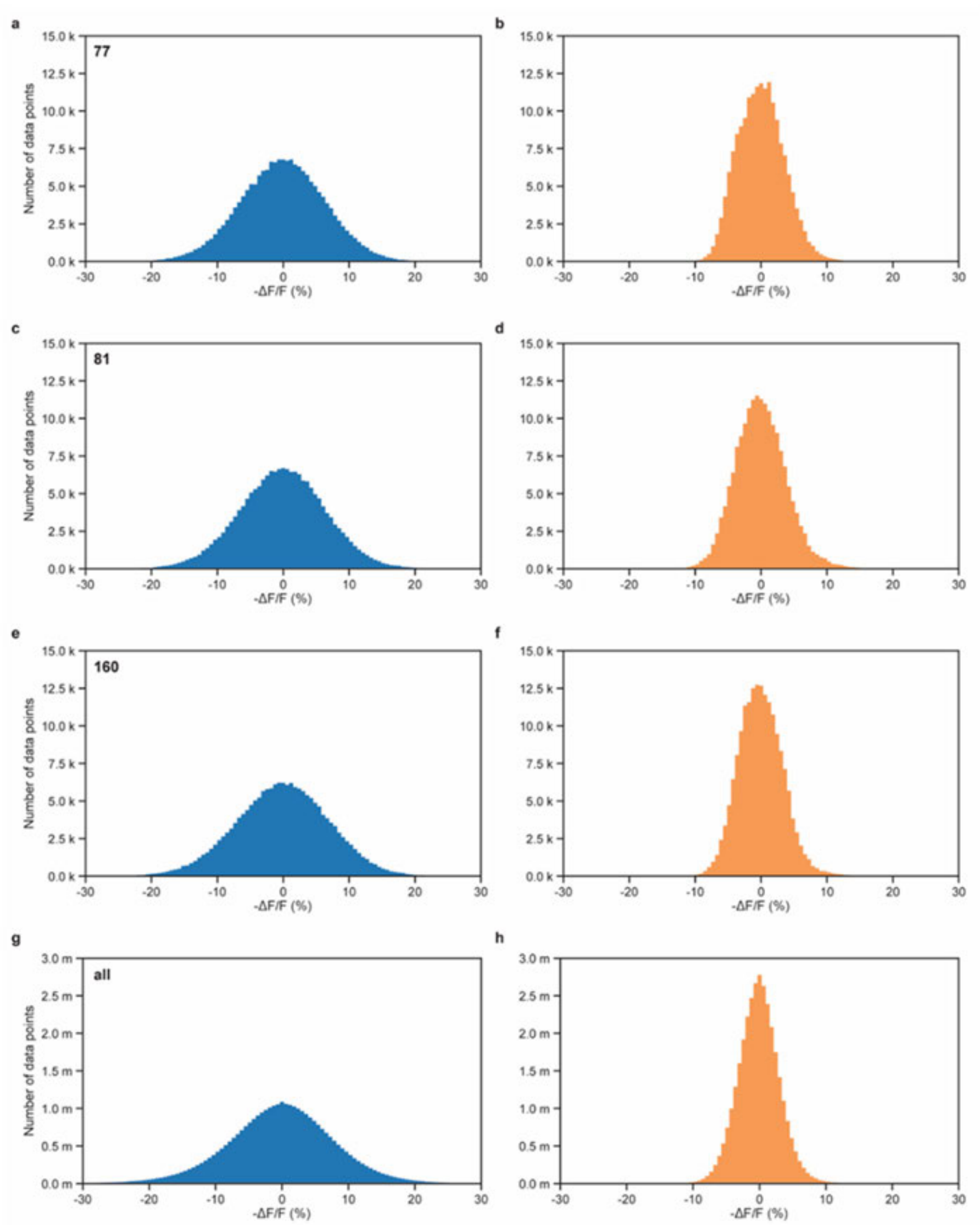

Amplitude distributions of the subthreshold ΔF/F traces. Blue: histograms for raw subthreshold ΔF/F traces (after excluding time points associated with spikes); Orange: histograms for low-pass-filtered subthreshold ΔF/F traces (with a 20-Hz 5th-order Butterworth lowpass filter). (**a,b**) Distributions for ROI 77 in Fig. 2. (**c,d**) Distributions for ROI 81 in Fig. 2. (**e,f**) Distribution for ROI 160 in Fig. 2. (**g,h**) Distribution for all 182 neurons in Fig. 2. Y-axis units indicate the number of data points: "k" for thousands and "m" for millions of data points.

As indicated by these distributions, low-pass-filtering sharpens the distributions by suppressing high-frequency noise (see below the example traces in **Fig. 2b,c**), without shifting the average.

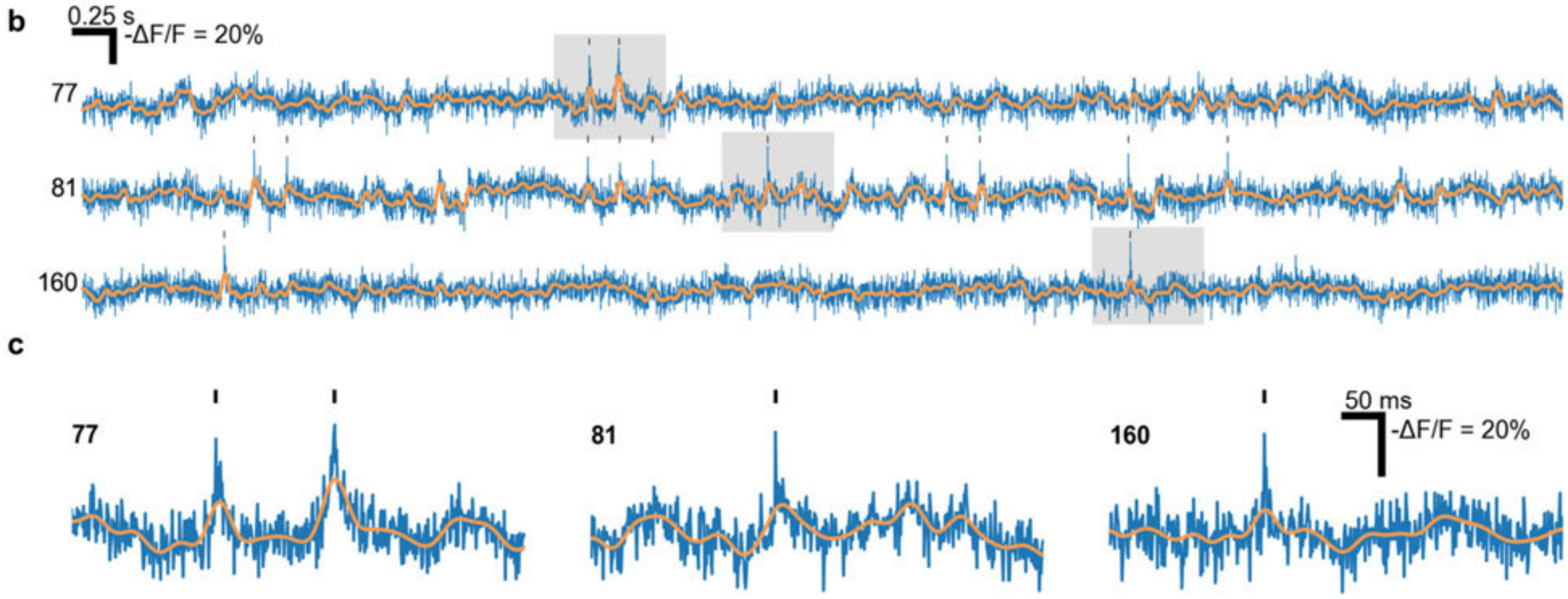

During an example 8-s-long visual stimulation trial, the low-pass-filtered subthreshold traces exhibited highly synchronized activity (see below the subthreshold panel of **Fig. 2d**; see 53 more examples in **Extended Data Fig. 10** and **Extended Data Fig. 13**).

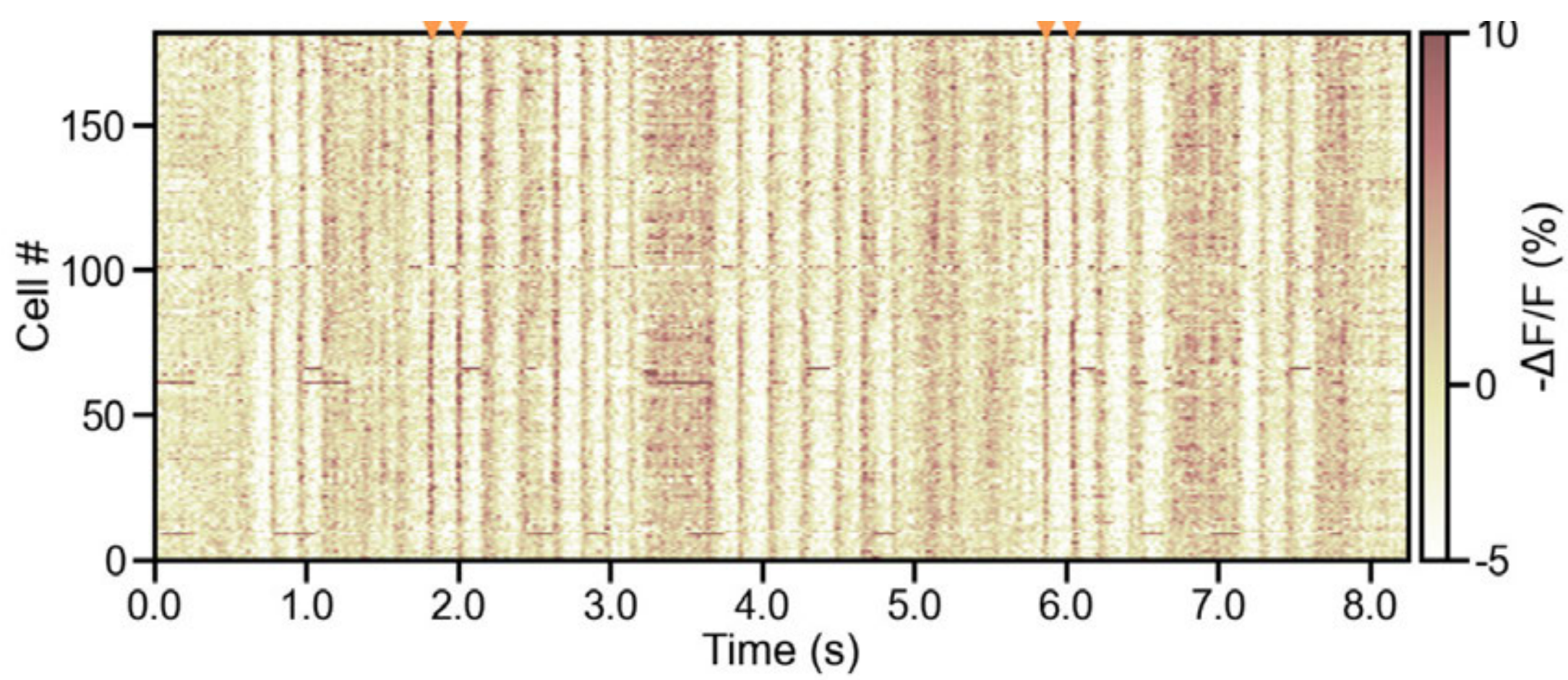

Such synchrony would not have been present if the subthreshold responses were too noisy (because photon noises for individual neuronal traces are not correlated).

"2) Current Supplementary Figure 1: please provide spatial scale and intensity scale. Is the intensity scale the same in the ground truth (left) and volumetric (right) images?"

The intensity scales are not exactly the same but very similar. Please see below the revised figures with scale bars.

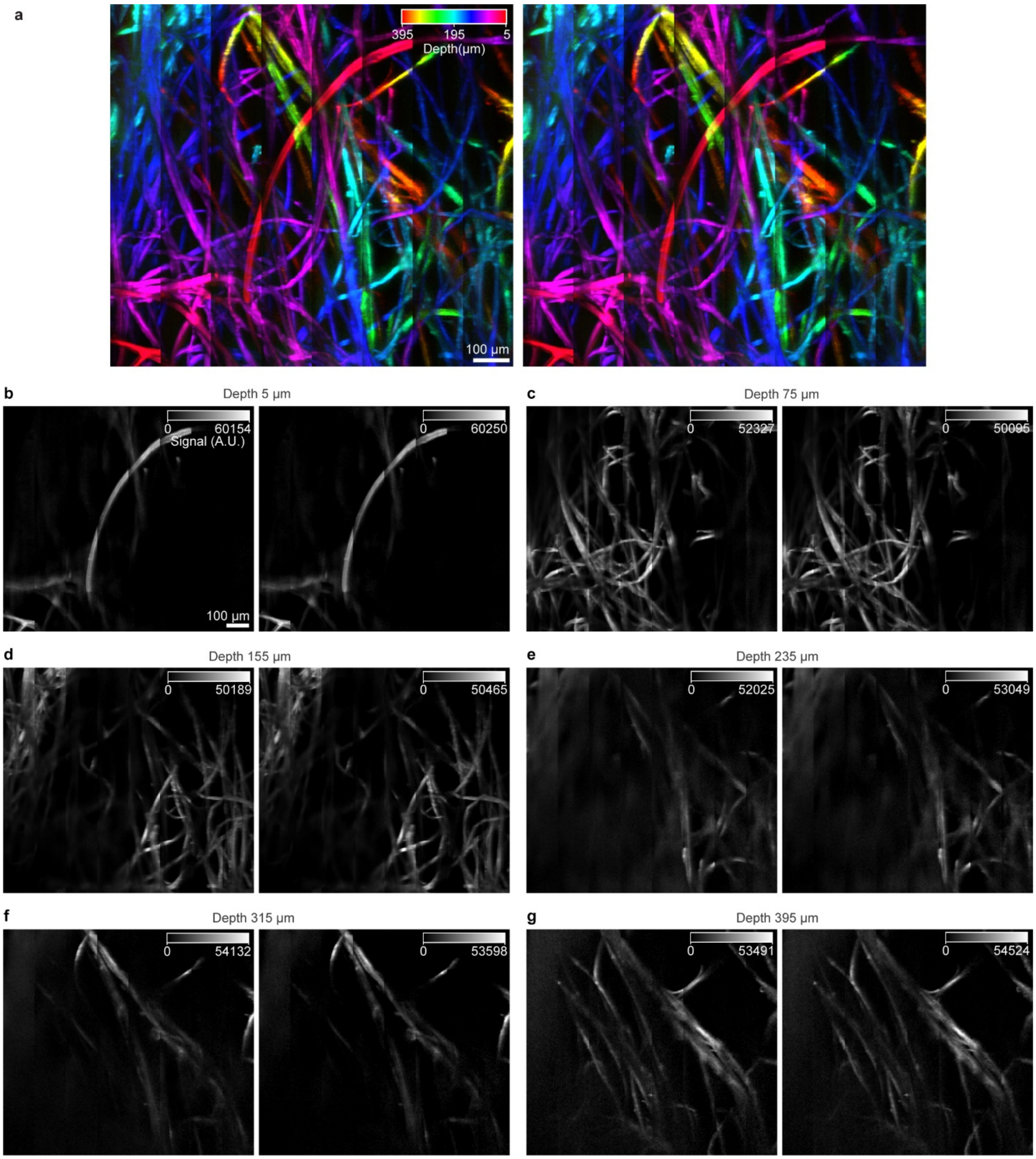

Supplementary Fig. 1

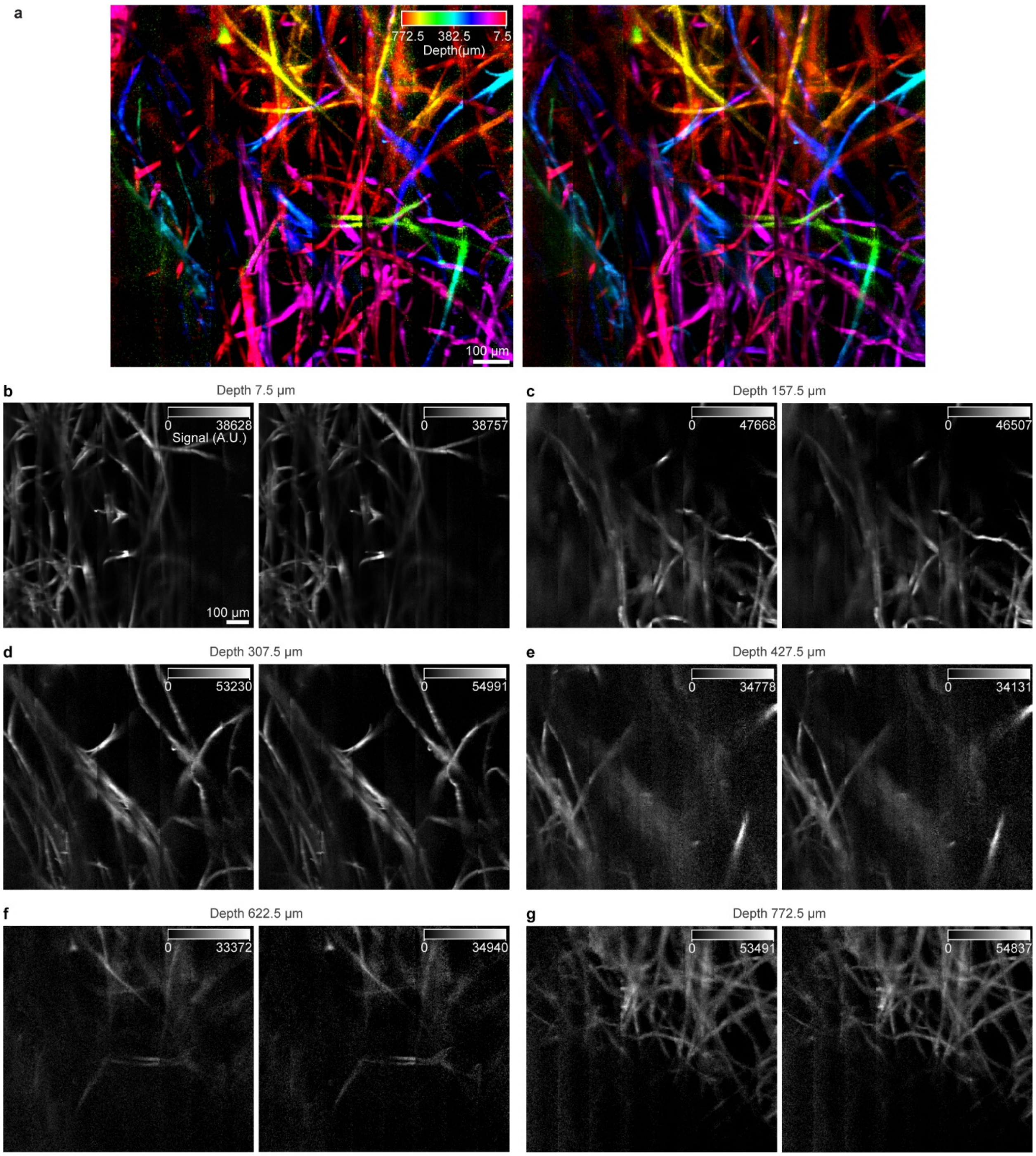

Supplementary Fig. 2

We have updated **Supplementary Fig. 1** and **Supplementary Fig. 2** to include scale and intensity bars.

"3) A short text should be added to the discussion stating that 1035 nm laser excitation was used in the study and therefore that the performance of FACED 2.0 should significantly improve when 920 nm laser light will be used to excite JEDI-2P and GCaMP6s."

We added the following text to the second-to-last paragraph of Main Text:

“(Note that although 920 nm excitation would be more optimal for JEDI-2P-kv, 1035 nm excitation was used here due to limitations of the laser system. A better match between excitation wavelength and voltage indicator would further improve the performance of FACED 2.0.)”

“4) A short sentence should be added to the discussion stating the limitation of FACED regarding the need of regular and frequent alignment (every 5-30 minutes). This is critical information for experimenters willing to implement FACED 2.0 in their laboratories.”

We added the following as the new last paragraph of Main Text:

“Finally, due to room temperature fluctuations, laser pointing instability, and mechanical drift, FACED 2.0, as configured here, requires realignment of the FACED mirror pairs every 5-30 minutes. Better mechanical design, improved laser systems, and active beam stabilization would enable hands-free operation in the future.”

Reviewer #3 (Remarks to the Author):

"We appreciate the authors' careful responses to all concerns, questions, and comments raised by both reviewers. To improve the quality of the data and enhance the credibility of the methods, the authors corrected their photon counts calibrations, performed new voltage imaging experiments which yielded higher photon counts (1.5 x) at double the frame rate, and compared the voltage imaging data obtained at 800 Hz and 400 Hz. Upon reading the additional data and re-examining the entire manuscript, we believe that the major issues are still not resolved. We list them below.

"1. Compared to FACED1, FACED2 reduced the pulse (pixel) separation from 2 ns to 1 ns. The authors claimed that this is one of the main improvements of FACED2. We respectfully disagree. The short delay time between adjacent pixels using FACED microscopes is a major limitation for imaging fluorescence, with most fluorescent probes have fluorescence lifetimes of 2 to 6 ns. For GFP-based probes (lifetime ~ 3 ns), and at 1 ns delay between pulses, the fluorescence overlap between adjacent pixels is ~ 72%. Put it in another way, the adjacent pixels mostly have the same information even though they are spatially separated."

In our previous round of response, we already addressed this concern in detail (Pages 26-29, **Supplementary Note 4, Supplementary Fig. 7**). The reviewer's statement that "the adjacent pixels mostly have the same information even though they are spatially separated" is exactly what we explained in our previous response (Page 26-27): because consecutive pulses mostly sample the same cell body and contain the same information, the temporal overlap of their fluorescence signal is not a source of contamination.

"Therefore, the number of independent pixels per line is effectively reduced by ~ 2x."

<u>The improvement from FACED 1.0 to FACED 2.0 was NOT about the increased number of independent pixels.</u> As we detailed in our previous response on Page 27, for population calcium and voltage imaging of cell bodies, pixels within the cell bodies are always averaged to generate the final activity traces. Whether these pixels are independent or not is not relevant for these measurements.

Reducing time delay from 2 ns in FACED 1.0 to 1 ns in FACED 2.0 enables us to have a larger field of view (FOV) while still sample each cell body with enough pixels to detect the small signal change associated with voltage activity.

"One may argue that the overlap in fluorescence can be subtracted out, but this will inevitably increase the photon noise. Depending on the signal strength difference between the adjacent pixels, this additional photon noise can be overwhelming."

We did not argue that the overlap in fluorescence can be subtracted out and we did not do such subtraction here.

"The authors argued that this overlap in fluorescence may not be important for imaging the soma where the pixels are binned anyway."

Our statement that overlapping in fluorescence is not an issue is supported by our quantitative analyses (**Supplementary Note 4**, **Supplementary Fig. 7**).

"This statement essentially says that when imaging the soma, a high spatial resolution is not important. While this may be true in some situations, it remains a major limitation as a fluorescence microscope."

The reviewer misunderstood our argument. We never claim that "when imaging the soma, a high spatial resolution is not important". As experts on using adaptive optics to improve 2PFM imaging resolution in the brain in vivo, we deeply understand and appreciate the importance of spatial resolution. In fact, we stated explicitly in the manuscript: "Importantly, FACED 2.0 maintained subcellular resolution in XY and cellular resolution in Z (**Fig. 1c**), which are essential for resolving individual neurons in 3D and accurately measuring their activity."

"In addition, if a low spatial resolution can be used for imaging the voltage signal, a much better way is to use a larger focal spot and higher pulse energy (e.g., twice the spot area with twice the pulse energy at half the "effective repetition rate"). Due to the two-photon response, the resulting signal will be higher."

Low spatial resolution should not be used for imaging voltage signals. Voltage indicators are localized on cell membrane. High spatial resolution is required for both spatially resolving the voltage signal from different neurons (especially along the axial direction) and generating high focal intensity for two-photon excitation. Reducing spatial resolution would lead to contamination of voltage signals and would require higher average power that may cause damage. (Even for calcium imaging, where calcium indicators typically fill the cytosol, high axial resolution is required - We are currently working on a manuscript that shows that it is essential to have high axial resolution, such as those provided by FACED 2.0, for accurate calcium imaging of cell bodies.)

"Therefore, to improve from FACED1, FACED2 should not reduce the time separation between the pulses to 1 ns to increase the nominal number of pixels per line (even 2 ns is too short for many fluorescent probes). Instead, FACED2 should have enlarged the focal spot size and used less number of foci (while maintaining the same average power) to maximize the performance of FACED microscope."

As explained above, reducing resolution is the wrong approach. Reducing time delay from 2 ns in FACED 1.0 to 1 ns in FACED 2.0 enables us to have a larger field of view (FOV) while still sample each cell body with enough pixels to detect the small signal change associated with voltage activity. We have also already provided ample evidence that 1 ns pulse delay is not a problem. Our claim of superior performance of FACED 2.0 over FACED 1.0 is supported by large-scale calcium and voltage imaging datasets that show new enabling capabilities of our microscope (e.g., voltage imaging of hundreds of neurons in FACED 2.0 versus ~tens of neurons in FACED 1.0).

"The authors went on to claim that "this (1 ns separation between the pulses) is a feature, not a bug". We disagree. We would like the authors to compare (1) FACED with 1 ns pulse separation and 100 foci vs (2) a setup (e.g., reverb or lightbeads, not using FACED) with 10 ns pulse separation and 100 foci, which will fill the 1000 ns between the pulses of a 1-MHz laser. What is the advantage of (1) FACED approached with 1 ns delay vs (2)? If there is no advantage, then the short delay time between adjacent pulses is a limitation of the FACED approach, not a useful feature."

The request for us to "compare (1) FACED with 1 ns pulse separation and 100 foci vs (2) a setup (e.g., reverb or lightbeads, not using FACED) with 10 ns pulse separation and 100 foci, which will fill the 1000 ns between the pulses of a 1-MHz laser" is neither reasonable nor practical.

Neither Reverb (https://www.nature.com/articles/s41592-019-0728-9) nor Light Beads (https://www.nature.com/articles/s41592-021-01239-8) have been shown to have the capacity for population voltage imaging. FACED utilizes ultrafast X scanning within the focal plane, with consecutive excitation pulses landing at pixels 0.8 µm apart. As a result, FACED can densely sample the neuronal membrane of each neuron in 2D at 800 Hz, enabling voltage imaging. Reverb and Light Bead use ultrafast Z scanning, with consecutive laser pulses landing at pixels tens of microns apart in Z. They cannot densely sample neuronal membranes in 2D at kilohertz. Both techniques also have much lower spatial resolution (axial resolution: 19-30 µm for Light Beads; 10 µm for Reverb) than FACED 2.0 (axial resolution for 1.05-NA objective: 2 µm). These factors likely explain their lack of population voltage imaging capability. Therefore, we do not agree that a comparison as suggested by the reviewer is meaningful.

In addition, the reviewer's proposal that we should build a Reverb or Light Beads microscope with 100 foci and 10 ns pulse separation is far beyond the capabilities of the published implementations of these techniques. The Reverb paper demonstrated 6 foci and 6.7 ns pulse separation, while the Light Beads paper employed 30 foci and 6.7 ns pulse separation. In other words, the reviewer wants us to build a Reverb or Light Beads system with a 25× and 5× gain in optical path length than previously published implementations, which would require the excitation light to propagate over 3000 meters (1.9 miles) on the optical table. The developers of Reverb or Light Beads would likely balk at building systems as requested.

More generally, we would like to push back on requesting researchers to build a previously published microscope system in order to prove technological advancements. Such endeavor can take years to complete. For this reason, in published microscopy literature, advances are claimed based on achievable performance, rather than using different microscope systems to image the same samples.

“2. The quantification of the SNR of the voltage and Ca imaging. For fast and/or large scale recording of neuronal activity, the SNR is perhaps the most important parameter to quantify the performance of the system. We found the revised manuscript still does not provide adequate quantifications of the SNR, and the SNRs we calculated from the data reported appear to be too low.

The SNR of the voltage recording should be calculated directly from the photon counts per neuron per frame (F) and the measured (or reported) delta F/F for the probe. The main text cited a peak delta F over F0 of ~ 20 %, which is consistent with the previously reported AP response of JEDI-2P-Kv. With a reported photon counts of ~ 300 photons/neuron/frame (Supplementary Table 5, 50% percentile), the calculated SNR should be ~ 3.5. This is substantially below the SNR reported for FACED1.”

In the previous round of review, the reviewer wrote “It seems like the authors simply lower the spike detection threshold from SNR > 7.5 (FACED 1.0) to SNR > 2.5 (FACED 2.0, this work)”. In response, we provided detailed analysis on SNR and spike detection threshold (Pages 33-37 in our previous response).

In that response, we explained that the FACED 1.0 paper defined SNR as $-\Delta F/\sqrt{F}$ and spikes were detected when SNR was above 7.5. Therefore, to directly compare the SNR of FACED 1.0 and FACED 2.0 as requested by the reviewer, we used that same definition of SNR and showed that most voltage traces acquired in FACED 2.0 had spikes above the 7.5 threshold (**Supplementary Fig. 3**).

We also provided photon counts for all imaging data, from which readers can calculate SNR using their preferred definition. We believe that we have provided all the information needed to evaluate the performance of our microscope.

“We would like the authors to directly compare the SNR of FACED2 vs FACED1 to show improvement.”

We did not claim that FACED 2.0 is better than FACED 1.0 because its voltage traces have higher SNRs. Instead, we showed that FACED 2.0 can acquire voltage traces at ***sufficient*** SNRs to enable population voltage imaging over a much larger FOV (e.g., 320 µm × 400 µm). As requested by the reviewers, we provided example traces for hundreds of neurons (182 neurons in **Extended Data Figure 8** and 225 neurons in **Extended Data Figure 12**). We validated our spike detection accuracy (Pages 33-37 in our previous response, **Supplementary Note 2**, **Supplementary Fig. 6**), which showed that our voltage traces had sufficient SNRs to enable analyses on neuronal properties (e.g., tuning, correlation, input-output function). FACED 2.0 example voltage traces having lower SNRs than the example traces in FACED 1.0 does not reduce the accuracy of these analyses.

In summary, the advance of FACED 2.0 over FACED 1.0 is not about having higher SNR voltage traces. In our previous revision, in response to a comment by Reviewer 2, we added **Supplementary Note 5** to compare FACED 2.0 with FACED 1.0. We reproduce the Note below:

“FACED 2.0 substantially enhances the imaging capabilities of FACED 2PFM by offering larger imaging FOVs and longer continuous imaging durations than FACED 1.0, in addition to having volumetric imaging capability. These improvements, achieved via both hardware and software optimization, allow for selecting imaging area/volume/throughput based on the temporal dynamics of biological events, enabling the study of larger neuronal populations over wider spatial domains and longer timescales at higher throughput than FACED 1.0.

In FACED 1.0, the FACED module was configured to generate linescans containing 80 pixels at a 2 ns inter-pixel delay, with its imaging FOV restricted to 50 µm × 250 µm (25× 1.05 NA objective). In FACED 2.0, the FACED module is configured to generate linescans containing 100 pixels at a 1 ns inter-pixel delay, with a single FACED FOV reaching 80 µm × 585 µm (25× 1.05 NA objective) or 140 µm × 1035 µm (16× 0.8 NA objective).

A complete rewrite of the microscope control software for FACED 2.0 allows us to increase Y galvo scanning to 833 Hz (1.67 kHz frame rate) over 585 µm for 25× 1.05-NA objective and 1,035 µm for 16× 0.8 NA objective, a substantial improvement over from FACED 1.0 with 500 Hz Y galvo scanning over 250 µm for 25× 1.05-NA objective. Adding an additional X galvo that scans along the FACED linescan direction in FACED 2.0 allows tiling

FACED FOVs to further enlarge the area that can be scanned at high speed. For example, using the 16× 0.8 NA objective, an area of 1111 µm × 1000 µm can be scanned at 192 Hz.

The control software also synchronizes 2D FACED imaging with continuous axial scanning of the microscope objective, enabling volumetric imaging. The large FOV and high frame rate of FACED 2.0 enable calcium activity of >10,000 neurons to be collected over large volumes at true cellular resolution (e.g., 1111 µm × 1000 µm × 780 µm at 365.7 ms/volume).

FACED 1.0 utilized a PXIe-5160 digitizer (National Instruments), which limits the maximal continuous imaging duration to 6 s. FACED 2.0 uses a high-speed digitizer with onboard FPGA (ADQ7-DC, Teledyne SP Devices). Together with a custom multi-thread data acquisition program that optimizes data transfer and storage, it enables continuous data streaming, limited in its duration only by disk storage size."

"For Ca-imaging, we can convert the reported pixel values (average of pixel values in the top 99% of image frame, Supplementary Table 4) to photon counts using the calibration factor (~ 1300) provided by the authors. For example, at 517 um depth, the photons/neuron/frame is (195/1300)*100 ~ 15 (or ~ 41 photons/neuron/s), which is not sufficient for Ca-imaging (using the d' calculation for GCaMP6s). Therefore, data for neuronal activity recording at depth beyond 500 um has too low an SNR to be useful. To show the ability to image throughout the 750 um depth, the authors need to show, for each depth, the distribution of the d' values for the calcium data."

Here the reviewer refers to **Extended Data Fig. 16**. We note here that the data included in the same figure and **Extended Data Fig. 17** clearly show that we had enough photons for calcium imaging, and images acquired at those depths provided biologically useful information. **Extended Data Fig. 16** presents calcium activity of neurons evoked by 3-s-long gratings (each trial including a 3-s blank screen, a 3-s drifting grating, and then a 2-s blank screen; a total of 8 different gratings were presented). The images of neurons at 517 µm were shown in panel e:

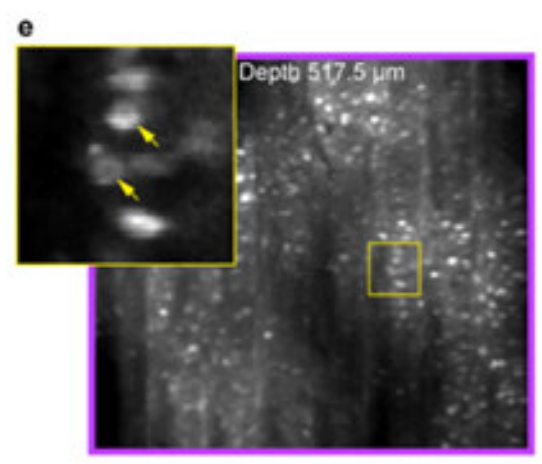

The example traces for these neurons measured during a 64-s-long recording with 8 different grating directions were shown in the upper purple box within Panel g:

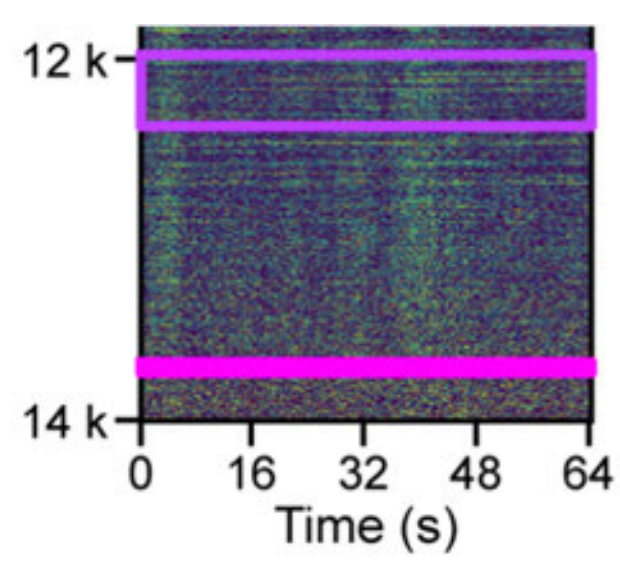

The responses from these neurons during a total of ~60 min of recording were shown in **Extended Data Fig. 17** (neurons numbered the same way as the figure panel above):

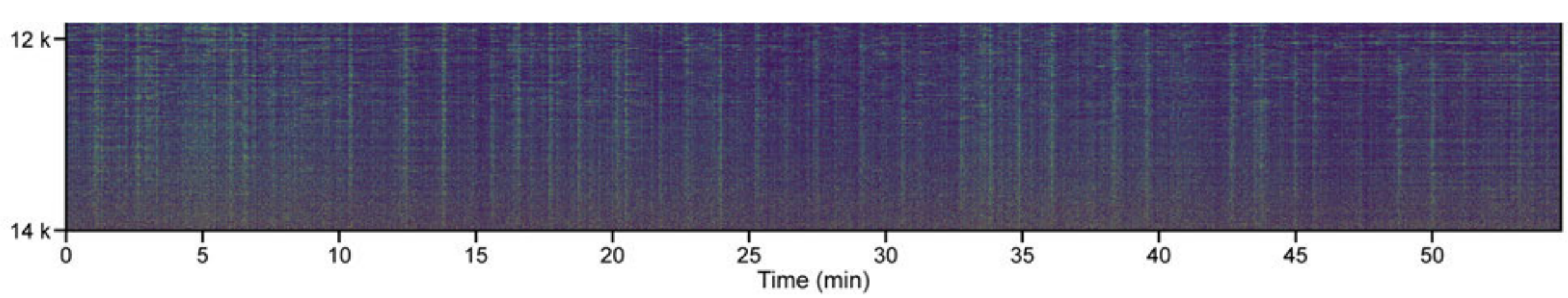

In these traces, calcium activity from neurons at 500 µm and deeper were clearly visible.

Why does the reviewer's calculation not agree with our experimental data? Neurobiologists do not use a single data point (or the data point within a single second, as calculated by the reviewer) to determine the presence of activity. For our analysis on visually evoked activity, we included the 8 frames acquired during the 3-s-long visual stimulation and the 5 frames acquired during the 2 s after the grating stimulation (to capture the calcium decay of GCaMP6s). Photons collected during these 5 seconds are sufficient for our algorithm (and our eye) to detect calcium activity from a single trial.

Furthermore, in visual neuroscience, it is standard experimental procedure to repeatedly present the same drifting grating stimulus and average the single-trial responses for each neuron. We presented the trial-averaged responses of neurons at depth in panel h, where the visually evoked responses were even more evident:

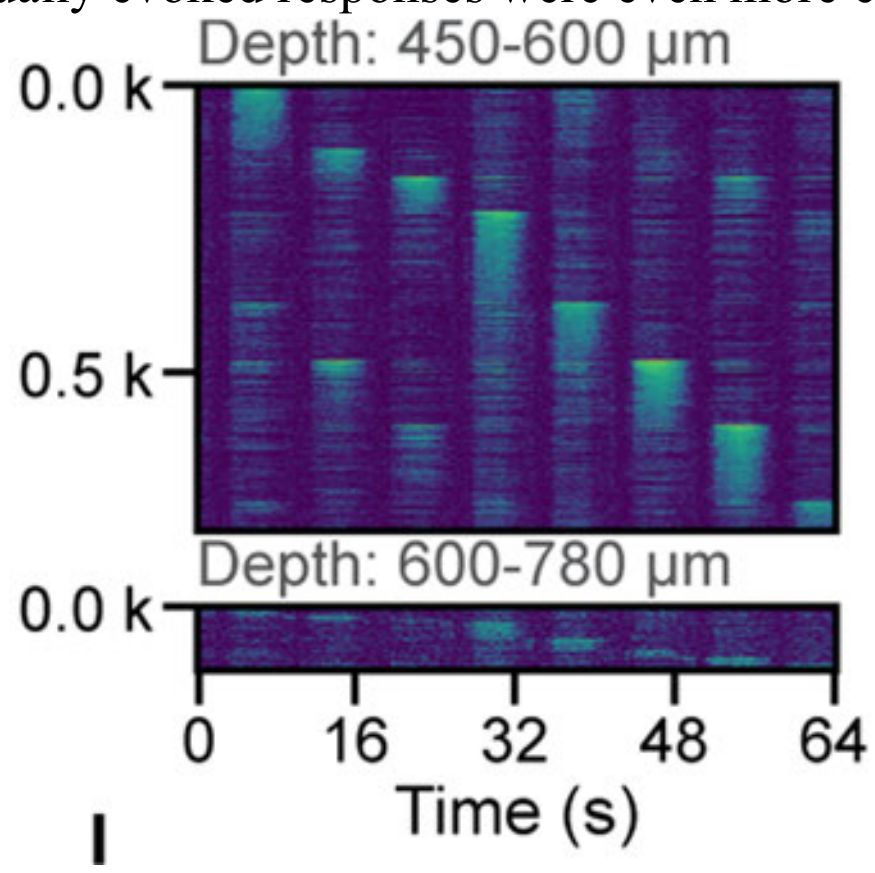

In summary, our data clearly supports the ability of FACED 2.0 to detect calcium activity from neurons at large depths.

3. The use of a constant excitation power throughout the imaging depth of ~ 750 um.
This approach for imaging a 3D volume is very unusual, and it is certainly not optimum. It's not clear why the authors did this instead of increasing the excitation power with increasing imaging depth. We would like the authors to provide some reasons for this approach. '

We did not vary excitation power with image depth because doing so would require the inclusion and synchronization of a Pockel cell, which increases system cost and complexity. More importantly, using the same power worked for the data presented. If varying power during volumetric imaging proves crucial for certain applications, we can certainly incorporate a Pockel cell in the future.

Even more mysterious is the fact that the authors reported a total fluorescence signal attenuation of ~50x (GCaMP6s, 920 nm excitation, Supplementary Table 4) from 97.5 um to ~ 757.5 um, and ~ 80x (1035 nm excitation, in the rebuttal letter) from ~ 50 um to ~ 750 um. These attenuation factors appear to be off by one order of magnitude, and these data will lead to scattering lengths for the mouse brain in vivo of ~ 340 um at 920 nm and ~320 nm at 1035 nm. These scattering lengths are 1.5x to 2x longer than typical data reported in the past at these wavelengths (by many research groups). Would this be measurement errors? The authors need to provide the explanations.

There was no mistake in our power measurement. We always used a calibrated power meter to measure power. And the pixel values for images at different depths were directly read out from the raw images. The reviewer's critique in the previous round of review "For imaging at 750 to 800 μm depth, however, the signal would decrease by ~1000×" appears to be similarly based on calculation using attenuation lengths. However, experimentally we did not see such a large signal decrease in vivo, as we showed in the previous response.

The fact that FACED 2.0 could image calcium activity from GCaMP6s-expressing neuronal cell bodies at 757.5 μm depth using 140 mW of 920 nm excitation is consistent with the performance of microscopes built by us and others. Below is a table with a list of publications where neurons were imaged at similar depths at similar power.

Ref.	Structure imaged	Label	Depth	Power	λ	Microscope	Reference
**From Ji lab**							
1	Dendrite and cell body	YFP	500 µm	75 mW	900 nm	Homebuild system 1 Ji lab	Wang *et al.* Multiplexed aberration measurement for deep tissue imaging *in vivo*. Nat Methods **11**, 1037–1040 (2014). https://doi.org/10.1038/nmeth.3068
2	Cell body	GCaMP6s	600 µm	140 mW	920 nm	Homebuild system 2 Ji lab	Wang *et al.* Direct wavefront sensing for high-resolution in vivo imaging in scattering tissue. Nat Commun 6, 7276 (2015). https://doi.org/10.1038/ncomms8276
3	Dendrite	YFP	750 µm	87 mW	960 nm		
4	Cell body	GCaMP6s	550 µm	90 mW	900 nm	Homebuild system 3 Ji lab	Sun *et al.* Thalamus provides layer 4 of primary visual cortex with orientation- and direction-tuned inputs. Nat Neurosci 19, 308–315 (2016). https://doi.org/10.1038/nn.4196
5	Dendrite	GCaMP6s	0-612 µm	36-96 mW	920 nm	Janelia mesoscope (https://elifesciences.org/articles/14472)	Lu *et al.* Rapid mesoscale volumetric imaging of neural activity with synaptic resolution. Nat Methods 17, 291–294 (2020). https://doi.org/10.1038/s41592-020-0760-9
6	Cell body	GCaMP6s	50-650 µm	24-96 mW	920 nm		
7	Capillary	FITC	39-710 µm	24-128 mW	920 nm	FACED 1.0 Ji lab	Meng *et al.* Ultrafast two-photon fluorescence imaging of cerebral blood circulation in the mouse brain in vivo. PNAS 119, e2117346119 (2022). https://doi.org/10.1073/pnas.2117346119
8	Dendritic spine	GFP	300–620 µm	38-65 mW	920 nm	Optimized Thorlabs Bergamo® II Ji lab	Yaeger *et al.* A dendritic mechanism for balancing synaptic flexibility and stability. Cell Reports, 43, 114638 (2024). https://doi.org/10.1016/j.celrep.2024.114638
**Examples from other labs**							
9	Dendrite and cell body	GFP	0-700 µm	30-120 mW	920 nm	Homebuilt system LotosScan 1.0	Gu *et al.* Rabies virus-based labeling of layer 6 corticothalamic neurons for two-photon imaging in vivo. iScience, 26, 106625 (2023). https://doi.org/10.1016/j.isci.2023.106625
10	Cell body	Cal 590	806 µm	120 mW	1070 nm	Homebuilt system Konnerth lab	Tischbirek *et al.* In Vivo Functional Mapping of a Cortical Column at Single-Neuron Resolution. Cell Reports 27, 1319-1326.e5 (2019). https://doi.org/10.1016/j.celrep.2019.04.007
11	Cell body	GCaMP6f	500 – 780 µm	50 – 75 mW	950 nm	Custom-built Sutter MOM	Augustinaite et al. Complementary Ca2+ Activity of Sensory Activated and Suppressed Layer 6 Corticothalamic Neurons Reflects Behavioral State. Current Biology 30, 3945-3960 (2020). https://doi.org/10.1016/j.cub.2020.07.069

We note that Ref. 8-11 were all biological papers, involving many imaging sessions and animals. The ability of well-designed two-photon fluorescence microscopes to routinely image synapses (Ref. 8, 620 µm max depth, 775 basal spines and 794 oblique spines from 4 animals imaged daily over 5-10 days) and cell bodies (Ref. 9-11 with 700 µm, 806 µm, and 780 µm max depth) at similar depths and excitation power to our data is well established experimentally.

The power ranges used in these publications were also consistent with our observation. Instead of increasing power by 10 or 30 folds to combat the 1000× signal drop predicted by the reviewer, the power increase from superficial brain to max depth was much less:

Ref. 5: 2.7× for 612 µm depth difference
Ref. 6: 4× for 600 µm depth difference
Ref. 7: 5.3× for 671 µm depth difference
Ref. 8: 1.7× for 320 µm depth difference
Ref. 9: 4× for 700 µm depth difference
Ref. 11: 1.5× for 270 µm depth difference

In summary, both our data and those in the literature indicate that fluorescence signal does not drop as fast with depth as claimed by the reviewer based on attenuation length.

We speculate that the discrepancy between the reviewer's theoretical prediction and experimental data in the literature may arise from two factors. The fluorescence collection paths of the microscopes used for attenuation length measurements might not have been optimized for collecting scattered fluorescence photons at large imaging depths. Brain-induced optical aberration also increases with image depth, artifactually reducing the fluorescence signal and attenuation length. Both factors may lead to an underestimation of the attenuation depth in vivo. Although resolving such discrepancy is of important scientific value, it is beyond the scope of our manuscript.

4. A minor point:
Please report the power throughput for the FACED module and the microscope separately. In addition, the authors need to report the intensity non-uniformity of FACED illumination line, which will also impact the recording SNR across the FOV.

We edited the Methods section to be "The power throughput of the FACED module is 25%." We decide not to provide the power throughput for the microscope, as different microscope systems and microscope objectives have different power throughputs. Providing the power throughput of FACED module itself is sufficient for interested readers to calculate the power throughput of their microscope systems after adding the FACED module.

For illumination profile, we added to the final section in Methods: "Experimentally, defining the peak illumination power along the FACED line scan as 1, illumination power along the FACED line scan had a mean of 0.80 and a standard deviation of 0.12, with minimal illumination around 0.5."

Please see our detailed responses below. Edits made to the manuscript are highlighted in yellow.

1. The quantification of the SNR of the voltage and Ca imaging.
For voltage imaging, the calculations of the SNR by the authors do not agree with our calculations. In our previous responses, we stated that the SNR of the voltage recording should be calculated directly from the photon counts per neuron per frame (F) and the measured (or reported) delta F/F for the probe. We calculated SNR = delta F/sqrt(F) = delta F/F * sqrt(F) = 20% [cited in the main text] * sqrt(300) [Supp Table 5, 50% percentile] ~= 3.5. However, the authors insisted on an SNR ~ 7.5 with the same definition.

SNR is a crucial quantity that determines the recording quality, and these two drastically different quantifications of SNRs could lead to entirely opposite understandings of the performance. We would like the authors to point out what makes up the difference in the SNR calculations.

The definition of SNR associated with the value 7.5 is NOT the same as the Reviewer's definition. In short, using the Reviewer's definition, we obtain a similar value of ~3.5. We used a different definition to address a spike-detection concern raised by the Reviewer in the first round of review, as detailed below. At the end of our response, please see our proposal to reconcile this discrepancy.

I. Why the misunderstanding?

This misunderstanding arose from the 1st round review, in which the Reviewer claimed that we "simply lower the spike detection threshold from SNR > 7.5 (FACED 1.0) to SNR > 2.5 (FACED 2.0, this work)".

In response to this comment, we first explained how we defined SNR in FACED 1.0 (blue boxes below are screenshots from previous rebuttal and manuscript):

Spike detection criteria in FACED 1.0 and FACED 2.0:

In FACED 1.0, spike detection was based on the signal-to-noise ratio (SNR), defined as $-\Delta F/\sqrt{F}$, where F represents baseline fluorescence and ΔF is the fluorescence change relative to baseline. Spikes were detected when SNR was above 7.5.

Throughout our manuscript, F is defined as the fluorescence pixel value (i.e., as digitized by our data acquisition system), not the number of photons that the Reviewer used in their definition. In retrospect, we should have made it clear that F is not the number of photons. However, both in our paper and in published literature, the value of F generally does not denote photon numbers (which usually use the symbol "N"); in Methods, we also described how F was extracted (by Volpy from an input image time-series).

But we agreed with the Reviewer that number of photons is more appropriate when comparing across different systems. This is why we provided photon counts in **Supplementary Table 5**. Furthermore, in **Supplementary Note 2.2** and **Supplementary Fig. 6**, we validated our two-step spike detection method by calculating its true positive and false positive rates using different number of photons per ROI:

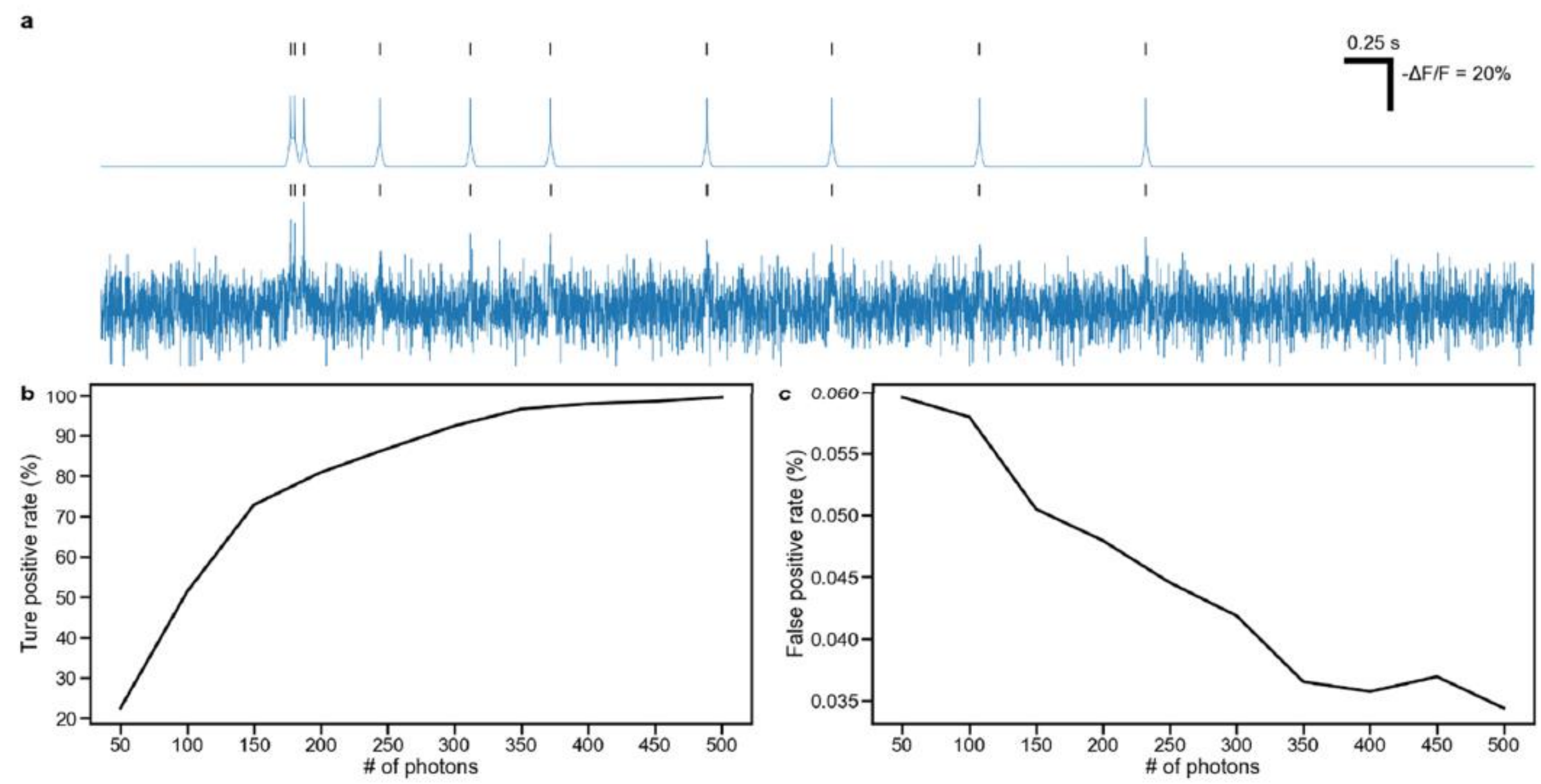

Supplementary Fig. 6. Validation of the spike detection method on simulated voltage traces. (**a**) Top trace: an example ground-truth voltage trace. Top black ticks: ground-truth spikes. Bottom trace: corresponding noise-added voltage trace with a SNR from 150 photons/frame. Bottom black ticks: spikes detected from noise-added voltage trace. (**b**) True positive rate of spike detection across different SNR conditions (SNR is quantified by number of photons per frame) (**c**) False positive rate of spike detection across different SNR conditions (SNR is quantified by number of photons per frame). For **b** and **c**, data point for each SNR condition is computed from 1000 simulated noisy traces with the number of spikes randomly selected (1 to 50) and randomly positioned within the trace duration (**Supplementary Codes**).

We showed that our spike detection method has satisfactory performance at 316 photos per ROI (or ~3.5 SNR using the Reviewer's definition):

> more accurate spike detection. At the average photon counts for neurons in Fig. 2 (316.2 photons per ROI, **Supplementary Table 5**), our method achieved >90% true positive rates and ~0.04% false positive rates (i.e., ~4 false spikes in 10,000 time points or a 12.5-s-long recording).

Since spike detection is a main goal of voltage imaging, we believe that the analysis in Supplementary Note 2.2 and Supplementary Fig. 6 already addressed the reviewer's concern on spike detection and is more informative than simply giving an average SNR value.

II. What about the SNR of 7.5?

To address the Reviewer's comment that we "simply lower the spike detection threshold from SNR > 7.5 (FACED 1.0) to SNR > 2.5 (FACED 2.0, this work)", we used the SNR definition in FACED 1.0 to calculate the SNR for FACED 2.0 data, which showed that FACED 2.0 and FACED 1.0 have similar SNR for spikes in **Supplementary Note 2.1**:

Here, we calculated the signal-to-noise ratio (SNR), defined as $-\Delta F/\sqrt{F}$, for spikes detected in **Fig. 2** and **Extended Data Fig. 11** using the above spike detection criterion (**Supplementary Fig. 3**). We found that 96.2 % and 75.1% of neurons, respectively, had their spike SNR values greater than 7.5.

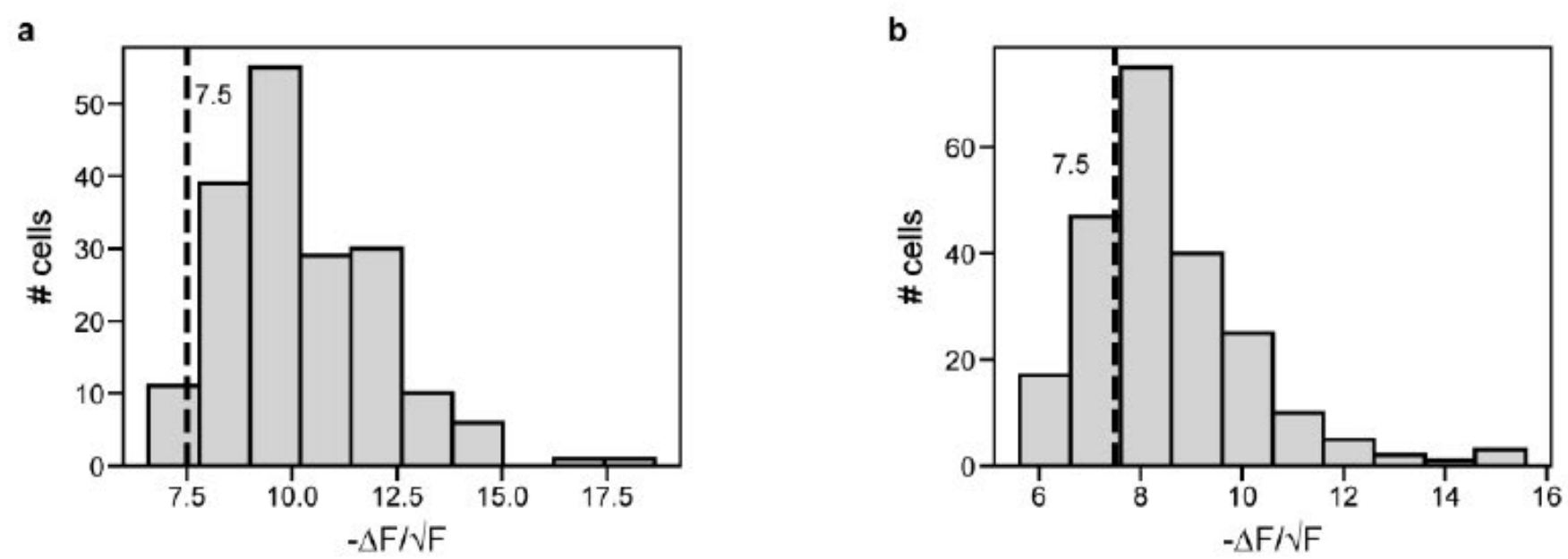

Supplementary Fig. 3. Histograms of the average $-\Delta F/\sqrt{F}$ of detected spikes for 182 neurons in (a) Fig. 2 and (b) 225 neurons in Extended Data Fig. 11. Dashed line: $-\Delta F/\sqrt{F} = 7.5$.

We then reanalyzed the voltage traces from **Fig. 2** and **Extended Data Fig. 11** using a $-\Delta F/\sqrt{F}$ thresholding criterion for spike detection, which we previously employed[2]. Here a spike was detected when the SNR value was greater than 7.5. This criterion produced quantitatively similar population activity characteristics to those in **Fig. 2** and **Extended Data Fig. 11** (**Supplementary Figs. 4,5**).

In other words, the SNR threshold of 7.5 using the definition of FACED 1.0 was only mentioned in **Supplementary Note 2.1** and its associated **Supplementary Fig. 3-5**, to address a specific comment of the Reviewer.

III. How to fix this?

We remove **Supplementary Note 2.1** and its associated **Supplementary Fig. 3-5**, which were included in the revision only to address Reviewer's specific concern on the difference of spike detection between FACED 1.0 and FACED 2.0. This way, we may avoid misleading general readers.

To better distinguish between fluorescence pixel values from number of photons, we added to Methods: "The imaging parameters, fluorescence pixel values, and estimation of number of photons are summarized in Supplementary Tables 2-6."

For volumetric Ca imaging, the SNR, or the d' value, as calculated in the previous response, is not sufficient for depths beyond 500 um (see DOI: https://doi.org/10.1016/j.bpj.2012.07.058 and https://doi.org/10.7554/eLife.53205).

We did not calculate d' values in our previous response. On Page 9 and 10 in the rebuttal file, we provided experimental evidence on our ability in imaging activity beyond 500 μm.

The authors claimed that the responses in calcium activities to the drifting grating stimuli are still visible to the algorithm and human eyes from a single trial which lasts 3-5 seconds or by averaging over multiple trials. This is true for repeating grating stimuli experiments, but for calcium imaging in general, the ability to reliably detect single, perhaps rarely repeatable activity events is still crucial. The FACED microscope presented here failed to demonstrate such a capability.

We already showed in our previous response that calcium transients were detectable from unaveraged single-trial data:

> The responses from these neurons during a total of ~60 min of recording were shown in **Extended Data Fig. 17** (neurons numbered the same way as the figure panel above):

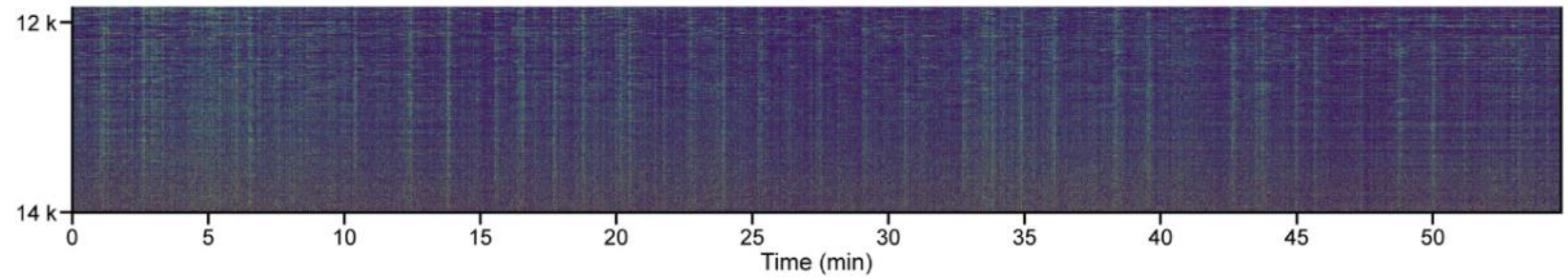

> In these traces, calcium activity from neurons at 500 µm and deeper were clearly visible.

<u>Finally, we note that the reviewer's concern pertains to a small subset of data in **Extended Data Figure 17** (specifically, neurons deeper than 500 µm).</u> We believe that our data fully support our conclusions regarding FACED's capabilities.

2. The improvement of FACED 2.0 vs FACED 1.0.
The authors replied that "the improvement from FACED 1.0 to FACED 2.0 was NOT about the increased number of independent pixels," but about enlarging the imaging FOV while sustaining the high axial resolution that is critical for high 2P fluorescence signals. Essentially, the larger imaging FOV comes at the expense of even higher pixel crosstalk (previously 2-ns interpixel delays and 1-ns interpixel delays now, compared to 3-ns to 4-ns fluorescence lifetime of common fluorophores), which effectively compromises the spatial resolution along the FACED scanning direction. While the authors argued in Supp Note 4 that the pixel crosstalk led to minimal contamination in neuronal activities, this feature/bug, unique to the FACED microscope, could preclude its use in the wider imaging applications.

We respectfully disagree that lifetime crosstalk limits FACED's use in broader imaging applications.

<u>As shown in **Supplementary Note 4**, lifetime crosstalk does not compromise population calcium and voltage imaging</u>—the focus of this paper, as reflected in its title, "FACED 2.0 enables large-scale voltage and calcium imaging in vivo." Imaging activity in densely labeled neurons account for most in vivo neuroscience imaging applications.

If the reviewer has concerns about our analyses in **Supplementary Note 4**, please state them explicitly. An assertion that "this feature/bug, unique to the FACED microscope, could preclude its use in the wider imaging applications" without supporting evidence does not enable a constructive response.

For the reason discussed above, statements in the main text like "FACED 2.0 achieves high-speed imaging without sacrificing spatial resolution" are not true for the FACED-scanning direction. Modifications of such statements are needed to avoid misleading messages.
We agree that the resolution along the FACED scanning direction is lower than standard two-photon fluorescence microscopy.

We have clarified in "FACED 2.0 enables high-throughput volumetric calcium imaging in the mouse and zebrafish larval brain in vivo" that "Importantly, FACED 2.0 does not sacrifice ~~spatial~~ axial resolution in

exchange for imaging throughput, unlike other high-throughput volumetric imaging methods with reduced ~~lateral and/or axial~~ resolution".

And in DISCUSSION: "Importantly, FACED 2.0 achieves high-speed imaging without sacrificing ~~spatial~~ axial resolution, with its high excitation NA leading to subcellular resolution laterally and cellular resolution axially."

3. The use of a constant excitation power throughout an imaging depth of ~ 750 um is a bad practice and not justifiable under any circumstance. It should be explicitly stated as such to discouraged others from doing the same.

Again, this comment pertains ONLY to the data in **Extended Data Figure 17**. While adjusting power with depth is generally a better approach, we respectfully disagree that keeping the power constant is "a bad practice" or "not justifiable under any circumstance." Many biological laboratories do not have Pockels cells on their microscopes. As long as the power does not cause photodamage, it is reasonable to keep the power constant across depth.

The authors chose not to vary the laser power with imaging depth because "doing so would require the inclusion and synchronization of a Pockel cell, which increases system cost and complexity." We believe many would agree that the integration of a Pockels cell is way less expensive or complex than building a FACED microscope. Many biological imaging labs have been equipped with Pockels cells in the system, and there exists commercial firmware that supports Pockels cell synchronization as a standard option. The authors went on to cite publications that discredit the need to greatly vary the excitation power with imaging depth. However, some of these examples evidently show that a greater change in laser power is preferred. For example, in the authors' own previous work (FACED 1.0 in vivo blood circulation imaging, Ref. 7 in the authors' rebuttal on page 11), they instructed in the methods that "Given the finite half-life of injected fluorescent dyes (~2 h for 70-kDa dyes) and tissue attenuation of the excitation light, we first collected data from the deepest blood vessels and shifted the imaging plane gradually toward the brain surface." This indicates that the deeper blood vessels were inherently brighter than the superficial ones when imaging acquisition occurred. With a 5.3x for 671 um depth difference (should it be ~ 530 um because the deepest video was obtained from a different mouse/experiment?), the signal level or SNR of the videos/kymographs in deeper layers is visibly lower than that in shallow layers. Therefore, the drop in signal level in deeper regions, compounded with the fact that deeper blood vessels were inherently brighter, suggests that a greater difference in laser power across imaging depth is required to reach approximately uniform signal levels.
We also understand that the authors are able to integrate a Pockels cell into the system. Imaging a volume as thick as > 700 um without varying the laser power is a bad idea, and should not be encouraged or emulated.

We included 11 references on page 11 in our previous rebuttal to show that the reviewer's assertions (that two-photon signal at depth should be attenuated by ~1,000×) were inaccurate. It was not intended to encourage users to avoid using a Pockels cell or to refrain from adjusting excitation power when that capability is available. Nowhere in the manuscript do we make such statements.

We have added a sentence in the Methods: "Although we used constant excitation power in volumetric imaging mode, an optimal implementation should adjust excitation power with imaging depth (e.g., using a Pockels cell)."

To conclude, although the authors have demonstrated specific biological imaging experiments using FACED 2.0, judging from the angle of technology development, we don't see a substantial improvement of FACED 2.0 over FACED 1.0. The main technical disadvantages of the FACED method (i.e., the pixel cross talk due to the short time delay between adjacent pixels) is not addressed at all and could limit its adoption to the wider imaging community.

Through multiple rounds of review, we have repeatedly explained and demonstrated that pixel crosstalk does not affect the accuracy of large-scale voltage and calcium imaging.

Regarding whether FACED 2.0 represents a substantial improvement over FACED 1.0: FACED 1.0, with its much smaller FOV, cannot acquire the data presented here. We have demonstrated advances in FACED 2.0 sufficient to meet the publication criteria of a Brief Communication.

The data included in our manuscript represent a lower bound of FACED's current capabilities. New voltage sensors with improved performance (e.g., higher ΔF/F for action potentials) than JEDI-2P are becoming available. In collaboration with voltage sensor developers, we are using FACED to acquire population voltage datasets for six different voltage sensors (two manuscripts under review, two to be submitted soon, and two in preparation). With these sensors, we have already achieved even larger FOV and higher SNR with FACED 2.0. Although we cannot include these data in the current manuscript because these sensors are unpublished, we would be happy to provide representative examples for editorial review.

In summary, FACED 2.0 is, to our knowledge, the most powerful method for population voltage imaging to date; its impact in neuroscience will be further amplified by the next generation of voltage sensors and will continue to grow in the coming years.
